# m^1^A-mediated regulation of BIRC2 mRNA stability drives apoptosis evasion and tumor progression in liver cancer

**DOI:** 10.1038/s41419-026-08731-z

**Published:** 2026-04-16

**Authors:** Yingmin Wu, Shenjie Zhang, Shilong Zhang, Jieyu Lu, Yuntao Yang, Zhirui Zeng, Shan Lei, Rui Mi, Yewei Zhang, Lichen Ge, Tengxiang Chen, Haiyang Li

**Affiliations:** 1 Department of Surgery, Affiliated Hospital of Guizhou Medical University, Guiyang, China; 2 Department of Physiology and Pathophysiology, School of Basic Medical Sciences, Guizhou Medical University, Guiyang, China; 3 Transformation Engineering Research Center of Chronic Disease Diagnosis and Treatment, Guizhou Medical University, Guiyang, China; 4 Guizhou Institute of Precision Medicine, Affiliated Hospital of Guizhou Medical University, Guiyang, China; 5 Department of Breast Thyroid Surgery, The Third People’s Hospital of Chengdu, The Affiliated Hospital of Southwest Jiao tong University, Chengdu, China; 6 Department of Laboratory Medicine, Third Affiliated Hospital of Sun Yat-sen University, Guangzhou, China

**Keywords:** Apoptosis, RNA decay, Cancer epigenetics

## Abstract

Liver cancer remains one of the leading causes of cancer-related mortality worldwide, with its progression driven by uncontrolled cell proliferation and evasion of apoptosis. N1-methyladenosine (m^1^A) is a prevalent RNA modification implicated in cancer progression, yet its role in liver cancer remains unclear. Here, we report a significant reduction in m^1^A levels in liver cancer tissues, which contributes to apoptosis evasion in liver cancer cells. We demonstrate that ALKBH3, an m^1^A demethylase, regulates apoptosis by modulating BIRC2 expression. Specifically, ALKBH3 depletion destabilizes BIRC2 mRNA by promoting its degradation, facilitated by m^1^A modifications at positions A98/99/100 in the 5'-UTR of BIRC2. These modifications enhance the interaction between BIRC2 mRNA and the YTHDF3/CNOT1–XRN2 complex, thereby driving mRNA degradation. In vitro, in vivo, and clinical analyses validate the critical role of the m^1^A/BIRC2 axis in regulating apoptosis and tumor progression in liver cancer. Our findings underscore the therapeutic potential of targeting the m^1^A/BIRC2 axis to overcome apoptosis resistance in liver cancer, offering new avenues for intervention in this malignancy.

## Introduction

Primary liver cancer is the seventh most commonly diagnosed cancer worldwide and the second leading cause of cancer-related mortality, with both incidence and mortality rates continuing to show a significant upward trend across diverse populations [1]. As the predominant form of primary liver malignancy, accounting for approximately 85% of all cases, it poses a significant clinical and socioeconomic burden, especially in regions with high prevalence of hepatitis B or C virus infection [2, 3]. Despite advances in treatment options, the prognosis for liver cancer remains poor, largely due to late diagnosis and an incomplete understanding of its molecular pathogenesis. Therefore, elucidating novel regulatory mechanisms driving liver cancer progression is urgently needed.

Emerging evidence highlights the pivotal role of epitranscriptomic reprogramming in liver cancer development and metastatic progression [4]. Among various RNA modifications, N1-methyladenosine (m^1^A) has garnered increasing attention as a dynamic post-transcriptional regulator [5]. This reversible modification, characterized by methyl group addition at the adenosine N1 position, induces local RNA structural perturbations by disrupting canonical base pairing [6, 7]. Similar to the well-characterized N6-methyladenosine (m^6^A) modification, m^1^A is dynamically regulated by methyltransferases (writers: TRMT6/61A/61B/10C, NML), demethylases (erasers: ALKBH1/3/7, FTO), and recognition proteins (readers: YTHDF1–3, YTHDC1) [7–10]. Notably, ALKBH3 serves as the principal eraser responsible for m^1^A demethylation on mRNA [10]. These regulatory components collectively orchestrate m^1^A-mediated control of mRNA splicing [11], structural conformation [6], translational efficiency [5, 7, 11, 12], and degradation kinetics [13–15].

Dysregulation of m^1^A homeostasis has been implicated in multiple oncogenic processes. Our previous investigations demonstrated that m^1^A modulates tRNA-derived fragment (tDR) biogenesis to regulate cancer cell proliferation, clonogenicity, invasiveness, and apoptosis [9], while also suppressing glycolytic metabolism through ATP5D regulation [16]. In the context of liver cancer, disruption of the TRMT6–TRMT61A complex formation inhibits tumor self-renewal capacity [17], and m^1^A-related regulators show diagnostic and prognostic potential [18, 19]. Nevertheless, the precise mechanistic links between m^1^A dysregulation and the aggressiveness of liver cancer remain largely elusive.

A critical barrier in liver cancer management lies in the ability of cancer cells to evade apoptosis—a fundamental mechanism that sustains malignant proliferation and confers therapeutic resistance [20–22]. Restoring apoptotic sensitivity represents a promising therapeutic strategy [23–25], though its clinical translation requires a deeper understanding of the underlying molecular evasion mechanisms. The inhibitor of apoptosis protein Baculoviral IAP repeat-containing 2 (BIRC2, also known as c-IAP1), which is overexpressed in 40% of breast cancers and associated with T cell resistance [26, 27], has emerged as a key mediator of apoptotic escape in liver cancer [28–30]. Our recent work [31] corroborates prior findings [32–34] that epigenetic regulation, particularly through ubiquitination-dependent BIRC2 stabilization, facilitates apoptotic evasion. However, the potential involvement of epitranscriptomic mechanisms in this process remains unexplored.

In this study, we identified significant m^1^A hypomodification in liver cancer specimens, which correlated with advanced disease progression. Functional studies demonstrated that ALKBH3 knockdown promotes apoptosis, while its reconstitution restores apoptotic resistance. Mechanistically, we revealed that m^1^A enrichment in the 5′-UTR of BIRC2 mRNA facilitates transcript degradation through the YTHDF3/CNOT1–XRN2 complex. These findings establish a novel regulatory axis linking m^1^A-dependent RNA epitranscriptomic modification with apoptotic evasion in liver cancer pathogenesis.

## Materials and methods

### Clinical specimens and data acquisition

Paired tumor and adjacent normal tissue samples were collected from 15 treatment-naïve liver cancer patients who underwent surgical resection at Guizhou Medical University Hospital between 2022 and 2025. Detailed clinical characteristics of the patients are provided in Supplementary Table S1. All fresh specimens were immediately snap-frozen in liquid nitrogen and stored at −80 °C until biomolecular extraction.

Publicly available liver cancer (LIHC) data were obtained from The Cancer Genome Atlas (TCGA) portal (https://portal.gdc.cancer.gov/) [35], including RNA‑seq expression values (in TPM and FPKM formats), copy number variation (CNV) data, and clinical annotations from 371 hepatocellular carcinoma (HCC) cases and 50 normal liver tissue samples. Bioinformatic analyses were performed using R version 4.2.1, with the following packages: limma, ConsensusClusterPlus, and ggplot2.

### m^1^A RIP (MeRIP)-qPCR

The MeRIP-qPCR assay was performed based on a previously described protocol with minor modifications [16]. Total RNA was extracted using TRIzol reagent. A total of 50 ng of RNA was saved as input control, and the remainder (2 μg) was subjected to immunoprecipitation (IP) using an anti-m^1^A antibody (MBL) in RIP buffer (150 mM NaCl, 0.1% NP-40, 10 mM Tris [pH 7.4], and 100 U of RNase inhibitor) to enrich m^1^A-modified RNAs. IgG was used as a negative control. Immunoprecipitation was performed using Dynabeads® Protein A (Thermo Fisher Scientific), and bound RNAs were eluted three times with elution buffer (5 mM Tris-HCl [pH 7.5], 1 mM EDTA [pH 8.0], 0.05% SDS, and 20 mg/mL Proteinase K). The eluted RNA was ethanol-precipitated and quantified using the Qubit® RNA HS Assay Kit (Thermo Fisher Scientific).

For qPCR analysis, 2 ng of input RNA or m^1^A-IP RNA was used as template. GAPDH mRNA, which lacks m^1^A peaks based on m^1^A profiling data [16], served as the internal control for input normalization. For experiments involving fragmented RNA, total RNA was fragmented using fragmentation reagents (Thermo Fisher Scientific) according to the manufacturer’s instructions. All primers used for amplifying regions of BIRC2 mRNA—including the 5′‑UTR, CDS, 3′‑UTR, and seven subregions of the 5′‑UTR (U1 to U7)—are listed in Supplementary Table S3.

### Pull-down and mass spectroscopy analysis

A total of 10^7^ cells were pelleted and lysed in 400 μL of cell lysis buffer for Western blot and IP (Beyotime), supplemented with protease inhibitor cocktail. The lysate was clarified by centrifugation and pre-cleared using 20 μL of Dynabeads™ Protein G (Thermo Fisher Scientific) for 2 h at 4 °C. The pre-cleared lysate was then incubated overnight at 4 °C with Dynabeads conjugated with either YTHDF3 antibody or IgG (as a negative control). After incubation, the beads were washed three times with lysis buffer, and bound proteins were eluted by boiling in 30 μL of 1× SDS loading dye.

A 5 μL aliquot of the eluted proteins was resolved by sodium dodecyl sulfate–polyacrylamide gel electrophoresis (SDS–PAGE) and visualized by silver staining. The remaining 25 μL were submitted to Shanghai OE Biotech Co., Ltd. for liquid chromatography–tandem mass spectrometry (LC–MS/MS) analysis. The resulting protein identifications are provided in Supplementary Table S4 and the mass spectrometry proteomics data have been deposited to the National Genomics Data Center (https://ngdc.cncb.ac.cn/) [36] via the OMIX with the dataset identifier OMIX01275. Gene Ontology (GO) enrichment analysis of identified proteins was performed using g: Profiler (https://biit.cs.ut.ee/gprofiler) [37] to assess pathway associations.

### Database analysis

Expression levels of BIRC2, ALKBH3, NFKB1, and YTHDF3 in LIHC cohorts were analyzed using data from TCGA [35]. The correlation between BIRC2 and ALKBH3 expression was assessed using LinkedOmics (http://www.linkedomics.org/login.php) [38], a publicly available platform providing multi-omics data across 32 TCGA cancer types.

The prognostic significance of ALKBH3, BIRC2, NFKB1, and YTHDF3 expression in patients with liver cancer was evaluated with the Kaplan–Meier plotter tool (http://kmplot.com/analysis/) [39]. Survival curves were compared using the log-rank test, and hazard ratios with 95% confidence intervals are reported.

### Statistical analyses

Data are presented as mean ± standard deviation (SD) from at least three independent biological replicates. For comparisons between two groups, a two-tailed unpaired Student’s *t*-test was used. For comparisons among three or more groups, one-way or two-way analysis of variance (ANOVA) was performed, followed by Bonferroni’s post hoc test for multiple comparisons. All tests were two-sided and conducted using SPSS version 16.0 for Windows. A *P*-value of less than 0.05 was considered statistically significant. Significance levels are denoted as follows: *p < 0.05, **p < 0.01, ***p < 0.001, and ****p < 0.0001; ns indicates not significant.

## Results

### Reduced m^1^A levels in liver cancer and its negative association with disease progression

**Fig. 1:**
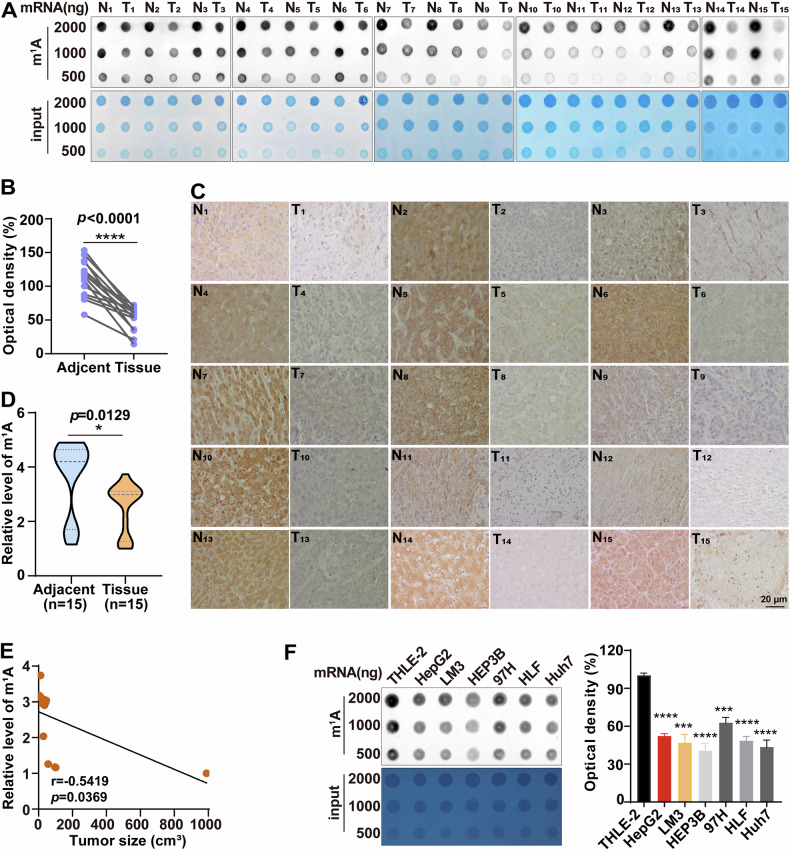
Reduced m^1^A levels in liver cancer and its negative association with disease progression. **A**, **B** m^1^A mRNA levels in paired human liver cancer tissues and adjacent normal tissues (*n* = 15 patients) were analyzed by dot-blot assay (**A**). Signal intensities were quantified using ImageJ, normalized to total mRNA stained with methylene blue, and are shown in (**B**). **C**, **D** Representative IHC images of m^1^A in paraffin-embedded sections from 15 paired liver cancer tissues and adjacent normal tissues (**C**). m^1^A staining intensity was semiquantitatively scored by ImageJ (**D**). Scale bar: 20 µm (×400). **E** Correlation between m^1^A dot-blot signal intensity (normalized to adjacent normal tissue) and maximal tumor diameter in 15 liver cancer patients. **F** mRNA m^1^A levels were measured via dot-blot in multiple liver cancer cell lines (HepG2, HCCLM3, HEP3B, MHCC-97H, HLF, Huh7) and the normal hepatocyte line THLE-2 (left). Signal intensities were quantified using ImageJ and normalized to total mRNA stained with methylene blue (right). Data are presented as mean ± SD. Statistical analyses: **B**, **D** paired two-tailed Student’s *t*-test; (**E**) Spearman’s rank correlation; **F** one-way ANOVA with Tukey’s post-hoc test for multiple comparisons vs. THLE-2. *p < 0.05, **p < 0.01, ***p < 0.001, ****p < 0.0001; ns, not significant.

Our previous study [18] indicated that m^1^A methylation levels in mRNA are lower in liver cancer tissues than in adjacent non-cancerous tissues and are inversely correlated with tumor aggressiveness, including overall survival (OS), tumor size, and Alpha-Fetoprotein (AFP) levels. To further investigate the role of m^1^A modification in liver cancer progression, we compared m^1^A levels in mRNA extracted from 15 pairs of liver cancer tissues and adjacent non-cancerous mucosa (Supplementary Table S1). Levels of m^1^A modification were detectable in all tumor and normal tissue samples analyzed. Dot-blot assays revealed a significant reduction in m^1^A levels in all tumor tissues compared with adjacent normal tissues, with the average levels of m^1^A modification in tumor tissues being more than 50% lower than those in adjacent normal mucosa (Fig. 1A, B; *p* < 0.0001). Immunohistochemistry (IHC) confirmed these findings (Fig. 1C, D; *p* = 0.0129), and a negative correlation was observed between m^1^A levels and tumor size (Fig. 1E, *p* = 0.0369). However, no significant differences in m^1^A levels were detected across age, gender, TNM stage, or disease grade (data not shown), which may be attributed to the limited sample size.

Additionally, we examined genetic alterations in m^1^A-related genes (including TRMT6/61 A, TRMT61B, TRMT10C, ALKBH1, ALKBH3, YTHDF1, YTHDF2, YTHDF3, and YTHDC1) in liver hepatocellular carcinoma (LIHC) datasets from TCGA. A significant upregulation of these genes was observed in liver cancer tissues compared to normal liver tissues (Fig. S1A, *p* < 0.05 for all). Further analysis revealed a robust network of m^1^A-regulated gene interactions, including correlations within functional categories and among writers, erasers, and readers of m^1^A marks. Multivariate Cox regression analysis identified high expression levels of m^1^A regulators as significant risk factors for prognosis in liver cancer patients (Fig. S1B). However, no associations were found between expression levels of individual m^1^A regulators and clinical parameters such as tumor grade, clinical stage, or survival status (Fig. S1C). Collectively, m^1^A regulators show coordinated dysregulation and serve as independent prognostic markers in liver cancer, functioning through synergistic networks rather than through individual associations with clinicopathological traits.

Moreover, m^1^A levels were assessed in multiple liver cancer cell lines (HepG2, LM3, HEP3B, MHCC-97H, HLF, and Huh7) and the normal liver cell line THLE-2. In all tested liver cancer cell lines, m^1^A levels were significantly lower compared to THLE-2 cells. The HEP3B cell line, known for its high tumorigenicity and metastatic potential, showed the lowest m^1^A levels (Fig. 1F). Together, these results underscore reduced m^1^A modification of mRNA in liver cancer cells and tissues and its negative correlation with disease progression.

### m^1^A promotes apoptosis and suppresses proliferation in liver cancer cells

**Fig. 2:**
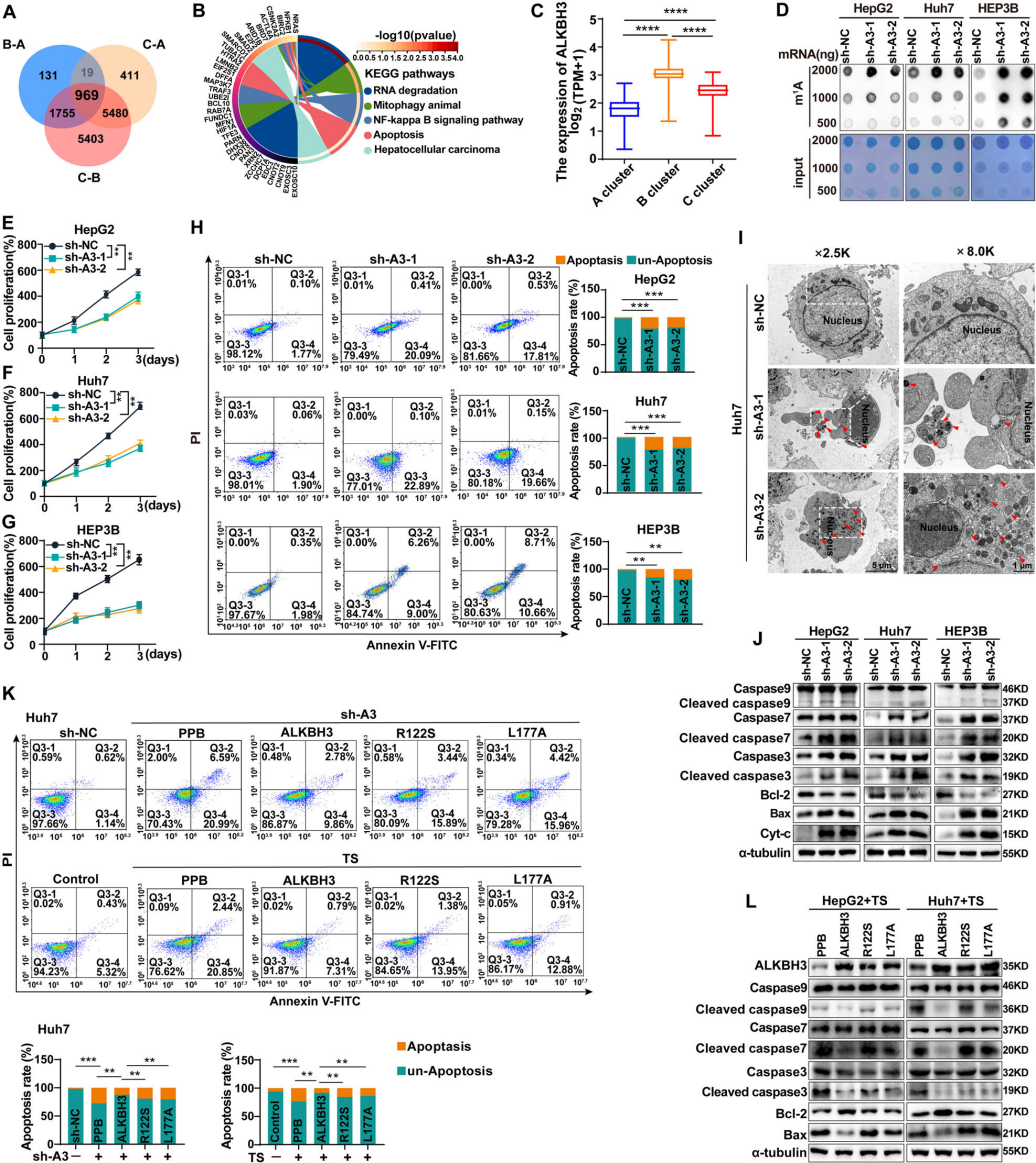
m^1^A promotes apoptosis and suppresses proliferation in liver cancer cells. **A** Venn diagram showing the intersection of differentially expressed genes (DEGs) among three molecular clusters (Cluster A, B, and C) identified from TCGA-LIHC transcriptomic data. **B** Gene Set Variation Analysis (GSVA) enrichment scores for apoptosis-related pathways across Cluster A, B, and C. **C** ALKBH3 mRNA expression levels across the three clusters. Data are derived from RNA-seq analysis of TCGA-LIHC samples (*n* = 371 tumor tissues). **D** Dot-blot analysis of m^1^A levels in HepG2, Huh7, and HEP3B cells stably transduced with shALKBH3 (sh-A3) or non-targeting control (sh-NC). Cell proliferation measured by CCK-8 assay in sh-A3 and sh-NC cells of HepG2 (**E**) Huh7 (**F**) and HEP3B (**G**). **H** Apoptosis assessment in sh-A3 and sh-NC HepG2, Huh7, and HEP3B cells was assessed by Annexin V-FITC/PI staining and flow cytometry. Representative flow cytometry plots (left) and quantitative analysis of the total apoptosis rate (right) are shown. **I** Transmission electron microscopy (TEM) images of sh-A3 and sh-NC Huh7 cells. Apoptotic bodies are indicated by red arrows. Scale bars: 5 μm (×2500), 1 μm (×8000). Images are representative of three independent experiments. **J** Western blot analysis of apoptosis-related proteins (Cleaved Caspase-3, Cleaved Caspase-9, Bcl-2, Bax) in sh-A3 and sh-NC cells. α-Tubulin served as a loading control. (K) Apoptosis levels assessed by flow cytometry: (upper) sh-A3 Huh7 cells transfected with the indicated constructs for 48 h; (lower) Huh7 cells were transfected with the indicated constructs for 48 h, followed by treatment with TS for 6 hours. Representative flow cytometry plots (upper) and quantitative analysis of the total apoptosis rate (lower) are shown. **L** Western blot analysis of apoptosis-related proteins in HepG2 and Huh7 cells treated as in (**K**, lower panel). Data are presented as mean ± SD. Statistical analyses: **B** one-way ANOVA with Tukey’s post-hoc test (*n* = 3 independent bioinformatic cohorts); **C** one-way ANOVA; **D** two-tailed unpaired *t*-test (*n* = 3); **E**–**G** two-way ANOVA with Bonferroni correction (*n* = 3 independent experiments); (H) unpaired *t*-test (*n* = 3); **K** one-way ANOVA with Tukey’s test (*n* = 3). *p < 0.05, **p < 0.01, ***p < 0.001, ****p < 0.0001; ns, not significant.

The m^1^A modification machinery, particularly the demethylase ALKBH3 as a key eraser [40, 41], dynamically regulates RNA metabolism and cellular signaling. To systematically dissect the functional landscape of m^1^A modification in liver cancer, we performed consensus clustering of 10 m^1^A regulators across 371 TCGA-LIHC patients, stratifying them into three molecular subtypes (Clusters A-C) with distinct clinical outcomes (Fig. S2A–D). Principal component analysis (PCA) confirmed distinct transcriptomic profiles among these subgroups (Fig. S2E), and intersection analysis identified 969 consensus differentially expressed genes (DEGs) (Fig. 2A).

Gene Set Variation Analysis (GSVA) revealed coordinated activation of apoptosis-related pathways alongside dysregulation of mitophagy and NF-κB signaling (Fig. 2B and Supplementary Table S5). The apoptosis signature encompassed both pro-apoptotic effectors (DFFA, HTRA2) and anti-apoptotic regulators (BIRC2, NFKB1), suggesting that m^1^A exerts bidirectional control over cell death programs. Notably, mitophagy-related genes such as FUNDC1 and RAB7A—critical for mitochondrial quality control—exhibited subtype-specific expression patterns, potentially linking m^1^A-modulated mitochondrial homeostasis to apoptotic susceptibility. Concurrently, NF-κB pathway components (e.g., TRAF3, BCL10) showed cluster-dependent dysregulation, implying m^1^A-dependent crosstalk between inflammatory signaling and apoptosis evasion. Strikingly, ALKBH3 emerged as the most differentially expressed m^1^A eraser across clusters (Fig. 2C; B > C > A, *p* < 0.0001) and displayed strong co-expression correlations with apoptosis-related DEGs (Figures S2F, G; BIRC2: r = 0.239, *p* < 0.001; NFKB1: r = 0.315, *p* < 0.001). This multi-omics convergence suggests that ALKBH3-mediated m^1^A demethylation may orchestrate apoptotic balance through mitochondrial integrity and NF-κB inflammatory signaling.

To dissect the functional role of ALKBH3-mediated m^1^A demethylation in apoptosis regulation, we established stable ALKBH3-knockdown models in three liver cancer cell lines (HepG2, Huh7, and HEP3B) using validated shRNAs (sh-A3-1 and sh-A3-2; Fig. S3A, B) [16, 18]. Dot-blot analysis showed that m^1^A levels were significantly higher in ALKBH3-knockdown cells than in control cells (Figs. 2D and S3C). ALKBH3 knockdown inhibited cell growth (Fig. 2E–G) and colony formation (Fig. S3D), while promoting apoptosis in liver cancer cells (Fig. 2H). Similarly, siRNA-mediated ALKBH3 knockdown in HepG2 and Huh7 cells resulted in significantly increased apoptosis rates compared with negative controls (Fig. S3E–G). Transmission electron microscopy (TEM) of shALKBH3 Huh7 cells revealed apoptotic vesicles, nuclear chromatin condensation, and cytoplasmic vacuolization, confirming enhanced apoptosis (Fig. 2I). Western blot analysis showed that ALKBH3 knockdown led to upregulation of apoptotic markers (cleaved caspases‑9, ‑7, and ‑3, Bax, and Cytochrome c) and downregulation of the anti-apoptotic protein Bcl-2 (Figs. 2J and S3H).

Conversely, in HepG2 and Huh7 cells treated with the apoptosis inducer TNF‑α + SM‑164 (TS) for 6 h, overexpression of wild-type ALKBH3 — but not the catalytically inactive mutants R122S and L177A — reduced mRNA m^1^A levels (Fig. S4A, B), promoted cell proliferation (Fig. S4C, D), and enhanced colony formation (Fig. S4E). Furthermore, overexpression of wild-type ALKBH3, though not the R122S or L177A mutants, counteracted apoptosis induced by ALKBH3 knockdown or TS treatment [42] (Figs. 2K and S4F). Western blot analysis further confirmed that only wild-type ALKBH3 reversed the apoptotic marker changes induced by TS treatment (Figs. 2L and S4G). These results indicate that ALKBH3 promotes apoptosis evasion in liver cancer through its m^1^A demethylase activity.

### BIRC2 is involved in m^1^A-regulated apoptosis in liver cancer cells

**Fig. 3:**
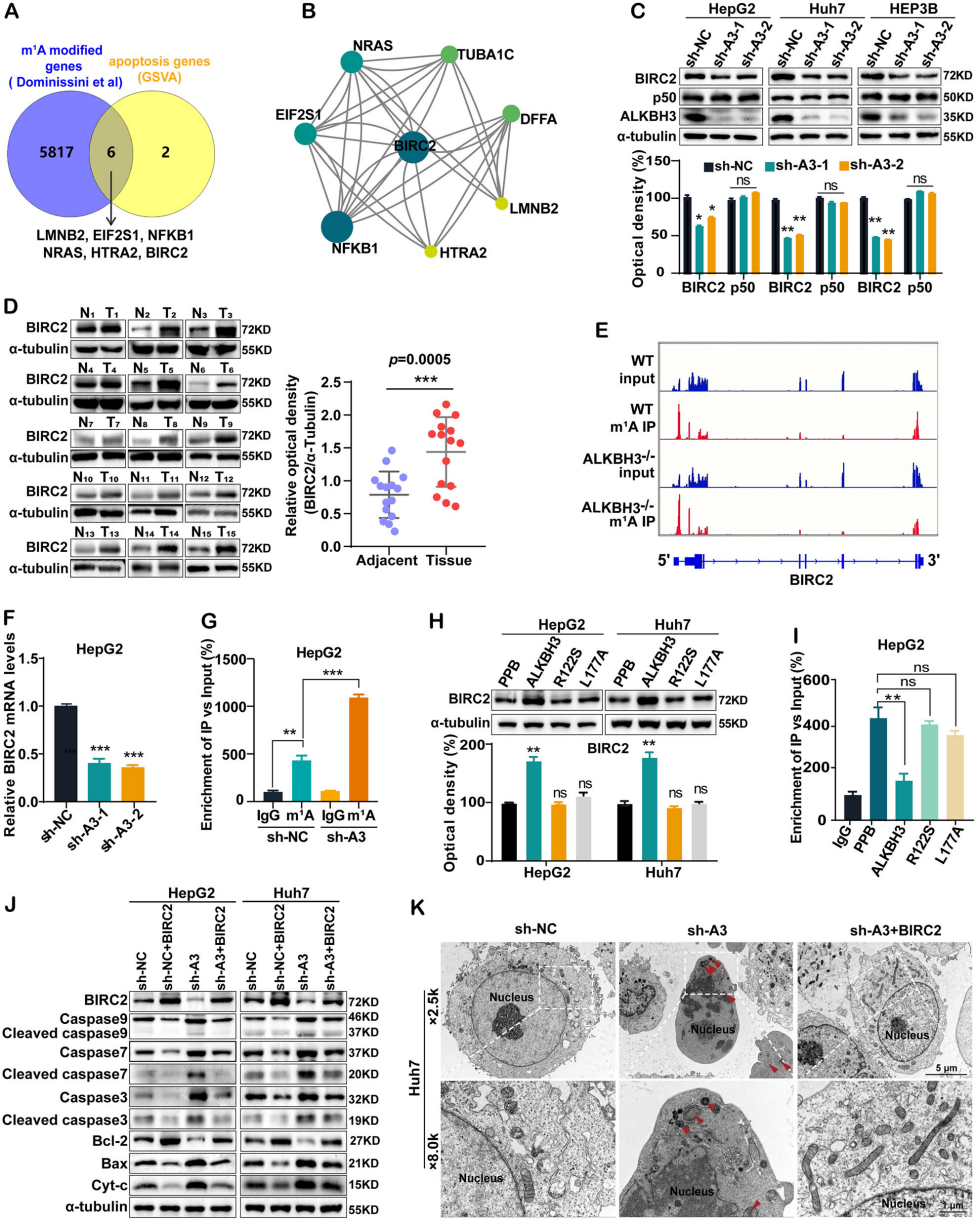
BIRC2 is involved in m^1^A-regulated apoptosis in liver cancer cells. **A** Venn diagram showing the overlap between apoptosis-related genes identified by GSVA and m^1^A-enriched transcripts from m^1^A-seq in HepG2 cells [5]. **B** Protein-protein interaction network of apoptosis-related proteins (from GSVA) and m^1^A regulators, constructed using STRING database. Nodes represent proteins, edges indicate functional associations. **C** Western blot analysis of BIRC2 and NFKB1 (p50) expression in HepG2, Huh7, and HEP3B cells stably transduced with shALKBH3 (sh-A3) or non-targeting control (sh-NC) (upper). Signal intensities were quantified using ImageJ and normalized to α-Tubulin (lower). **D** BIRC2 protein levels in 15 paired human liver cancer tissues and adjacent normal tissues (left). Signal intensities were quantified using ImageJ and normalized to α-Tubulin (right). **E** m^1^A RIP-seq profiles showing m^1^A peak enrichment in the 5′-UTR of BIRC2 mRNA in wild-type (WT) versus ALKBH3-knockout HeLa cells [16]. **F** Relative BIRC2 mRNA levels in sh-A3 and sh-NC HepG2 cells measured by RT-qPCR and normalized to GAPDH. **G** m^1^A RIP-qPCR analysis of BIRC2 mRNA enrichment in sh-A3 versus sh-NC HepG2 cells. **H** Western blot of BIRC2 in HepG2 and Huh7 cells transfected with empty vector (PPB), ALKBH3, ALKBH3-R122S, or ALKBH3-L177A for 48 h (upper). Signal intensities were quantified using ImageJ and normalized to α-Tubulin (lower). **I** m^1^A RIP-qPCR showing BIRC2 mRNA enrichment in HepG2 cells transfected as in (H). **J** Western blot analysis of apoptosis-related proteins (Cleaved Caspase-3, Cleaved Caspase-9, Bcl-2) in sh-A3 HepG2 and Huh7 cells rescued with pcDNA3.1-BIRC2 or empty vector. α-Tubulin was used as a loading control. **K** TEM images in sh-A3 and sh-NC Huh7 cells with or without BIRC2 overexpression. Apoptotic bodies are indicated by red arrows. Scale bars: 5 μm (×2500), 1 μm (×8000). Data are presented as mean ± SD. Statistical analyses: **D** paired two-tailed *t*-test (*n* = 15 patients); **F** unpaired *t*-test (*n* = 3); **G** unpaired *t*-test (*n* = 3); **I** one-way ANOVA with Tukey’s test (*n* = 3). *p < 0.05, **p < 0.01, ***p < 0.001, ****p < 0.0001; ns, not significant.

To identify key mediators of m^1^A methylation-induced apoptosis in liver cancer cells, we leveraged previous m^1^A sequencing (m^1^A-seq) data [5, 7, 12] and analyzed the STRING protein association network (string-db.org). We first examined whether the apoptosis-related genes identified in our GSVA enrichment analysis were subject to m^1^A modification. By comparing these genes with previously identified m^1^A-modified genes in HepG2 cells, we identified six overlapping candidates (Fig. 3A; for the complete list of m^1^A-modified genes, see Supplementary Table S6). Functional protein association network analysis revealed that BIRC2 and NFKB1 (p50) showed the strongest connections to m^1^A-regulated genes (Fig. 3B). Analysis of TCGA-LIHC data further demonstrated that BIRC2 expression, but not NFKB1, was significantly elevated in primary tumors compared with normal liver tissues (Fig. S5A, B). Moreover, elevated BIRC2 expression correlated with poorer survival outcomes in liver cancer patients (Fig. S5C, D). Furthermore, ALKBH3 knockdown significantly reduced BIRC2 protein levels without affecting p50 expression (Fig. 3C). Together, these findings suggest that BIRC2 may play a pivotal role in m^1^A-regulated apoptosis in liver cancer.

BIRC2 is known to function as an apoptosis inhibitor, contributing to apoptotic evasion and multidrug resistance in tumor cells [43]. We observed significantly higher BIRC2 expression in liver cancer tissues (*n* = 15) than in adjacent normal tissues (*n* = 15) (Fig. 3D). This upregulation was further validated in multiple liver cancer cell lines, where both BIRC2 mRNA (Fig. S5E) and protein (Fig. S5F) levels were consistently elevated compared with the normal liver cell line THLE-2. Analysis of m^1^A-seq data [16] revealed significant m^1^A enrichment in the 5′ untranslated region (5′-UTR) of BIRC2 in HeLa cells. This enrichment was markedly increased in ALKBH3-deleted HeLa cells relative to controls (Fig. 3E), a pattern consistent with observations in HepG2 cells [5] (Fig. S5G). Furthermore, ALKBH3 knockdown in HepG2, Huh7, and HEP3B cells led to reduced BIRC2 mRNA levels (Figs. 3F and S5H). Quantitative PCR (qPCR) analysis following m^1^A immunoprecipitation confirmed over fourfold enrichment of m^1^A in BIRC2 mRNA in HepG2 cells, with further increases upon ALKBH3 knockdown (Fig. 3G). These results were consistent in Huh7 cells (Fig. S5I). Importantly, no comparable enrichment was observed for NFKB1 (Fig. S5J), suggesting that NFKB1 is not regulated by ALKBH3-mediated m^1^A modification.

ALKBH3 can demethylate m^1^A and m^3^C in RNA and single-stranded DNA, respectively [9, 44]. However, further investigation revealed no significant m^3^C pull-down enrichment or upregulation of m^3^C modification in BIRC2 mRNA upon ALKBH3 knockdown in HepG2 cells (Fig. S5K), indicating that ALKBH3 regulates BIRC2 in an m^1^A-dependent rather than m^3^C-dependent manner. And overexpression of wild-type ALKBH3, but not the catalytically inactive mutants R122S or L177A, led to a marked increase in BIRC2 protein expression (Fig. 3H). In addition, ALKBH3 overexpression, though not the mutants, significantly reduced m^1^A methylation of BIRC2 mRNA in HepG2 cells (Fig. 3I).

To further investigate the role of BIRC2 in m^1^A-regulated apoptosis, we transfected ALKBH3-knockdown and control HepG2 and Huh7 cells with BIRC2 expression constructs (Fig. S6A). BIRC2 overexpression effectively reversed the ALKBH3-knockdown-induced suppression of proliferation (Fig. S6B) and colony formation (Fig. S6C), as well as the increase in apoptosis (Fig. S6D). Furthermore, BIRC2 overexpression attenuated the ALKBH3-knockdown-mediated upregulation of apoptotic markers (cleaved caspases‑9, ‑7, and ‑3, Bax, and Cytochrome c) and the downregulation of Bcl-2 expression (Figs. 3J and S6E). Transmission electron microscopy (TEM) corroborated these findings, showing reduced apoptosis in ALKBH3-knockdown Huh7 cells upon BIRC2 overexpression (Fig. 3K). Collectively, these data demonstrate that BIRC2 is a key effector in m^1^A-regulated apoptosis in liver cancer cells and that its demethylation by ALKBH3 plays a crucial role in regulating apoptotic evasion.

### m^1^A negatively regulates BIRC2 mRNA stability

**Fig. 4:**
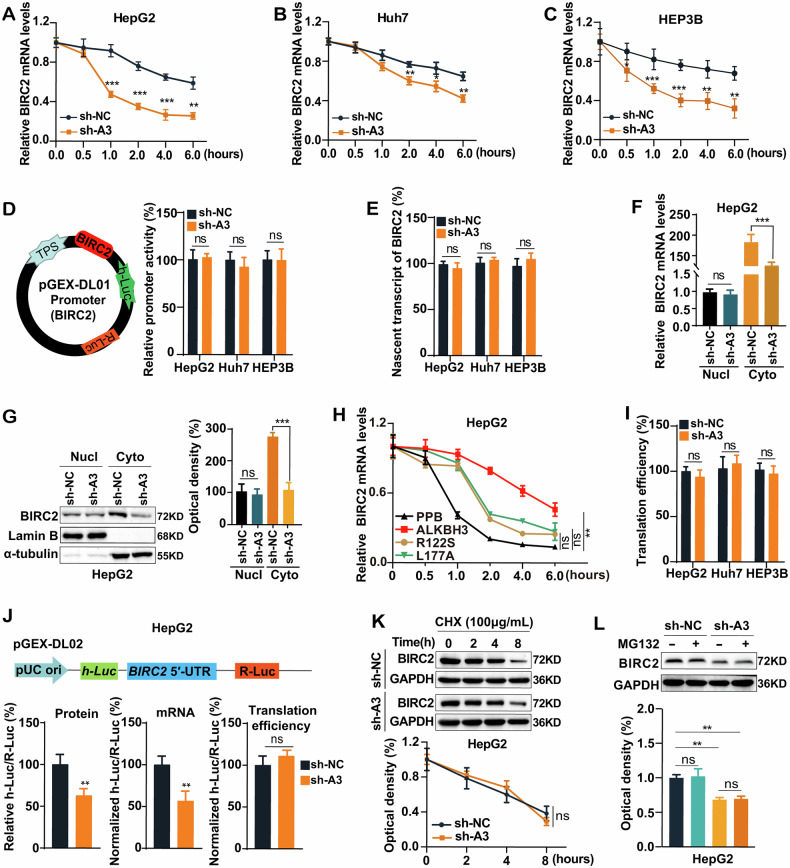
m^1^A negatively regulates BIRC2 mRNA stability. BIRC2 mRNA stability in sh-A3 and sh-NC HepG2 (**A**) Huh7 (**B**) and HEP3B (**C**) cells. Cells were treated with actinomycin D (Act-D, 1 µg/mL) for the indicated times. BIRC2 mRNA levels were measured by RT-qPCR and normalized to GAPDH. **D** BIRC2 promoter activity measured by dual-luciferase reporter assay in sh-A3 and sh-NC HepG2, Huh7, and HEP3B cells. Firefly luciferase (h-Luc) activity was normalized to Renilla luciferase (R-Luc). **E** Nascent BIRC2 transcription analyzed by nuclear run-on assay in sh-A3 and sh-NC HepG2, Huh7, and HEP3B cells. Newly synthesized BIRC2 transcripts were quantified by RT-qPCR. **F** Subcellular distribution of BIRC2 mRNA in sh-NC and sh-A3 HepG2 cells. Nuclear (Nucl) and cytoplasmic (Cyto) fractions were isolated, and BIRC2 mRNA levels were quantified by RT-qPCR and normalized to 18S (nuclear) or GAPDH (cytoplasmic). **G** Subcellular distribution of BIRC2 protein in sh-NC and sh-A3 HepG2 cells. Nuclear (Nucl) and cytoplasmic (Cyto) fractions were immunoblotted for BIRC2, Lamin B1 (nuclear), and α-Tubulin (cytoplasmic) (left). Quantification of BIRC2 protein distribution is shown (right). **H** BIRC2 mRNA stability in HepG2 cells transfected with empty vector (PPB), ALKBH3, ALKBH3-R122S, or ALKBH3-L177A for 24 h, then treated with Act-D (1 µg/mL) for the indicated times. BIRC2 mRNA levels were measured by RT-qPCR and normalized to GAPDH. **I** Translation efficiency of endogenous BIRC2, calculated as the ratio of BIRC2 protein level (by western blot) to BIRC2 mRNA level (by RT-qPCR) in sh-NC and sh-A3 HepG2 cells. **J** Translation efficiency of a BIRC2 5′-UTR-CDS reporter in sh-NC and sh-A3 HepG2 cells. Levels of h-Luc and R-Luc mRNA and protein were measured. Translation efficiency was calculated as (h-Luc/R-Luc protein) / (h-Luc/R-Luc mRNA). **K** BIRC2 protein stability in sh-NC and sh-A3 HepG2 cells treated with cycloheximide (CHX, 100 µg/mL) for the indicated times. BIRC2 protein levels were analyzed by western blot (upper), and signal intensities were quantified using ImageJ and normalized to GAPDH (lower). **L** BIRC2 protein levels in sh-NC and sh-A3 HepG2 cells treated with MG132 (2 µM) or DMSO for 6 h. BIRC2 was detected by western blot (upper), and signal intensities were quantified using ImageJ and normalized to GAPDH (lower). Data are presented as mean ± SD. Statistical analyses: **A**–**C** two-way ANOVA with Bonferroni’s test (*n* = 3); **D** unpaired *t*-test (*n* = 3); **E** unpaired *t*-test (*n* = 3); **F** unpaired *t*-test (*n* = 3); **G** unpaired *t*-test (*n* = 3); **H** two-way ANOVA with Tukey’s test (*n* = 3); **I** unpaired *t*-test (*n* = 3); **J** unpaired *t*-test (*n* = 3); **K** two-way ANOVA (*n* = 3); **L** unpaired *t*-test (*n* = 3). *p < 0.05, **p < 0.01, ***p < 0.001, ****p < 0.0001; ns, not significant.

We next investigated the mechanisms underlying m^1^A-mediated regulation of BIRC2 expression. Quantitative real-time PCR (qRT‑PCR) revealed that both the expression (Figs. 3F and S5H) and stability (Fig. 4A–C) of mature BIRC2 mRNA were significantly reduced in ALKBH3-knockdown cells. In contrast, no significant differences were observed in the expression or splicing efficiency of BIRC2 precursor mRNA (pre-mRNA) between sh-NC and sh-A3 cells (Fig. S7A–D). To further explore the regulatory mechanisms, we constructed a reporter system containing the BIRC2 promoter region (−2000 to +1 bp) upstream of the h-Luc and R-Luc genes. ALKBH3 knockdown did not significantly affect BIRC2 promoter activity (Fig. 4D). Nuclear run-on assays coupled with RT‑qPCR also showed no notable difference in nascent BIRC2 transcript levels between ALKBH3-knockdown and control cells (Fig. 4E). Subsequent fractionation of nuclear and cytoplasmic compartments showed that BIRC2 mRNA (Fig. 4F) and protein (Fig. 4G) levels were reduced in the cytoplasmic fraction of sh-A3 HepG2 cells, but not in the nuclear fraction. Notably, overexpression of wild-type ALKBH3, but not the catalytically inactive mutants R122S or L177A, significantly enhanced BIRC2 mRNA stability in HepG2 cells (Fig. 4H). These findings suggest that m^1^A negatively regulates BIRC2 mRNA stability in the cytoplasm, thereby promoting its downregulation.

We then examined the effect of m^1^A modification on BIRC2 translation and post-translational processes. ALKBH3 knockdown did not significantly alter the translation efficiency of endogenous BIRC2 mRNA (Fig. 4I). To further investigate, we generated luciferase reporters by fusing the BIRC2 5′‑UTR to the h-Luc gene in the pEGX‑DL02 plasmid. Dual-luciferase assays indicated that BIRC2 translation efficiency remained unchanged between sh-A3 and sh-NC HepG2 cells (Fig. 4J). The half-lives of BIRC2 protein were similar in sh-A3 and sh-NC HepG2 and Huh7 cells (Figs. 4K and S7E). Furthermore, pretreatment of sh-A3 and sh-NC HepG2 and Huh7 cells with the proteasome inhibitor MG132 did not prevent the reduction in BIRC2 levels induced by ALKBH3 knockdown (Figs. 4L and S7F). These results suggest that m^1^A modification does not regulate BIRC2 translation or post-translational stability, but rather specifically affects its mRNA stability.

### A98/99/100 in the 5′-UTR is the Key m^1^A methylation site regulating BIRC2 mRNA stability

**Fig. 5:**
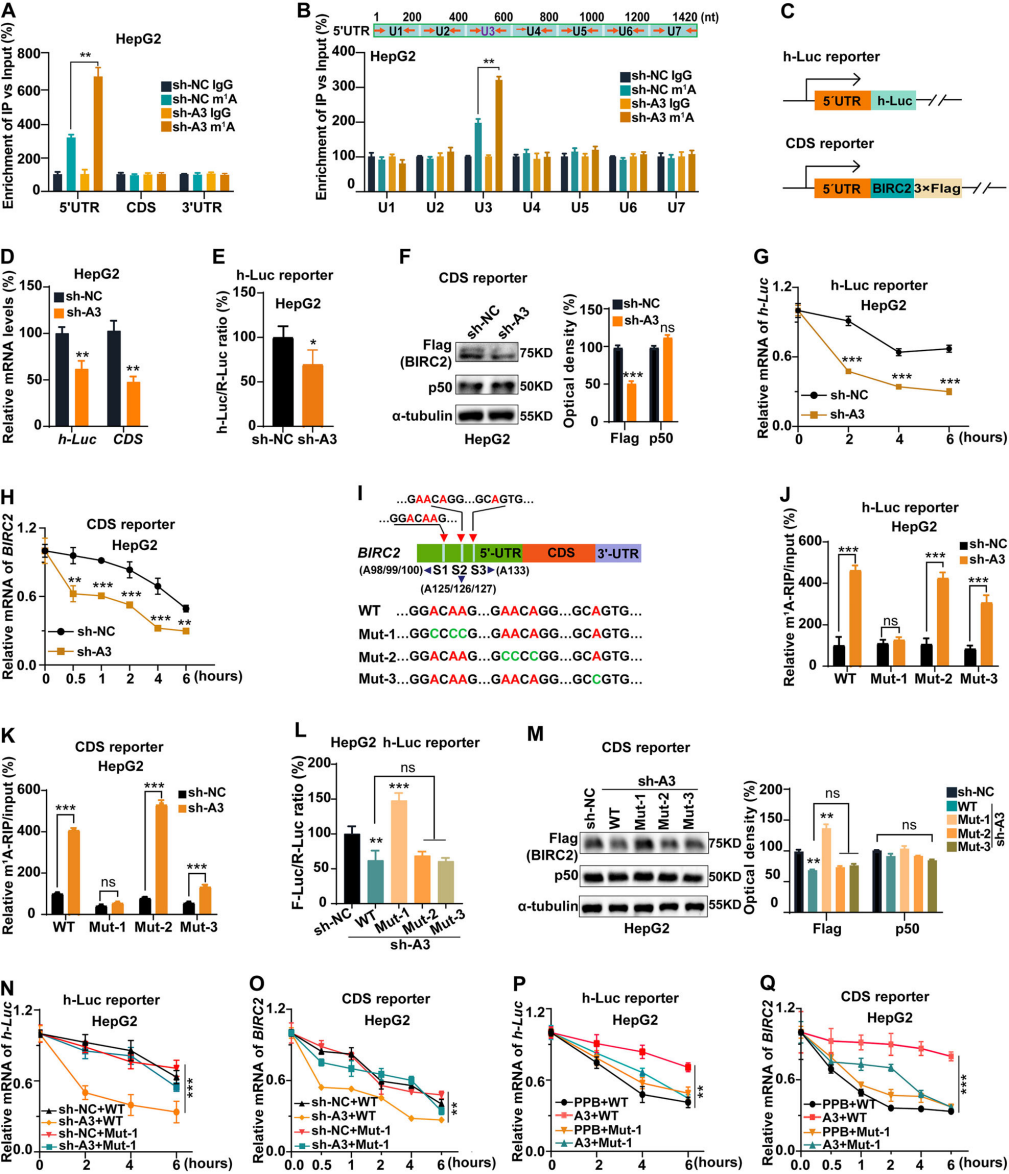
A98/99/100 in the 5′-UTR is the key m^1^A methylation site regulating BIRC2 mRNA stability. **A** Enrichment of m^1^A modification in BIRC2 mRNA in sh-NC and sh-A3 HepG2 cells was analyzed by m^1^A-RIP-qPCR using fragmented RNA from the 5′-UTR, CDS, and 3′-UTR regions. **B** Enrichment of m^1^A modification in the BIRC2 5′-UTR was analyzed by m^1^A-RIP-qPCR in sh-NC and sh-A3 HepG2 cells, assessing enrichment across seven 5′-UTR fragments (U1–U7). **C** Schematic representation of the h-Luc and CDS reporters containing the BIRC2 5′-UTR upstream of the h-Luc gene or BIRC2 5′-UTR and CDS region. **D** CDS and h-Luc reporters were transfected into sh-NC and sh-A3 HepG2 cells. Expression levels of reporter mRNAs were measured by RT-qPCR: BIRC2 mRNA for the CDS reporter (normalized to endogenous BIRC2 and GAPDH), and h-Luc mRNA for the h-Luc reporter (normalized to R-Luc mRNA). **E** h-Luc reporter was transfected into sh-NC and sh-A3 HepG2 cells for 48 h. Luciferase activity was measured and normalized to R-Luc levels. **F** CDS reporter was co-transfected with pcDNA3.1-NFKB1-HA into sh-NC and sh-A3 HepG2 cells for 48 h. Expression of exogenous BIRC2 (Flag) and p50 (NFKB1) was detected by western blot (left). Signal intensities of BIRC2 and p50 were quantified using ImageJ and normalized to α-Tubulin (right). h-Luc reporter (**G**) or CDS reporter (**H**) was transfected into sh-NC and sh-A3 HepG2 cells for 24 h, followed by Act-D (1 µg/mL) treatment for the indicated times. h-Luc mRNA or BIRC2 mRNA levels were measured by RT-qPCR. **I** Schematic of m^1^A sites within BIRC2 mRNA. Mutations in m^1^A sites (Mut-1/2/3) within the 5′-UTR are indicated. **J** sh-NC and sh-A3 HepG2 cells were transfected with h-Luc reporter-WT or Mut-1/2/3 for 24 h. Enrichment of m^1^A modification in the BIRC2 5′-UTR was analyzed by m^1^A-RIP-qPCR. **K** sh-NC and sh-A3 HepG2 cells were transfected with CDS reporter-WT or Mut-1/2/3 for 24 hours. Enrichment of m^1^A modification was analyzed by m^1^A-RIP-qPCR. **L** WT and mutated h-Luc reporters were transfected into sh-NC and sh-A3 HepG2 cells for 24 h. Luciferase activity was measured and normalized to R-Luc. **M** WT or mutated CDS reporters were co-transfected with pcDNA3.1-NFKB1-HA into sh-NC and sh-A3 HepG2 cells for 48 h. Exogenous BIRC2 (Flag) and p50 (NFKB1) expression was detected by western blot (left). Signal intensities of BIRC2 and p50 were quantified using ImageJ and normalized to α-Tubulin (right). **N** WT and Mut-1 h-Luc reporters were transfected into sh-NC and sh-A3 HepG2 cells for 24 hours, followed by Act-D (1 µg/mL) treatment for the indicated times. h-Luc mRNA levels were measured by RT-qPCR. **O** WT and Mut-1 CDS reporters were transfected into sh-NC and sh-A3 HepG2 cells for 24 h, followed by Act-D (1 µg/mL) treatment for the indicated times. BIRC2 mRNA levels were measured by RT-qPCR. **P** WT and Mut-1 h-Luc reporters were co-transfected with empty vector (PPB) or ALKBH3 constructs into HepG2 cells for 24 h, followed by Act-D (1 µg/mL) treatment for the indicated times. h-Luc mRNA levels were measured by RT-qPCR. **Q** WT and Mut-1 CDS reporters were co-transfected with empty vector (PPB) or ALKBH3 constructs into HepG2 cells for 24 h, followed by Act-D (1 µg/mL) treatment for the indicated times. BIRC2 mRNA levels were measured by RT-qPCR. Data are presented as mean ± SD. Statistical analyses: **A**, **B**, **J**, **K** unpaired *t*-test (*n* = 3); (**D**, **E**, **L**) unpaired *t*-test (*n* = 3); (**G**, **H**, **N**, **O**, **P**, **Q**) two-way ANOVA with Bonferroni’s test (*n* = 3). *p < 0.05, **p < 0.01, ***p < 0.001, ****p < 0.0001; ns, not significant.

Previous m^1^A sequencing (m^1^A-seq) [16] and independent studies [5] identified m^1^A modification peaks in the 5′-UTR of BIRC2 mRNA, with a central peak located at chr11:102,218,041 (Figs. 3E and S5G). m^1^A-RIP-qPCR assays performed on fragmented RNA confirmed significant m^1^A enrichment specifically in the 5′-UTR of BIRC2 mRNA, but not in the coding sequence (CDS) or 3′-UTR regions (Fig. 5A). To further map m^1^A distribution along the BIRC2 5′-UTR, we designed seven primer pairs (U1–U7) for targeted amplification and detected m^1^A peaks across this region. Our data indicated predominant m^1^A enrichment in the U3 fragment (Fig. 5B). Moreover, ALKBH3 overexpression significantly reduced m^1^A methylation at the U3 region of the BIRC2 5′-UTR (Fig. S8A), whereas no such effect was observed for the U2 or U4 fragments (Fig. S8B). These results suggest that m^1^A modification occurs specifically within the U3 fragment of the BIRC2 5′-UTR.

Previous studies have indicated that m^1^A or m^6^A modifications in the 5′-UTR can regulate mRNA stability [45–47]. We therefore hypothesized that m^1^A modification in the BIRC2 5′-UTR influences its mRNA stability. To test this, we constructed two reporter systems: one containing the BIRC2 5′-UTR upstream of the h-Luc gene (h-Luc reporter), and another with the BIRC2 5′-UTR and CDS upstream of h-Luc (CDS reporter) (Fig. 5C). In sh-A3 HepG2 cells, both mRNA (Fig. 5D) and protein (Fig. 5E, F) levels of the h-Luc and CDS reporters were downregulated. ALKBH3 knockdown also reduced the half-lives of both h-Luc (Fig. 5G) and BIRC2-CDS reporters (Fig. 5H), whereas overexpression of wild-type ALKBH3—but not the R122S or L177A mutants—restored reporter expression (Fig. S8C, D). These results parallel the behavior of endogenous BIRC2 mRNA, supporting the critical role of 5′-UTR m^1^A modification in regulating BIRC2 stability.

Given that m^1^A methylation is often enriched in highly structured or GC-rich regions [41], we introduced A-to-C mutations at three adenosine sites within the GC-rich segment of the U3 fragment, generating wild-type (WT) and three mutant versions (Mut-1, Mut-2, Mut-3) of both h-Luc and CDS reporters (Fig. 5I). m^1^A-RIP-qPCR analysis showed that only Mut-1 (A98/99/100) significantly reduced m^1^A enrichment levels (Fig. 5J, K), while increasing mRNA (Fig. S8E, F) and protein expression of both h-Luc (Fig. 5L) and CDS reporters (Figs. 5M and S8G). Furthermore, Mut-1 (A98/99/100) reversed the shortened half-lives of h-Luc (Fig. 5N) and BIRC2-CDS (Fig. 5O) reporters in sh-A3 HepG2 cells. Conversely, ALKBH3 overexpression in HepG2 and Huh7 cells produced opposing effects (Figs. 5P, Q and S8H–K). Together, these findings demonstrate that m^1^A modification at positions A98/99/100 in the BIRC2 5′-UTR is a key determinant of its mRNA stability.

### YTHDF3/CNOT1-XRN2 complex is Involved in m^1^A-regulated stability of BIRC2 mRNA

**Fig. 6:**
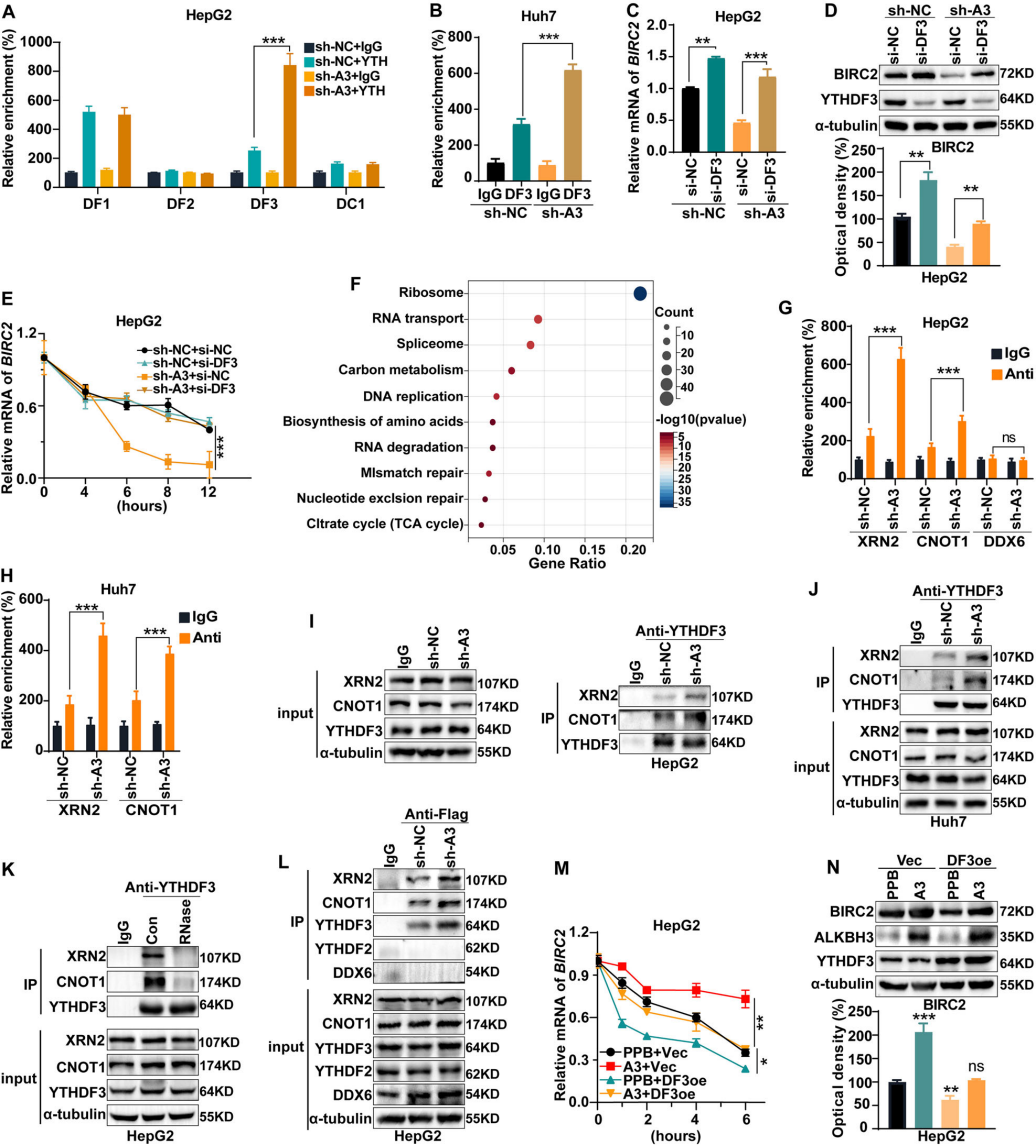
YTHDF3/CNOT1-XRN2 Complex is Involved in m^1^A-Regulated Stability of BIRC2 mRNA. **A** CLIP-qPCR analysis of BIRC2 mRNA enrichment in sh-NC and sh-A3 HepG2 cells using antibodies against YTHDF1, YTHDF2, YTHDF3, or YTHDC1. **B** CLIP-qPCR analysis of BIRC2 mRNA enrichment in sh-NC and sh-A3 Huh7 cells using an antibody against YTHDF3. **C** BIRC2 mRNA levels in sh-NC and sh-A3 HepG2 cells with YTHDF3 knockdown, measured by RT-qPCR and normalized to GADPH. **D** BIRC2 protein levels in sh-NC and sh-A3 HepG2 cells with YTHDF3 knockdown, measured by western blot (upper). Signal intensities of BIRC2 were quantified using ImageJ and normalized to α-Tubulin (lower). **E** BIRC2 mRNA stability in sh-NC and sh-A3 HepG2 cells transfected with si-NC or si-YTHDF3 for 24 h and followed by Act-D (1 µg/mL) treatment for indicated times, measured by RT-qPCR. **F** Gene Ontology (GO) enrichment analysis of proteins interacting with YTHDF3 from LC-MS/MS data. **G** CLIP-qPCR analysis of BIRC2 mRNA enrichment in sh-NC and sh-A3 HepG2 cells using antibodies against XRN2, CNOT1, or DDX6. **H** CLIP-qPCR analysis of BIRC2 mRNA enrichment in sh-NC and sh-A3 Huh7 cells using antibodies against XRN2 or CNOT1. Interaction between YTHDF3 and XRN2 or CNOT1 in sh-NC and sh-A3 HepG2 (**I**) or Huh7 (**J**) cells, assessed by co-immunoprecipitation using YTHDF3 antibody. **K** Interaction between YTHDF3 and XRN2 or CNOT1 in HepG2 cells treated with or without RNase, assessed by co-immunoprecipitation using YTHDF3 antibody. **L** Interactions between BIRC2 mRNA (CDS reporter) and YTHDF3, XRN2, CNOT1, DDX6, or YTHDF2 in sh-NC and sh-A3 HepG2 cells, analyzed by Flag antibody immunoprecipitation. **M** BIRC2 mRNA stability in HepG2 cells co-transfected with PPB or ALKBH3 (A3) and vector (Vec) or YTHDF3 (DF3oe) constructs for 24 h, followed by Act-D (1 µg/mL) treatment for indicated times, measured by RT-qPCR. **N** BIRC2 protein levels in HepG2 cells co-transfected with PPB or ALKBH3 and vector or YTHDF3 constructs for 48 hours, measured by western blot (upper). Signal intensities of BIRC2 were quantified using ImageJ and normalized to α-Tubulin (lower). Data are presented as mean ± SD. Statistical analyses: **A**, **B**, **G**, **H** unpaired *t*-test (*n* = 3); **C**, **D** one-way ANOVA with Tukey’s test (*n* = 3); **E**, **M** two-way ANOVA with Bonferroni’s test (*n* = 3); **I**–**L** representative of three independent experiments. *p < 0.05, **p < 0.01, ***p < 0.001, ****p < 0.0001; ns, not significant.

YTHDF2 and YTHDF3 are known to recognize m^1^A-modified mRNAs and regulate their stability, whereas YTHDF1 and YTHDC1 primarily influence translation or splicing [8, 13–15, 48]. CLIP-qPCR analysis revealed that YTHDF1, YTHDF3, and YTHDC1 could bind to BIRC2 mRNA, while YTHDF2 showed no detectable binding. Notably, the interaction between YTHDF3 and BIRC2 mRNA was significantly enhanced in ALKBH3-knockdown HepG2 cells (Fig. 6A). Consistent with this observation, ALKBH3-knockdown Huh7 cells also exhibited increased YTHDF3 binding to both endogenous BIRC2 mRNA (Fig. 6B) and to the h-Luc and CDS reporter constructs (Fig. S9A, B). Silencing YTHDF3 led to significant upregulation of BIRC2 mRNA (Fig. 6C) and protein (Figs. 6D and S9C) levels, as YTHDF3 depletion prevented BIRC2 mRNA degradation (Fig. 6E). Furthermore, YTHDF3 silencing did not affect the expression or stability of the Mut-1 (A98/99/100) variant of the BIRC2 CDS reporter (Fig. S9D–F), supporting the notion that YTHDF3 specifically modulates m^1^A-dependent BIRC2 mRNA stability.

Although YTHDF3 has been reported to direct methylated transcripts to YTHDF2 for accelerated degradation [48], we found that YTHDF2 did not enrich BIRC2 mRNA, and its interaction with BIRC2 mRNA remained unchanged upon YTHDF3 knockdown (Figs. 6A and S9G). Moreover, YTHDF2 silencing did not rescue the degradation of BIRC2 mRNA induced by ALKBH3 knockdown (Fig. S9H). To further elucidate the mechanism underlying YTHDF3-mediated regulation of BIRC2 mRNA stability, we performed YTHDF3 pull-down followed by mass spectrometry, identifying 333 proteins enriched in sh-A3 cells compared with sh-NC cells (Fig. S9I and Supplementary Table S4). Gene Ontology (GO) enrichment analysis revealed that these YTHDF3-interacting proteins were significantly associated with the RNA degradation pathway, including eight known regulators of mRNA stability (Fig. 6F and Supplementary Table S7). Among these, XRN2, CNOT1, and DDX6 have established roles in reducing mRNA stability and promoting mRNA degradation [49–51]. We therefore assessed the binding of BIRC2 mRNA to XRN2, CNOT1, and DDX6 using CLIP-qPCR. The results showed markedly increased binding of BIRC2 mRNA to XRN2 and CNOT1 in ALKBH3-knockdown HepG2 (Fig. 6G) and Huh7 (Fig. 6H) cells.

XRN2 (5′–3′ exoribonuclease 2) functions as the principal exonuclease responsible for 5′–3′ exonucleolytic degradation of eukaryotic mRNA. CNOT1, the core scaffold subunit of the CCR4–NOT complex, initiates mRNA degradation by mediating deadenylation and decapping, which renders transcripts accessible for subsequent exonucleolytic digestion by XRN2 and related exonucleases [52]. Co‑immunoprecipitation (co‑IP) assays further demonstrated enhanced interaction between YTHDF3 and both XRN2 and CNOT1 in ALKBH3-knockdown HepG2 and Huh7 cells (Fig. 6I, J). This interaction was RNA-dependent, as RNase treatment diminished binding (Fig. 6K). To investigate whether the identified RNA-binding proteins form a complex on the m^1^A-modified BIRC2 mRNA scaffold, we performed co-IP experiments. We used the Flag-tagged BIRC2 CDS reporter as a potential RNA scaffold and pulled down associated protein complexes with an anti-Flag antibody. This analysis confirmed that YTHDF3, XRN2, and CNOT1 were specifically enriched in the immunoprecipitates from sh-A3 HepG2 cells compared to controls, suggesting their recruitment into a complex. In contrast, neither YTHDF2 nor DDX6 was detectably co-precipitated, consistent with the CLIP-qPCR results and underscoring the specificity of the YTHDF3/CNOT1-XRN2 complex (Fig. 6L). YTHDF3 knockdown reduced the interaction between XRN2/CNOT1 and BIRC2 mRNA, whereas YTHDF2 knockdown had no such effect (Fig. S9J). In addition, YTHDF3 overexpression suppressed the upregulation of BIRC2 mRNA (Fig. S9K) and enhanced its stability (Fig. 6M), as well as increased BIRC2 protein levels (Fig. 6N) induced by ALKBH3 overexpression. These results indicate that YTHDF3, independently of YTHDF2, recruits the CNOT1–XRN2 complex to BIRC2 mRNA. CNOT1 promotes decapping and poly(A) tail shortening of BIRC2 mRNA, rendering it susceptible to 5′–3′ exonucleolytic degradation by XRN2.

### m^1^A/BIRC2 axis regulates liver cancer progression

**Fig. 7:**
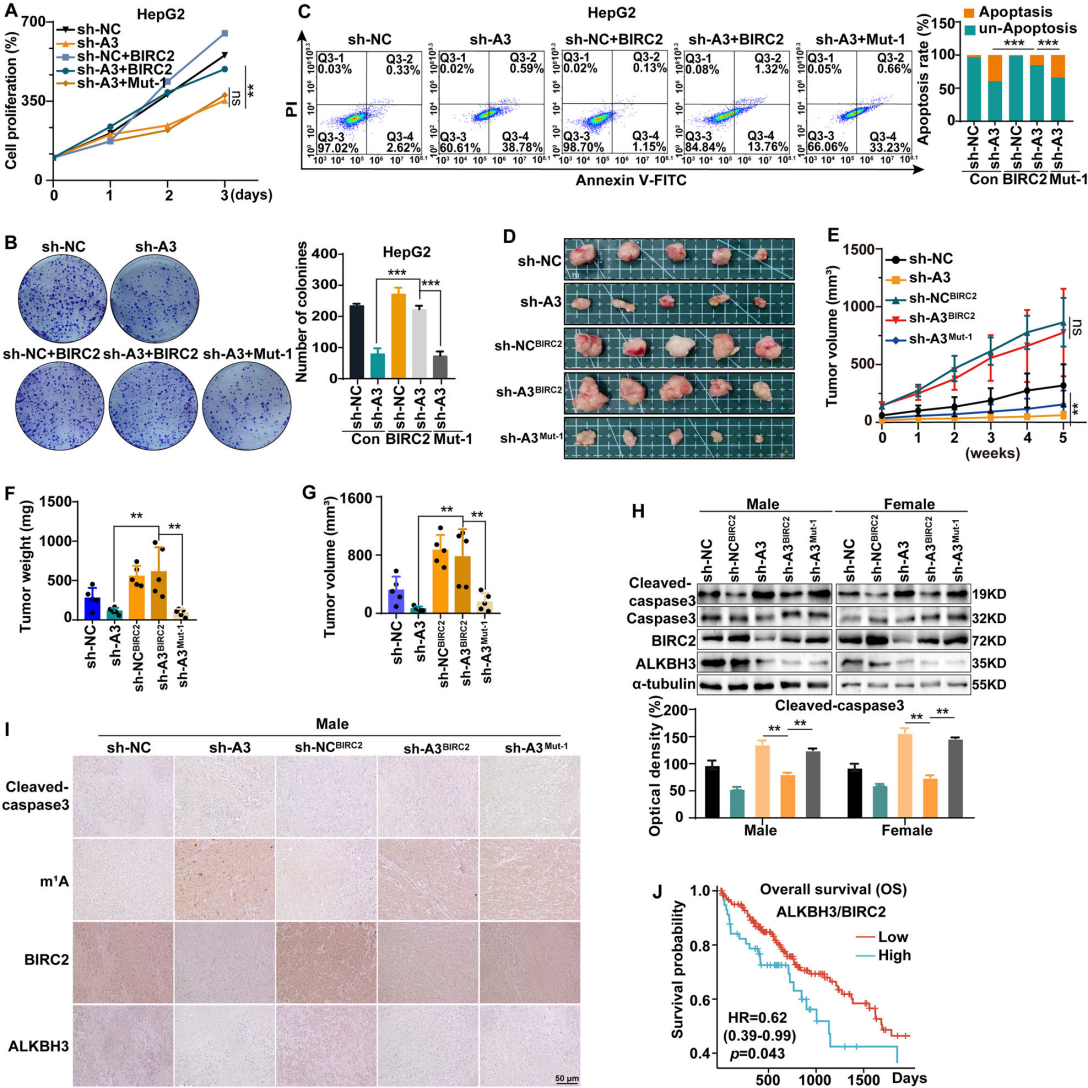
m^1^A/BIRC2 axis regulates liver cancer progression. **A** Relative cell proliferation in sh-NC and sh-A3 HepG2 cells transfected with vector control, BIRC2-WT, or BIRC2-Mut-1 constructs, measured by CCK-8 assay. **B** Colony formation ability in sh-NC and sh-A3 HepG2 cells transfected with vector control, BIRC2-WT, or BIRC2-Mut-1 constructs. Representative images (left) and quantitative analysis of colony numbers (right) are shown. **C** Apoptosis levels in sh-NC and sh-A3 HepG2 cells transfected with vector control, BIRC2-WT, or BIRC2-Mut-1 constructs, assessed by Annexin V/PI staining and flow cytometry. Representative flow cytometry plots (left) and quantitative analysis of the total apoptosis rate (right) are shown. **D** Representative images of tumor tissues from xenograft models using sh-NC and sh-A3 HepG2 cells stably transfected with vector control, BIRC2-WT, or BIRC2-Mut-1 constructs. **E** Tumor growth curves of xenograft tumors from sh-NC and sh-A3 HepG2 cells stably transfected with vector control, BIRC2-WT, or BIRC2-Mut-1 constructs. **F** Tumor weight of xenograft tumors from the indicated groups as in (**D**). **G** Tumor volume of xenograft tumors from the indicated groups as in (**D**). **H** Western blot analysis of Cleaved Caspase-3, Caspase-3, ALKBH3, and BIRC2 expression in xenograft tumor tissues (upper). Signal intensities of cleaved-caspase3 was quantified using ImageJ and normalized to α-Tubulin (lower). **I** Immunohistochemical (IHC) staining of Cleaved caspase-3, m^1^A, BIRC2, and ALKBH3 in paraffin-embedded sections from xenograft tumor tissues. Scale bar: 50 μm (×200). **J** Kaplan–Meier survival curves showing overall survival (OS) based on ALKBH3/BIRC2 expression in liver cancer patients from the TCGA-LIHC dataset. Data are presented as mean ± SD. Statistical analyses: **A**–**C** two-way ANOVA with Tukey’s test (*n* = 3); **E**–**G** two-way ANOVA with Bonferroni’s test (*n* = 5 mice per group); **J** log-rank test. *p < 0.05, **p < 0.01, ***p < 0.001, ****p < 0.0001; ns, not significant.

To investigate the role of BIRC2 in m^1^A-regulated liver cancer progression, we assessed its effects on tumor growth, clonogenicity, and apoptosis. Overexpression of BIRC2 in ALKBH3-knockdown HepG2 cells reversed the suppression of cell growth, colony formation, and apoptosis (Fig. 7A–C), and similar results were observed in ALKBH3-knockdown Huh7 cells (Fig. S10A–C). In contrast, a BIRC2 construct carrying mutations at A98/99/100 in the 5′‑UTR (Mut-1) exhibited a significantly diminished ability to rescue these phenotypes compared with wild-type BIRC2.

We then established stable HepG2 cell lines with distinct ALKBH3 and BIRC2 expression profiles—including sh-NC, sh-A3, sh-NC^BIRC2^, sh-A3^BIRC2^, and sh-A3^Mut-1^—and generated xenograft tumor models (Fig. S10D, E). Consistent with our cellular data, BIRC2 overexpression reversed the inhibitory effects of ALKBH3 knockdown on tumor growth, size, and weight. By contrast, the A98/99/100 BIRC2 mutant (sh‑A3^Mut-1^) failed to exert such restorative effects (Fig. 7D–G). Western blot (Fig. 7H) and IHC analyses (Figs. 7I and S10F) further confirmed that ALKBH3 knockdown led to elevated m^1^A levels, increased cleaved caspase‑3 expression, and reduced BIRC2 levels in xenograft tumor tissues.

We systematically evaluated the relationship among m^1^A methylation, BIRC2 expression, and liver cancer progression. Analysis of TCGA‑LIHC data revealed significant upregulation of YTHDF3 in tumor tissues relative to normal controls (Fig. S10G, *p* < 0.01), and high YTHDF3 expression was associated with poorer overall survival (Fig. S10H, *p* = 0.045). Notably, patients with concurrent overexpression of ALKBH3 and BIRC2 exhibited substantially shortened survival (Fig. 7J). Although we initially hypothesized that the YTHDF3/BIRC2 expression ratio might hold prognostic value, subsequent rigorous statistical analysis indicated that this parameter lacked independent prognostic significance in multivariate models (data not shown).

These findings underscore the importance of the “ALKBH3 (m^1^A eraser)–YTHDF3 (m^1^A reader)–BIRC2 (effector)” regulatory axis, the coordinated dysregulation of which was consistently observed in xenograft models. Through multidimensional validation—including (1) individual effector dysregulation patterns, (2) synergistic oncogenic cooperation, and (3) dynamic network remodeling—we establish that the m^1^A/BIRC2 axis promotes liver cancer malignancy via hierarchical regulatory mechanisms rather than through simple stoichiometric expression relationships.

## Discussion

Our study reveals a novel molecular mechanism through which m^1^A methylation regulates liver cancer progression by modulating BIRC2 mRNA stability. Although less characterized than other RNA modifications, m^1^A has been demonstrated to influence mRNA structural integrity and translational efficiency, thereby affecting gene expression [5, 6, 11–15]. In liver cancer, we observed that reduced m^1^A levels correlate with enhanced BIRC2 mRNA stability, promoting tumor cell survival, proliferation, and apoptosis resistance. These findings provide new insights into the functional impact of m^1^A in liver cancer pathogenesis and underscore its potential as a therapeutic target.

Evasion of apoptosis represents a hallmark of cancer and a key contributor to tumor progression [53]. Tumor cells employ diverse strategies to bypass apoptotic signals, including dysregulation of intrinsic apoptotic pathways and metabolic adaptations that inhibit programmed cell death [54]. Many cancers also overexpress anti‑apoptotic proteins such as Bcl‑2 family members to attenuate pro‑apoptotic signaling [55]. In addition, epigenetic alterations—including DNA methylation, histone modifications, and non‑coding RNA activity—contribute to the aberrant expression of apoptosis‑related genes, thereby facilitating tumor development [56]. Our work is the first to establish that m^1^A methylation plays a pivotal role in regulating apoptosis evasion in liver cancer via BIRC2. As an anti‑apoptotic protein, BIRC2 upregulation enhances tumor cell resistance to apoptotic stimuli. This was further corroborated by immunohistochemical analysis showing that ALKBH3 knockdown led to decreased BIRC2 expression in tumor tissues. Such disruption of the survival–death balance in the tumor microenvironment likely contributes to sustained tumor cell viability in liver cancer.

Notably, the role of m^1^A modification in regulating mRNA stability, particularly within the 5′‑UTR, remains poorly understood. Recent studies have linked m^1^A methylation to mRNA decay, as exemplified by ALKBH3‑mediated demethylation promoting tumor progression through SP100A mRNA degradation [47]. In addition, m^6^A modifications in the 5′‑UTR have been shown to modulate mRNA stability in other systems [45, 46]. Our study extends this knowledge by demonstrating that m^1^A critically regulates BIRC2 mRNA stability through the following evidence: (1) Using m^1^A‑seq and m^1^A‑RIP‑qPCR, we identified the 5′‑UTR of BIRC2 mRNA as a hotspot for m^1^A modification, with residues A98/99/100 being essential for maintaining mRNA stability; (2) Mut-1 (A98/99/100) mutation led to elevated expression and extended half‑life of BIRC2 mRNA, indicating that m^1^A methylation in this region plays a central role in BIRC2 mRNA turnover; and (3) We verified that BIRC2 is specifically regulated by m^1^A and is not a target of other common RNA modifications such as m^6^A or m^5^C [57–59], highlighting the selectivity of m^1^A in this regulatory process.

**Fig. 8:**
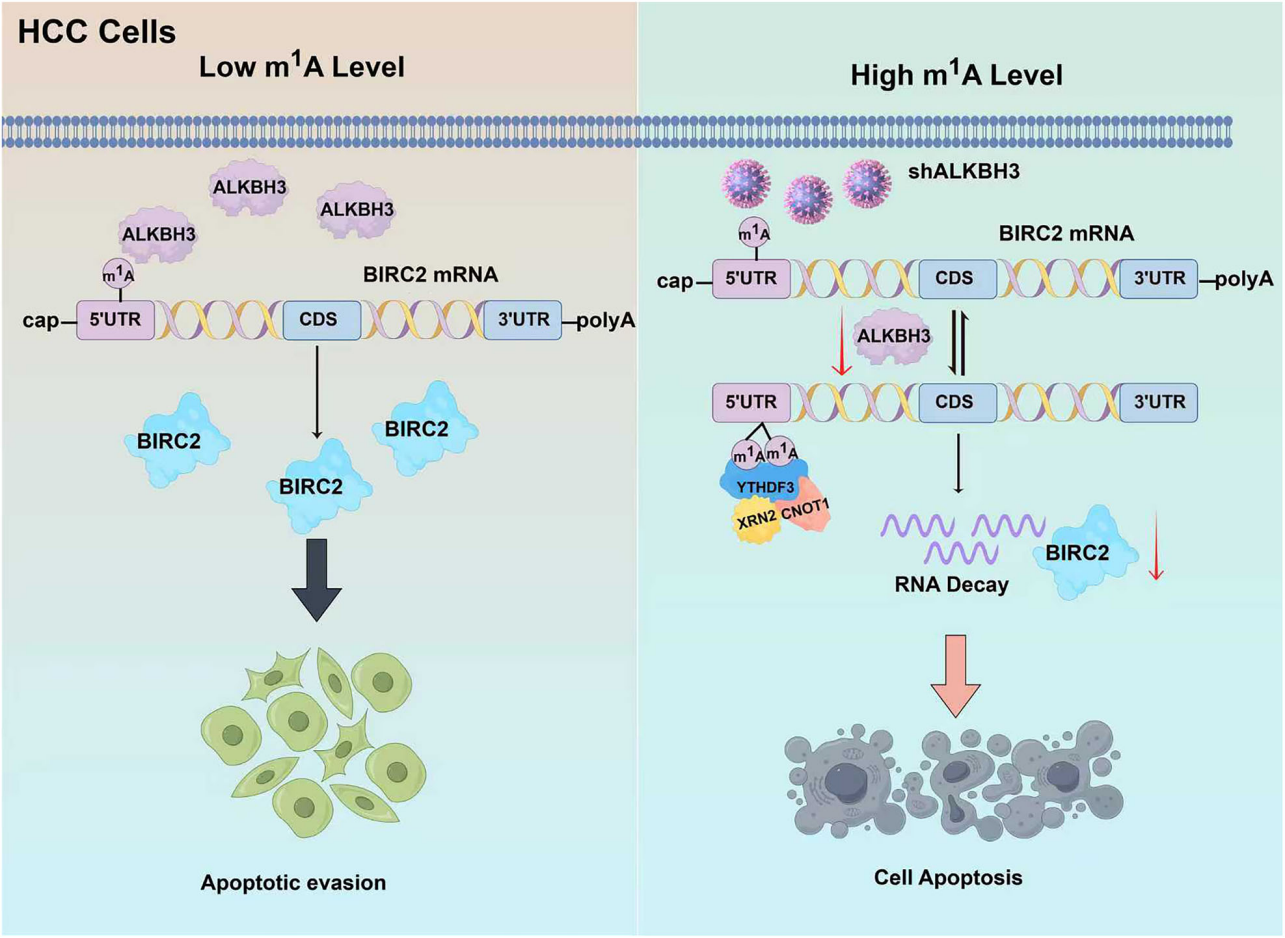
Proposed Model to illustrate the Mechanisms of m^1^A-Regulated Stability of BIRC2 in Liver Cancer Cells (by Figdraw).

Our study also elucidates key molecular effectors involved in m^1^A‑dependent regulation of BIRC2 mRNA stability. We found that the m^1^A reader YTHDF3—previously implicated in m^6^A‑mediated mRNA decay in the cytoplasm [48, 60, 61]—binds m^1^A‑modified BIRC2 mRNA independently of YTHDF2. This interaction recruits the RNA degradation machinery, including CNOT1 and XRN2, to destabilize BIRC2 mRNA (Fig. 8). YTHDF3, CNOT1, and XRN2 form a functional complex that promotes the decay of m^1^A‑methylated transcripts, revealing a previously unrecognized mechanism of mRNA destabilization. Co‑immunoprecipitation assays further confirmed that YTHDF3 interacts with CNOT1 and XRN2 to facilitate BIRC2 mRNA degradation in a YTHDF2‑independent manner. These findings broaden the current model of m^1^A‑driven mRNA regulation and offer new perspectives on how RNA modifications contribute to mRNA stability and gene expression.

In summary, this study provides new insights into the role of m^1^A methylation in regulating BIRC2 mRNA stability and its impact on liver cancer progression. By delineating the mechanism through which m^1^A influences apoptosis evasion and tumor survival, our work lays a foundation for developing targeted therapeutic strategies aimed at modulating RNA modifications. Intervention in the m^1^A pathway may offer a promising new direction for improving liver cancer treatment and patient outcomes.

## Supplementary information

Supplementary File

Supplementary Tables

Supplemental Material

## Data Availability

All data generated or analysed during this study are included in this published article and its supplementary information files (containing detailed procedures of methods, ten figures, and seven tables). The mass spectrometry proteomics data generated in this study have been deposited in the National Genomics Data Center (https://ngdc.cncb.ac.cn/) via the OMIX database under accession number OMIX01275 [36]. The public liver hepatocellular carcinoma (LIHC) data supporting the findings of this study are available from The Cancer Genome Atlas (TCGA) portal (https://portal.gdc.cancer.gov/) and are referenced in accordance with TCGA publication guidelines [35]. Additional bioinformatics analyses were performed using LinkedOmics (http://www.linkedomics.org/login.php) [38] and the Kaplan–Meier plotter tool (http://kmplot.com/analysis/) [39].
