# Supplementary material for: m1A-mediated regulation of BIRC2 mRNA stability drives apoptosis evasion and tumor progression in liver cancer
Source: Cell Death Dis. 2026 Apr 16;17(1):630. doi: 10.1038/s41419-026-08731-z (PMC13350948; doi:10.1038/s41419-026-08731-z)
Supplement: Supplementary file 1 — Supplementary File [file 41419_2026_8731_MOESM1_ESM.pdf]

Supplementary data for

m^{1}A-Mediated Regulation of BIRC2 mRNA Stability Drives Apoptosis Evasion and Tumor Progression in Liver Cancer

Yingmin Wu[1,2,5,6#], Shenjie Zhang[1#], Shilong Zhang[1,4#], Jieyu Lu [2,5], Yuntao Yang[2,5], Zhirui Zeng[1,2,5], Shan Lei[2,5], Rui Mi[1], Yewei Zhang[1], Lichen Ge[3]*, Tengxiang Chen[1,2,5,6]*, Haiyang Li[1,6]*

[1] *Department of Surgery, Affiliated Hospital of Guizhou Medical University, 550004 Guiyang, China.*

[2] *Department of Physiology and Pathophysiology, School of Basic Medical Sciences, Guizhou Medical University, 561113 Guiyang, China.*

[3] *Department of Laboratory Medicine, Third Affiliated Hospital of Sun Yat-sen University, Guangzhou 510630, China.*

[4] *Department of Breast Thyroid Surgery, The Third People's Hospital of Chengdu, The Affiliated Hospital of Southwest Jiao tong University, 610014 Chengdu, China.*

[5] *Transformation Engineering Research Center of Chronic Disease Diagnosis and Treatment, Guizhou Medical University, 561113 Guiyang, China.*

[6] *Guizhou Institute of Precision Medicine, Affiliated Hospital of Guizhou Medical University, 550004 Guiyang, China.*

[#] These authors contributed equally to this work.

*** Co-corresponding authors:** To whom correspondence should be addressed. Prof. Haiyang Li. Email: lihaiyang@gmc.edu.cn; Correspondence may also be addressed to Prof

Tengxiang Chen. Email: txch@gmc.edu.cn; Correspondence may also be addressed to PhD Lichen Ge. Email: gelch5@mail.sysu.edu.cn.

Figure S1

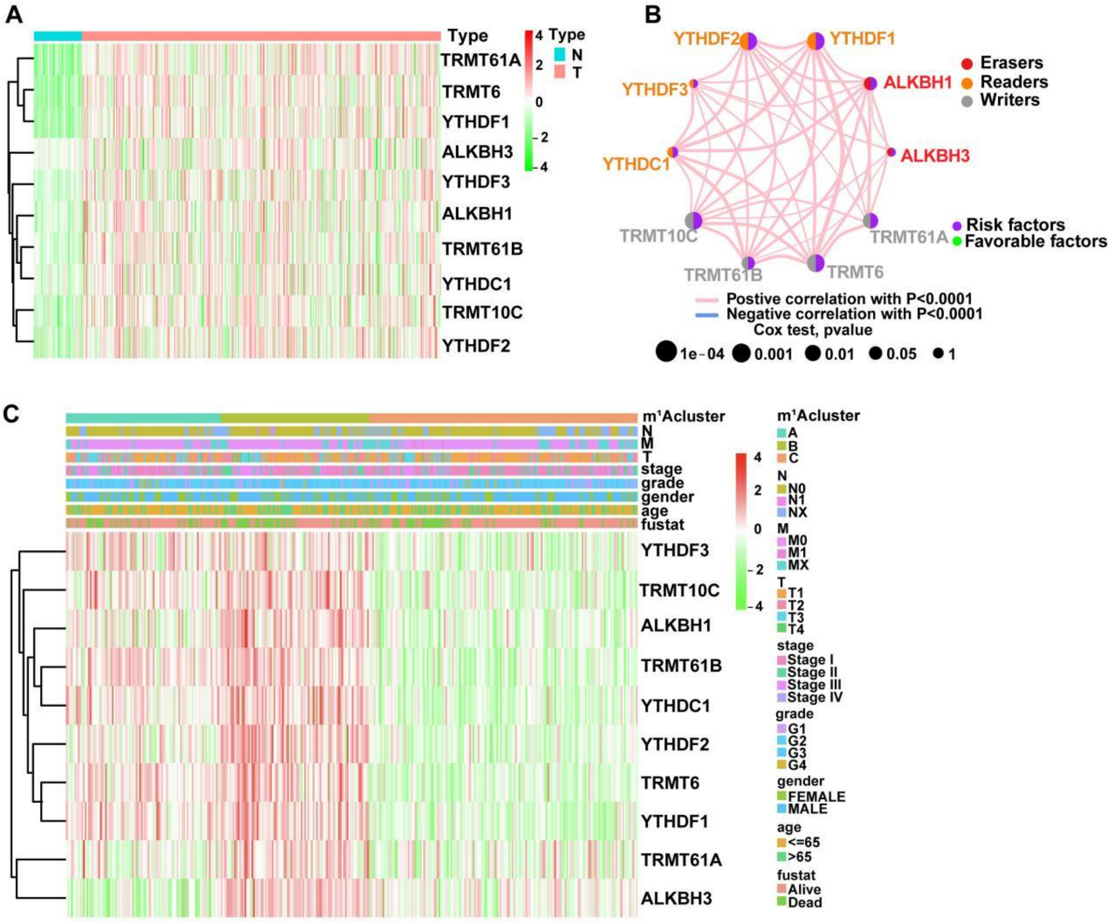

Figure S1 Reduced m^{1}A Levels in Liver Cancer and Association with Disease Progression

Related to Figure 1

(A) Comparative analysis of the expression of 10 m^{1}A regulators in liver tumor and adjacent normal tissues from the TCGA-LIHC dataset.

(B) Interaction network of 10 m^{1}A regulators in liver cancer. Circle size represents prognostic significance (log-rank test *p*-values). Green nodes: risk factors; purple nodes: protective factors. Line thickness indicates correlation strength (blue: negative; pink: positive). Regulator clusters: Erasers (red), Readers (orange), Writers (gray).

(C) Relationships between clinicopathological features and m¹A-regulated gene subgroups in liver cancer. PCA: principal component analysis; K-M: Kaplan-Meier.

Statistical note: In panels (A) and (C), significance is indicated as *p < 0.05, **p < 0.01. Network correlations in (B) were assessed by Spearman's rank correlation test.

Figure S2

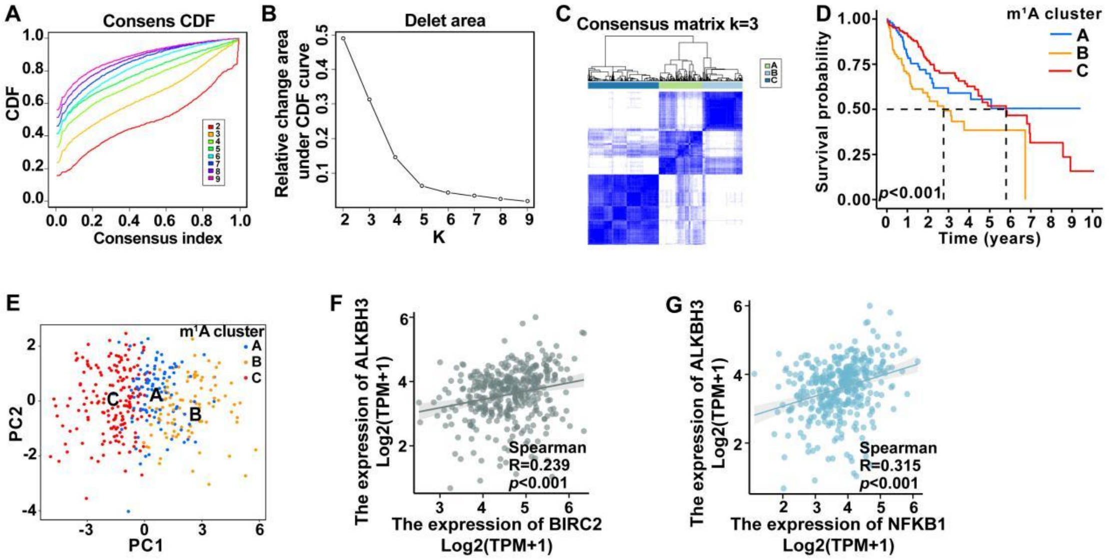

Figure S2 Consensus Clustering of m¹A Regulators Defines Clinically Distinct Liver Cancer Subtypes

Related to Figure 2

(A–C) Consensus clustering analysis of 10 m¹A regulators in the TCGA-LIHC cohort.

(D) Kaplan–Meier survival curves showing overall survival (OS) of liver cancer patients in Clusters A, B, and C.

(E) Principal component analysis (PCA) of expression profiles of 10 m¹A regulators, categorizing liver cancer patients into three distinct subgroups (A: blue, B: yellow, C: red).

(F) Correlation between ALKBH3 and BIRC2 mRNA expression in TCGA-LIHC samples.

(G) Correlation between ALKBH3 and NFKB1 mRNA expression in TCGA-LIHC samples.

Data are presented as mean ± SD where applicable. Statistical analyses: (D) log-rank test; (F, G) Spearman's correlation. *p < 0.05, **p < 0.01, ***p < 0.001.

Figure S3

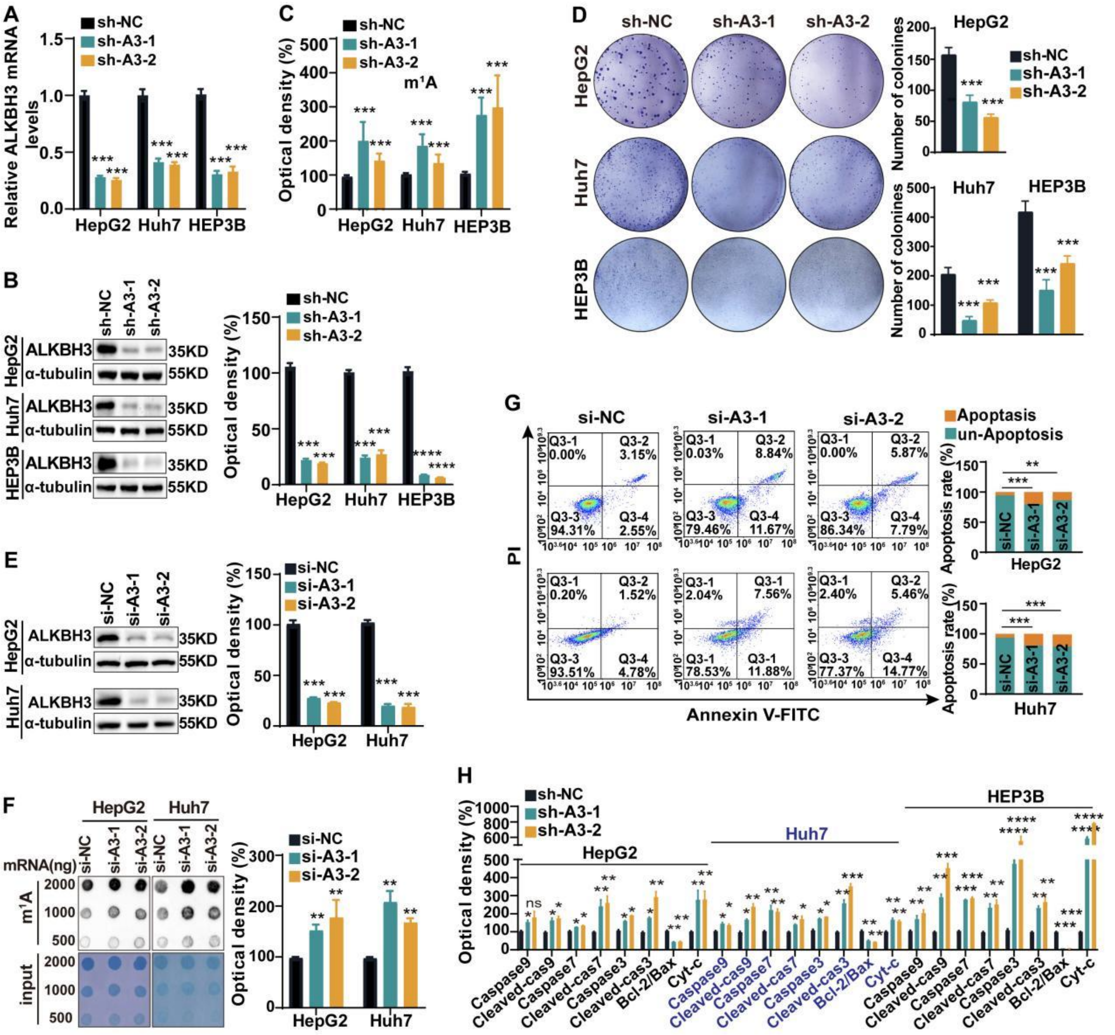

Figure S3 m^{1}A Modification Promotes Apoptosis and Suppresses Proliferation in Liver Cancer Cells

Related to Figure 2

(A) Relative ALKBH3 mRNA expression in sh-A3 and sh-NC HepG2, Huh7, and HEP3B cells, measured by RT-qPCR and normalized to GAPDH.

(B) ALKBH3 protein levels in sh-A3 and sh-NC HepG2, Huh7, and HEP3B cells were determined by western blot. The representative images (left) and quantification (right, normalized to α-Tubulin) are presented.

(C) Quantification of m^{1}A levels from dot-blot assays in Figure 2D. Signal intensities were quantified using ImageJ and normalized to total mRNA stained with methylene blue.

(D) Colony formation assay in sh-A3 and sh-NC HepG2, Huh7, and HEP3B cells. Representative images (left) and quantitative analysis of colony numbers (right) are shown.

(E) ALKBH3 protein levels were determined by western blot in HepG2 and Huh7 cells after transfection with si-NC, si-ALKBH3-1, or si-ALKBH3-2 for 48 hours. The representative images (left) and quantification (right, normalized to α-Tubulin) are presented .

(F) mRNA m^1A levels were determined by dot-blot in HepG2 and Huh7 cells after transfection with si-NC, si-ALKBH3-1, or si-ALKBH3-2 for 48 hours. Representative blots (left) and quantitative analysis (right, normalized to total mRNA stained with methylene blue) are shown.

(G) Apoptosis assessment in HepG2 and Huh7 cells transfected with si-NC, si-ALKBH3-1, or si-ALKBH3-2 for 48 hours by Annexin V-FITC/PI staining and flow cytometry. Representative flow cytometry plots (left) and quantitative analysis of the total apoptosis rate (right) are shown.

(H) Quantification of apoptosis-related proteins levels from western blot assays in Figure 2J. Signal intensities were quantified using ImageJ and normalized to α-Tubulin.

Data are presented as mean ± SD. Statistical analyses: (A, B, C, E, F, G, H) one-way ANOVA with Tukey's test (n = 3); (D) unpaired t-test (n = 3). $*p < 0.05$, $**p < 0.01$, $***p < 0.001$; ns, not significant.

Figure S4

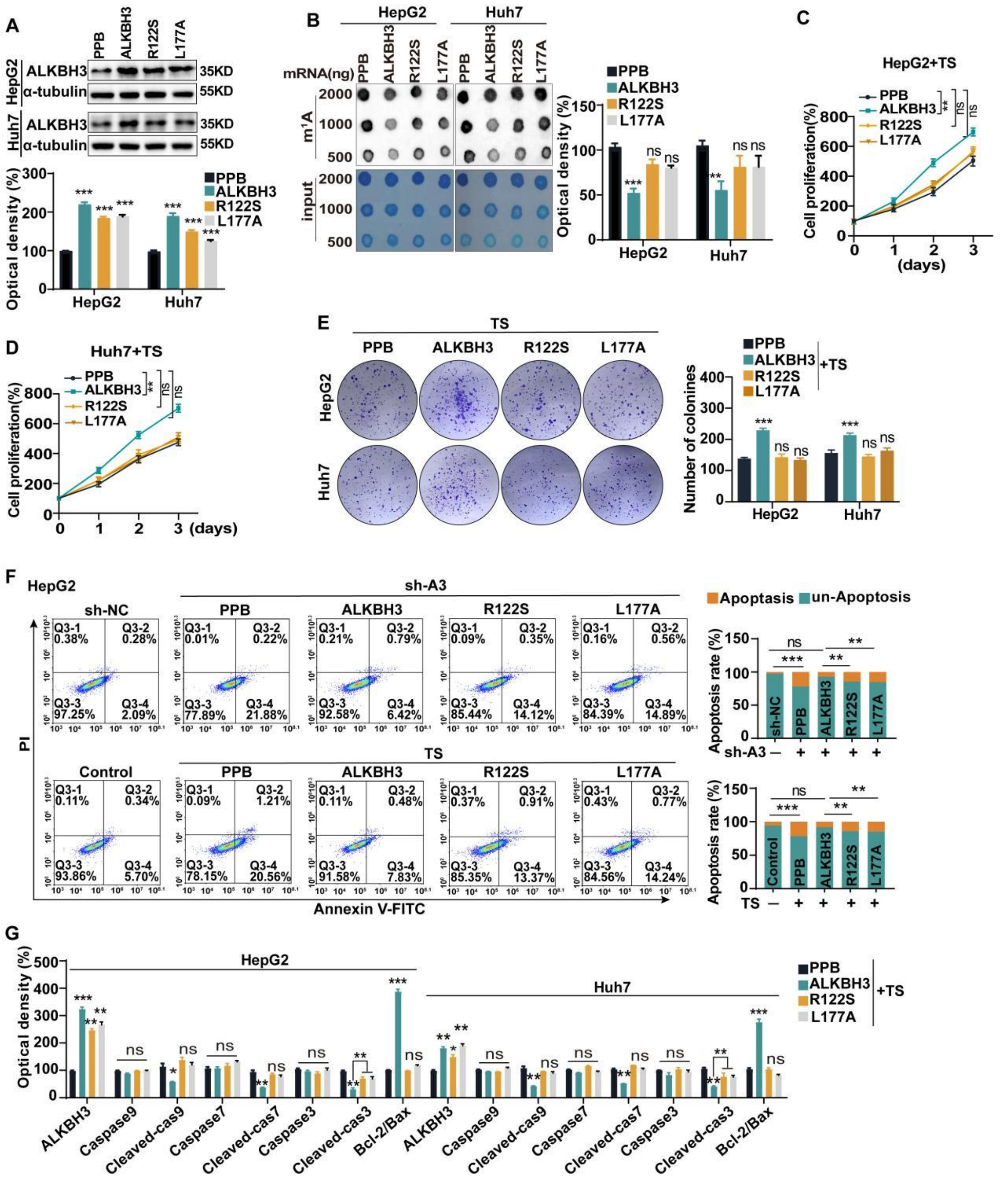

Figure S4 ALKBH3 Mutants Modulate m^{1}A-Driven Cell Proliferation, Colony Formation, and Apoptosis in Liver Cancer

Related to Figure 2

(A) ALKBH3 protein expression in HepG2 and Huh7 cells transfected with empty vector (PPB), ALKBH3, ALKBH3-R122S, or ALKBH3-L177A for 48 hours were analyzed by western blot. The representative images (upper) and quantification (lower, normalized to α-Tubulin) are presented.

(B) m^1A mRNA levels in HepG2 and Huh7 cells transfected as in (A) were measured by dot-blot assay (left). Signal intensities were quantified using ImageJ and normalized to total mRNA stained with methylene blue (right).

(C) HepG2 cells were transfected with the indicated constructs for 48 hours, followed by treatment with TNF-α + SM-164 (TS) for 6 hours. Cell proliferation was assessed using the CCK-8 assay at indicated time points.

(D) Proliferation of Huh7 cells treated as in (C).

(E) Colony formation ability of HepG2 and Huh7 cells treated as in (C).

(F) Apoptosis levels assessed by flow cytometry: (upper) sh-A3 HepG2 cells transfected with the indicated constructs for 48 hours; (lower) HepG2 cells were transfected with the indicated constructs for 48 hours, followed by treatment with TS for 6 hours. Representative flow cytometry plots (left) and quantitative analysis of the total apoptosis rate (right) are shown.

(G) Quantification of apoptosis-related proteins levels from western blot assays in Figure 2L. Signal intensities were quantified using ImageJ and normalized to α-Tubulin.

Data are presented as mean ± SD. Statistical analyses: (A, B, F, G) one-way ANOVA with Tukey's test (n = 3); (C, D) two-way ANOVA with Bonferroni's test (n = 3); (D) unpaired t-test (n = 3). *$p < 0.05$, **$p < 0.01$, ***$p < 0.001$; ns, not significant.

Figure S5

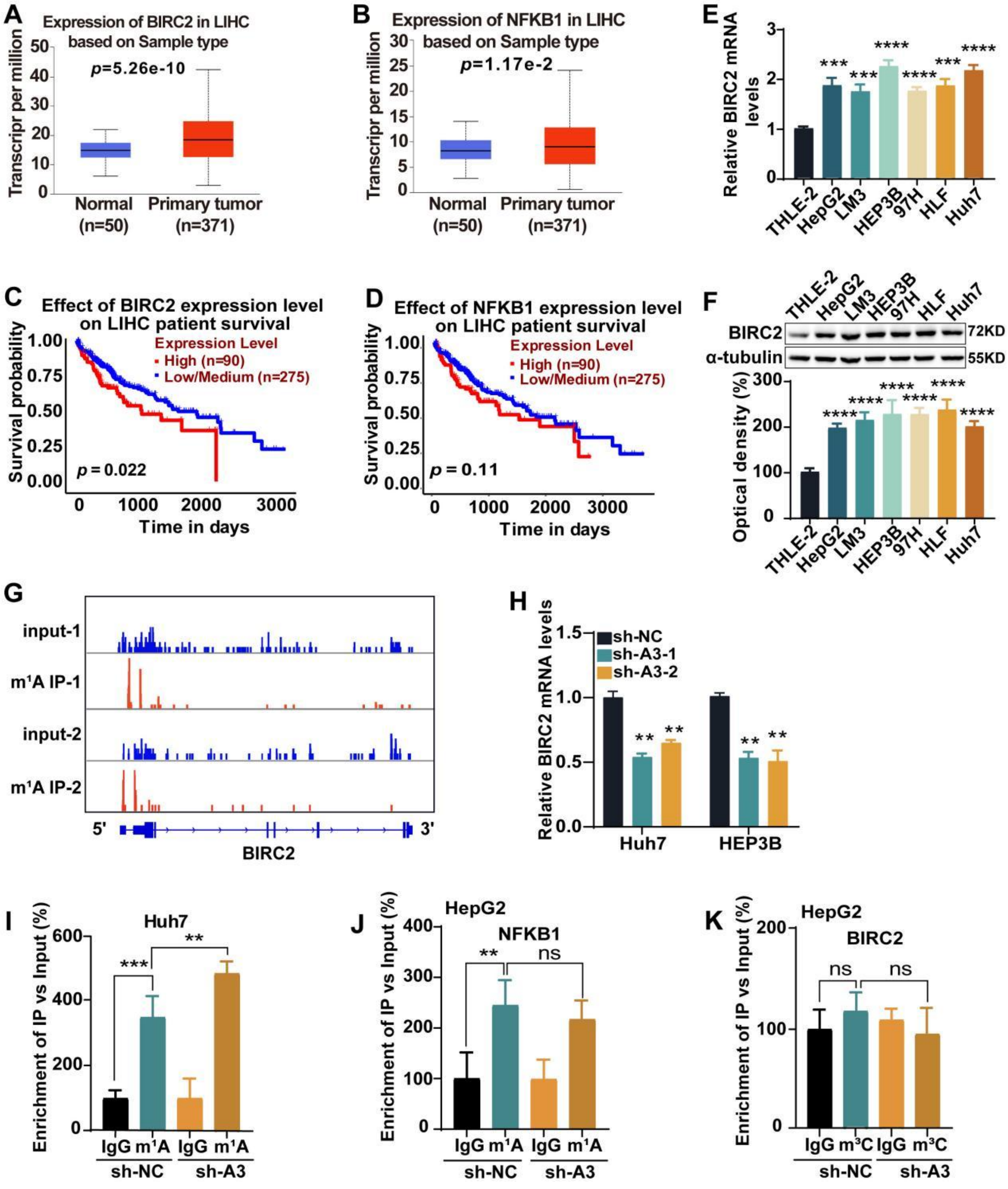

Figure S5 BIRC2 is an m^1A-Modified Transcript

Related to Figure 3

(A) BIRC2 mRNA expression in liver tumor and normal tissues from the TCGA-LIHC database.

(B) NFKB1 mRNA expression in liver tumor and normal tissues from the TCGA-LIHC database.

(C) Kaplan–Meier survival curves of overall survival (OS) based on BIRC2 expression in TCGA-LIHC patients.

(D) Kaplan–Meier survival curves of overall survival (OS) based on NFKB1 expression in TCGA-LIHC patients.

(E) BIRC2 mRNA levels in multiple liver cancer cell lines and the normal hepatocyte line THLE-2, measured by RT-qPCR and normalized to GAPDH.

(F) BIRC2 protein levels in liver cancer cell lines and THLE-2 cells, analyzed by western blot (upper). Signal intensities of BIRC2 was quantified using ImageJ and normalized to α-Tubulin (lower).

(G) m^1A RIP-seq profiles showing m^1A peak enrichment in the 5′-UTR of BIRC2 mRNA in HepG2 cells [1].

(H) Relative BIRC2 mRNA expression in sh-A3 and sh-NC Huh7 and HEP3B cells, measured by RT-qPCR and normalized to GAPDH.

(I) m^1A RIP-qPCR analysis of BIRC2 mRNA enrichment in sh-A3 versus sh-NC Huh7 cells.

(J) m^1A RIP-qPCR analysis of NFKB1 mRNA enrichment in sh-A3 versus sh-NC HepG2 cells.

(K) m^3C RIP-qPCR analysis of BIRC2 mRNA enrichment in sh-A3 versus sh-NC HepG2 cells.

Data are presented as mean ± SD. Statistical analyses: (A, B) unpaired t-test; (C, D) log-rank test; (E, H–K) unpaired t-test (n = 3); (I–K) representative of three independent experiments. *$p < 0.05$, **$p < 0.01$, ***$p < 0.001$; ns, not significant.

Figure S6

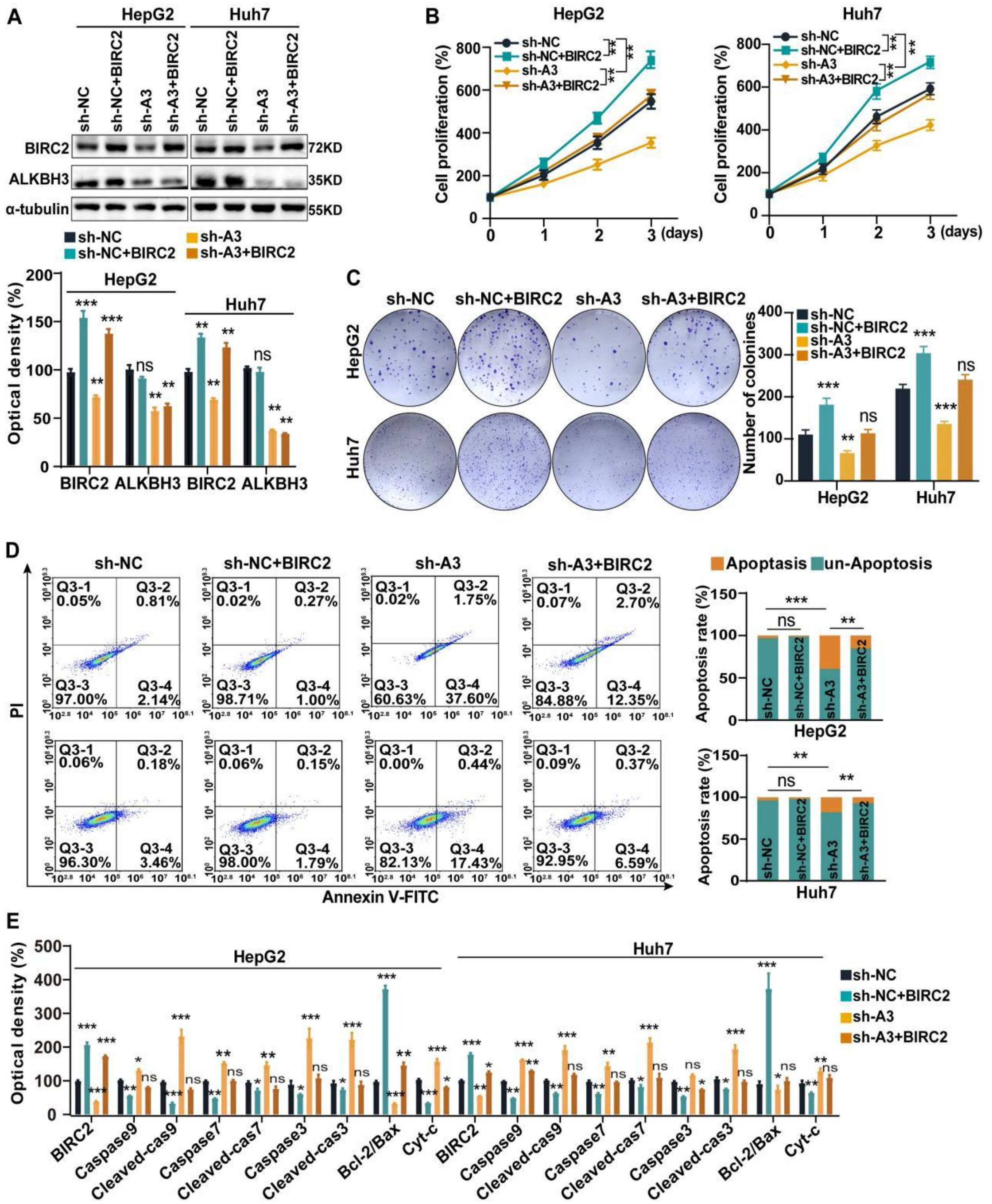

Figure S6 BIRC2 Involvement in m^{1}A-Regulated Apoptosis in Liver Cancer Cells Related to Figure 3

(A) Western blot analysis of BIRC2 and ALKBH3 expression in sh-A3 and sh-NC HepG2 and Huh7 cells transfected with empty vector (pcDNA3.1) or BIRC2 constructs for 48 hours (upper). Signal intensities of BIRC2 and ALKBH3 were quantified using ImageJ and normalized to α-Tubulin (lower).

(B) Proliferation of sh-A3 and sh-NC HepG2 (left) and Huh7 (right) cells transfected with empty vector or BIRC2 constructs, measured by CCK-8 assay at indicated time points.

(C) Colony formation ability of sh-A3 and sh-NC HepG2 (upper) and Huh7 (lower) cells transfected with empty vector or BIRC2 constructs, with quantitative analysis shown in the right.

(D) Apoptosis rates in sh-A3 and sh-NC HepG2 (upper) and Huh7 (lower) cells transfected with empty vector or BIRC2 constructs, assessed by Annexin V/PI staining and flow cytometry. Quantitative analysis of the total apoptosis rate are shown in the right.

(E) Quantification of apoptosis-related proteins levels from western blot assays in Figure 3J. Signal intensities were quantified using ImageJ and normalized to α-Tubulin.

Data are presented as mean ± SD. Statistical analyses: (B) two-way ANOVA with Bonferroni's test (n = 3); (C, D, E) one-way ANOVA with Tukey's test (n = 3). **p* < 0.05, ***p* < 0.01, ****p* < 0.001; ns, not significant.

Figure S7

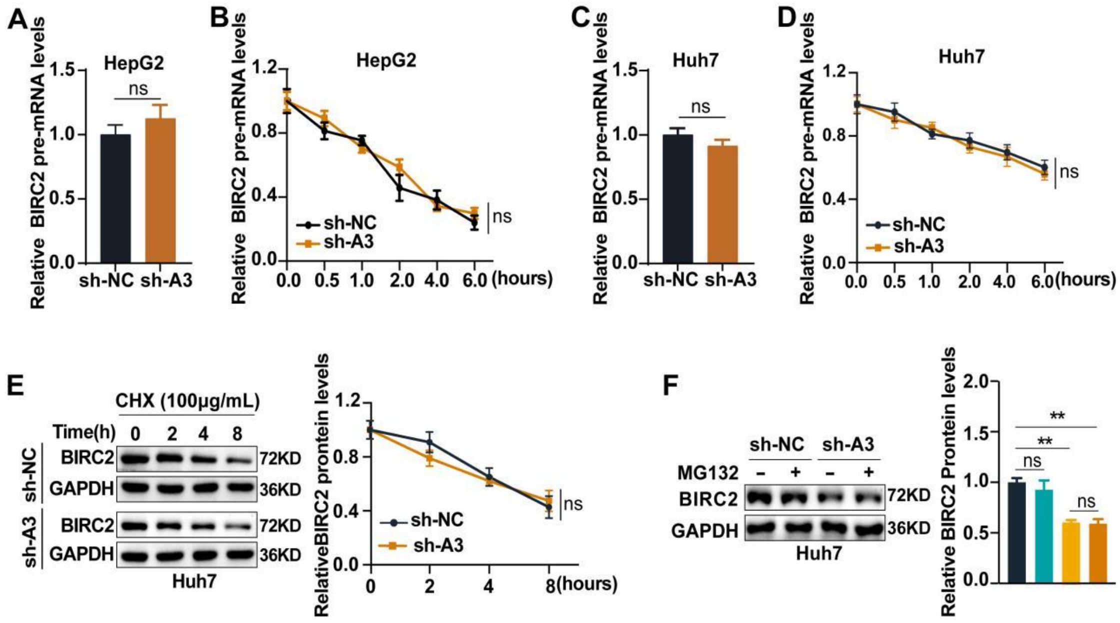

Figure S7 m[1]A Negatively Regulates the Stability of BIRC2 mRNA

Related to Figure 4

(A and C) Expression of BIRC2 pre-mRNA in sh-NC and sh-A3 HepG2 (A) and Huh7 (C) cells, measured by RT-qPCR and normalized to 18S.

(B and D) sh-NC and sh-A3 HepG2 (B) and Huh7 (D) cells were treated with actinomycin D (Act-D, 1 μg/mL) for the indicated times. Splicing efficiency of BIRC2 pre-mRNA were measured by RT-qPCR and normalized to 18S.

(E) BIRC2 protein stability in sh-NC and sh-A3 Huh7 cells treated with cycloheximide (CHX, 100 μg/mL) for indicated times, analyzed by western blot (left) and signal intensities were quantified using ImageJ and normalized to GAPDH (right).

(F) BIRC2 protein levels in sh-NC and sh-A3 Huh7 cells treated with MG132 (2 μM) or DMSO for 6 hours, analyzed by western blot (left) and signal intensities were quantified using ImageJ and normalized to GAPDH (right).

Data are presented as mean ± SD. Statistical analyses: (A–D) unpaired t-test (n = 3); (E) two-way ANOVA with Bonferroni's test (n = 3); (F) unpaired t-test (n = 3). *$p < 0.05$, **$p < 0.01$, ***$p < 0.001$; ns, not significant.

Figure S8

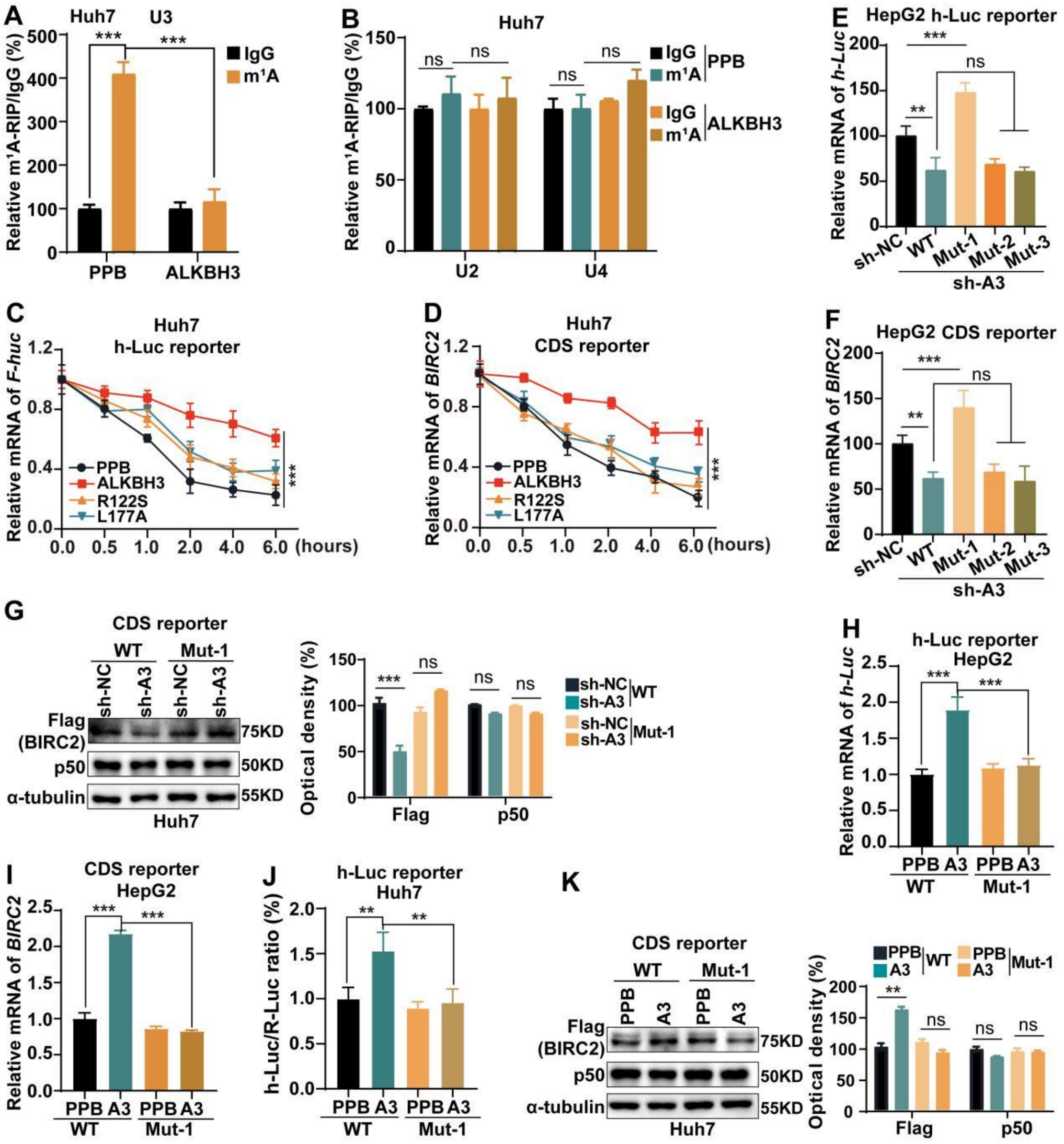

Figure S8 A98/99/100 in the 5′-UTR is the Key m^{1}A Methylation Site Regulating BIRC2 mRNA Stability

Related to Figure 5

(A) m^{1}A-RIP-qPCR analysis of m^{1}A enrichment in the U3 region of BIRC2 5′-UTR in Huh7 cells transfected with empty vector or ALKBH3 for 24 hours, using fragmented RNA.

(B) m^{1}A-RIP-qPCR analysis of m^{1}A enrichment in U2 or U4 regions of BIRC2 5′-UTR in Huh7 cells transfected as in (A).

(C) h-Luc reporter mRNA stability in Huh7 cells transfected with empty vector (PPB), ALKBH3, ALKBH3-R122S, or ALKBH3-L177A for 24 hours, followed by Act-D (1 μg/mL)

treatment for the indicated times, measured by RT-qPCR and normalized to R-Luc.

(D) CDS reporter mRNA stability in Huh7 cells transfected and treated as in (C), measured by RT-qPCR and normalized to endogenous BIRC2 and GAPDH mRNA.

(E) h-Luc mRNA levels of WT or mutated h-Luc reporters in sh-NC and sh-A3 HepG2 cells were measured by RT-qPCR , normalized to R-Luc mRNA.

(F) BIRC2 CDS mRNA levels of WT or mutated CDS reporters in sh-NC and sh-A3 HepG2 cells were measured by RT-qPCR, normalized to endogenous BIRC2 and GAPDH mRNA.

(G) Western blot analysis of exogenous Flag-BIRC2 and p50 in sh-NC and sh-A3 Huh7 cells co-transfected with WT or Mut-1 CDS reporters and pcDNA3.1-NFKB1-HA for 48 hours (left). Band intensities were quantified using ImageJ, and normalized to α-Tubulin (right).

(H) h-Luc mRNA levels in HepG2 cells co-transfected with WT or Mut-1 h-Luc reporters and empty vector or ALKBH3 for 24 hours, measured by RT-qPCR and normalized to R-Luc.

(I) BIRC2 mRNA levels in HepG2 cells co-transfected with WT or Mut-1 CDS reporters and empty vector or ALKBH3 for 24 hours, measured by RT-qPCR and normalized to endogenous BIRC2 and GAPDH mRNA.

(J) Luciferase activity in Huh7 cells co-transfected with WT or Mut-1 h-Luc reporters and empty vector or ALKBH3 for 48 hours, normalized to R-Luc.

(K) Western blot analysis of exogenous Flag-BIRC2 expression in Huh7 cells co-transfected with WT or Mut-1 CDS reporters and empty vector or ALKBH3 for 48 hours (1eft). Band intensities were quantified using ImageJ, and normalized to α-Tubulin (right).

Data are presented as mean ± SD. Statistical analyses: (A, B, E, F, H, I, J) unpaired t-test (n = 3); (C, D) two-way ANOVA with Bonferroni's test (n = 3); (G, K) one-way ANOVA with Tukey's test (n = 3). *$p < 0.05$, **$p < 0.01$, ***$p < 0.001$; ns, not significant.

Figure S9

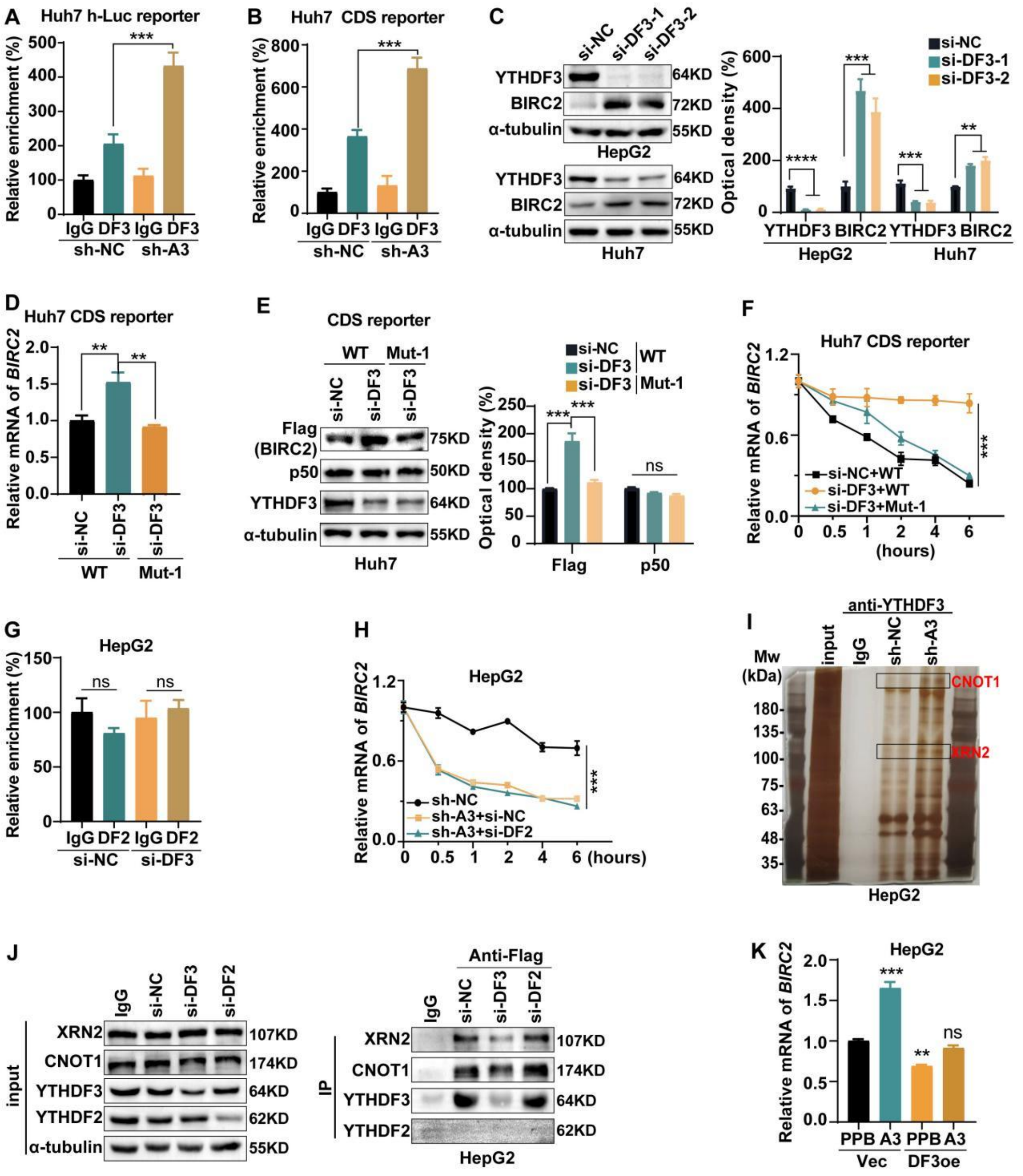

Figure S9 YTHDF3/CNOT1–XRN2 Complex is Involved in m^{1}A-Regulated Stability of BIRC2 mRNA

Related to Figure 6

(A) CLIP-qPCR analysis of BIRC2 mRNA from h-Luc reporter in sh-NC and sh-A3 Huh7 cells using an anti-YTHDF3 antibody.

(B) CLIP-qPCR analysis of BIRC2 mRNA from CDS reporter in sh-NC and sh-A3 Huh7 cells using an anti-YTHDF3 antibody.

(C) Western blot analysis of BIRC2 and YTHDF3 expression in HepG2 and Huh7 cells

transfected with si-NC, si-YTHDF3-1, or si-YTHDF3-2 (left). Band intensities were quantified using ImageJ, and normalized to α-Tubulin (right).

(D) BIRC2 mRNA levels from CDS reporter in Huh7 cells co-transfected with WT or Mut-1 CDS reporter and si-NC or si-YTHDF3, measured by RT-qPCR and normalized to endogenous BIRC2 and GAPDH.

(E) Western blot analysis of exogenous Flag-BIRC2 expression in Huh7 cells co-transfected with WT or Mut-1 CDS reporter and si-NC or si-YTHDF3 (left). Band intensities were quantified using ImageJ, and normalized to α-Tubulin (right).

(F) BIRC2 mRNA stability from CDS reporter in Huh7 cells co-transfected with WT or Mut-1 CDS reporter and si-NC or si-YTHDF3, followed by Act-D (1 μg/mL) treatment for the indicated times, measured by RT-qPCR and normalized to 18S.

(G) CLIP-qPCR analysis of BIRC2 mRNA enrichment in HepG2 cells transfected with si-NC or si-YTHDF3, using an anti-YTHDF2 antibody.

(H) BIRC2 mRNA stability in sh-NC and sh-A3 HepG2 cells transfected with si-NC or si-YTHDF2 and treated with Act-D (1 μg/mL) for the indicated times, measured by RT-qPCR and normalized to 18S.

(I) Silver-stained PAGE gel following YTHDF3 pull-down assay.

(J) Interactions between BIRC2 mRNA (CDS reporter) and YTHDF3, XRN2, CNOT1, or YTHDF2 in si-NC, si-YTHDF3, and si-YTHDF2 HepG2 cells, analyzed by Flag antibody immunoprecipitation.

(K) BIRC2 mRNA expression in HepG2 cells co-transfected with PPB or ALKBH3 and empty vector or YTHDF3 constructs for 24 hours, measured by RT-qPCR and normalized to GAPDH.

Data are presented as mean ± SD. Statistical analyses: (A–C, E, H, I, L) unpaired t-test (n = 3); (G) two-way ANOVA with Bonferroni's test (n = 3); (K) representative of three independent experiments. *$p < 0.05$, **$p < 0.01$, ***$p < 0.001$; ns, not significant.

Figure S10

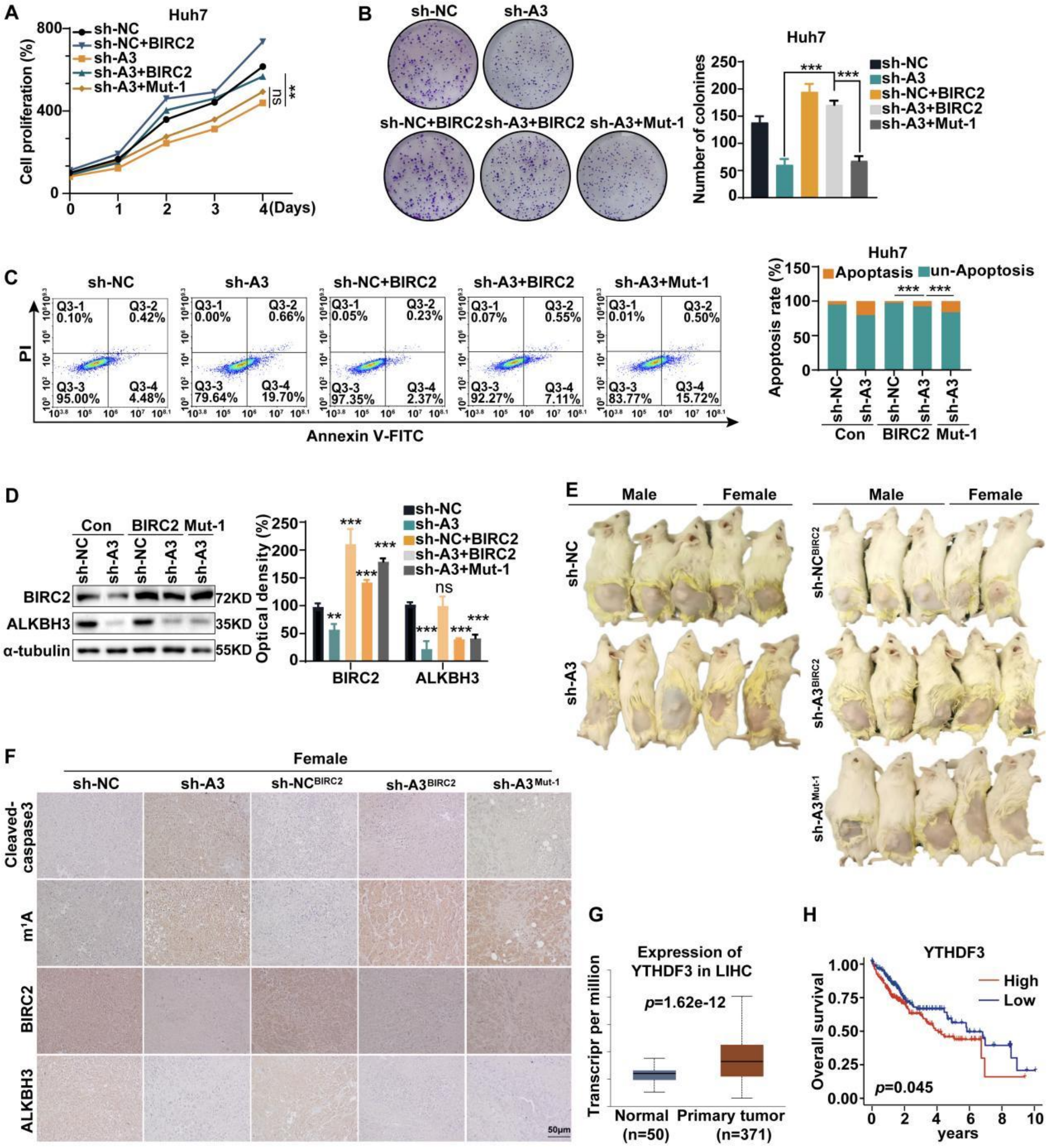

Figure S10 m^{1}A/BIRC2 Axis Regulates Liver Cancer Progression

Related to Figure 7

(A) Relative cell proliferation in sh-NC and sh-A3 Huh7 cells transfected with empty vector, BIRC2-WT, or BIRC2-Mut-1 constructs, measured by CCK-8 assay.

(B) Colony formation ability in sh-NC and sh-A3 Huh7 cells transfected as in (A).

(C) Apoptosis levels in sh-NC and sh-A3 Huh7 cells transfected as in (A), assessed by Annexin V/PI staining and flow cytometry. Representative flow cytometry plots (left) and quantitative analysis of the total apoptosis rate (right) are shown.

(D) Western blot analysis of BIRC2 and ALKBH3 expression in sh-NC and sh-A3 HepG2

cells transfected with empty vector, BIRC2-WT, or BIRC2-Mut-1 constructs (left). Band intensities were quantified using ImageJ, and normalized to α-Tubulin (right).

(E) Xenograft tumor model in NCG immunodeficient mice.

(F) Immunohistochemical (IHC) staining of Cleaved caspase-3, m^1A, BIRC2, and ALKBH3 in paraffin-embedded sections from xenograft tumor tissues. Scale bar: 50 μm (×200).

(G) YTHDF3 mRNA expression in liver tumor and normal tissues from the TCGA-LIHC dataset.

(H) Kaplan–Meier survival curves of overall survival (OS) based on YTHDF3 expression in TCGA-LIHC patients.

Data are presented as mean ± SD. Statistical analyses: (A–C) two-way ANOVA with Tukey's test (n = 3); (G) unpaired t-test; (H) log-rank test. *$p < 0.05$, **$p < 0.01$, ***$p < 0.001$; ns, not significant.

MATERIALS AND METHODS

Cellular Models and Genetic Manipulation

Human liver cancer cell lines (Huh7, HEP3B, HepG2, HLF, MHCC-97H, and HCCLM3) were cultured in Minimum Essential Medium (MEM) supplemented with 10% fetal bovine serum (FBS) at 37 °C in a 5% CO_2 atmosphere. The normal hepatocyte line THLE-2 was obtained from Shanghai Anwei Biotechnology Co., Ltd. (Shanghai, China) and maintained in Bronchial Epithelial Cell Growth Medium (BEGM™ BulletKit™; CC-3170, Lonza/Clonetics Corporation, Walkersville, MD, USA) according to the manufacturer's instructions. Prior to experiments, all cell lines were authenticated using short tandem repeat (STR) profiling (Shanghai Biowing Applied Biotechnology Co., Ltd.) and tested for mycoplasma contamination using the Myco-Blue Mycoplasma Detector (Vazyme), with negative results.

Stable ALKBH3-knockdown cell lines (Huh7, HEP3B, and HepG2 backgrounds) were generated via lentiviral transduction (Obio Technology), followed by selection with 1 μg/mL puromycin for 14 days. Knockdown efficiency was confirmed in three independent experiments using quantitative RT–PCR and western blot analysis.

Cells were first maintained under the indicated treatment or transfection conditions. Apoptosis was then induced by treating the cells with a combination of 10 ng/mL TNF-α and 100 nM SM-164 (Beyotime; diluted 1:1000 as recommended) for 6 hours [2]. All experiments were performed in three independent biological replicates, with consistent sample sizes detailed in the respective figure legends.

Plasmids, Mutagenesis, siRNA, and Transfection

Plasmids used for transient overexpression included: PPB (a mammalian expression vector from Dr. Chuan He's lab) [3, 4], PPB/ALKBH3, PPB/ALKBH3-R122S, PPB/ALKBH3-L177A, pcDNA3.1-BIRC2, pcDNA3.1-5′UTR-CDS-3×Flag(BIRC2), pcDNA3.1-5′UTR-Mut1/2/3-CDS-3×Flag(BIRC2), and pcDNA3.1-NFKB1-HA. Sequences of siRNAs and shRNAs, as well as primers used for site-directed mutagenesis, are provided in Supplementary Tables S2 and S3, respectively.

Plasmid and siRNA transfections were carried out using Lipofectamine 3000 reagent (Invitrogen), following the manufacturer's protocol. The working concentration of siRNA was 50 nM. All transfection experiments were performed in three independent biological

replicates, with consistent sample sizes per group as indicated in the corresponding figure legends.

Cell Proliferation Assay

Liver cancer cells were seeded in 96-well plates at a density of 3 × 10^3 cells per well in medium supplemented with 10% FBS. After treatment under the indicated conditions, cell proliferation was assessed using the CCK-8 cell viability assay system (Dojindo) according to the manufacturer's instructions and previously established protocols [3, 4]. Each experimental group included six technical replicates, and the entire assay was independently repeated three times. Data are expressed as mean ± standard deviation (SD).

Colony Formation Assay

Liver cancer cells were seeded in 6-well plates at a density of 1000 cells per well and cultured in complete medium at 37 °C with 5% CO_2 for 14 days. Following incubation, cells were fixed with 4% paraformaldehyde for at least 1 hour and stained with 1% crystal violet solution. After three washes with phosphate-buffered saline (PBS), the stained colonies were photographed. Colony numbers were quantified using ImageJ software by analyzing images from three independent experiments, each performed with triplicate wells. Data are presented as mean ± SD.

Annexin V/PI Staining for Apoptosis Assay

The percentage of apoptotic cells was determined using an Annexin V-FITC/PI apoptosis detection kit (Beyotime) according to the manufacturer's instructions. Briefly, transfected Huh7, HepG2, or HEP3B cells were seeded in 6-well plates at a density of 1×10^5 cells per well and cultured under specified conditions. After treatment, cells were harvested, washed twice with cold PBS, and resuspended in 1× binding buffer. Subsequently, 1×10^5 cells were stained with 5 µL of FITC Annexin V and 5 µL of propidium iodide (PI) for 15 minutes at room temperature in the dark. Apoptosis was assessed within 1 hour using a NovoCyte flow cytometer (Agilent).

Apoptotic cells were defined as the sum of both early apoptotic (Annexin V+/PI−) and late apoptotic (Annexin V+/PI+) populations. Representative gating strategies, including quadrant settings based on unstained and single-stained controls. Each experiment was independently repeated three times with consistent results.

Western Blot Analysis

Western blot analysis was performed as previously [3, 4] described with the following details. After treatment as indicated in the figure legends, cells were lysed using Cell Lysis Buffer for Western Blot and IP (Beyotime) for 30 minutes on ice. Total protein concentration was determined using a BCA assay kit. Approximately 40 μg of protein per sample were separated by sodium dodecyl sulfate–polyacrylamide gel electrophoresis (SDS-PAGE) and transferred to polyvinylidene fluoride (PVDF) membranes. The membranes were blocked with 5% non-fat milk in PBST for 2 hours at room temperature and then incubated overnight at 4 °C with the following primary antibodies (BIRC2, Abcam, ab108361; ALKBH3, Merck Millipore, 09-882; YTHDF1, Proteintech, 17479-1-AP; YTHDF2, Proteintech, 24744-1-AP; YTHDF3, Proteintech, 25537-1-AP; YTHDC1, Proteintech, 29441-1-AP; α-tubulin, Proteintech, 66031-1-Ig; Caspase 9, Proteintech , 10380-1-AP; Cleaved caspase 9, Proteintech, 10380-1-AP; Caspase 7, Proteintech, 27155-1-AP; Cleaved caspase 7, Proteintech, 27155-1-AP; Caspase 3, Proteintech, 19677-1-AP; Cleaved caspase 3, Cell Signaling Technology, 9661S; Bcl-2, Proteintech, 12789-1-AP; BAX, Proteintech 50599-2-Ig; Cytochrome c, Proteintech, 10993-1-AP; NFKB1, Beyotime; AF1246; GAPDH, Baijia, IDS0106; XRN2, Proteintech, 11267-1-AP; CNOT1, Proteintech, 14276-1-AP; DDX6, Proteintech, 14632-1-AP; Lamin B1, Proteintech, 12987-1-AP).

After washing three times with PBST, the membranes were incubated with horseradish peroxidase (HRP)-conjugated secondary antibodies (1:5000 dilution) for 2 hours at room temperature. Protein bands were visualized using a Western Blotting Plus Chemiluminescence Reagent (Life Science) and imaged with a chemiluminescence imaging system. Band intensities were quantified using ImageJ software (National Institutes of Health). Each experiment was repeated independently at least three times ($n \geq 3$). Statistical analysis between groups was performed using a two-tailed Student's t-test or one-way ANOVA with Bonferroni correction, as appropriate, with details provided in the corresponding figure legends.

Dot-Blot Assay

The dot-blot assay was performed as previously [4] described with the following modifications . Briefly, mRNA was enriched using the GenElute mRNA Miniprep Kit

(Sigma-Aldrich) followed by the RiboMinus Transcriptome Isolation Kit (Human/Mouse; Thermo Fisher Scientific). After denaturation at 70 °C for 5 minutes, serially diluted mRNA samples (ranging from 2000 ng to 500 ng per dot; n = 3 independent biological replicates per sample group) were spotted onto an Amersham Hybond N+ membrane (GE Healthcare). The RNA was crosslinked to the membrane using a UV crosslinker with auto-crosslinking mode (3 cycles).

The membrane was blocked with 5% non-fat milk in 1× PBST for 1 hour at room temperature and then incubated overnight at 4 °C with a rabbit anti-m^1A primary antibody (MBL, D345-3; dilution 1:1000). After three washes with 1× PBST, the membrane was incubated with a horseradish peroxidase (HRP)-conjugated anti-rabbit IgG secondary antibody (Cell Signaling Technology; dilution 1:5000) for 1 hour at room temperature. Signals were detected using a 3,3′-diaminobenzidine (DAB) peroxidase substrate kit (Yeasen Biotechnology) and imaged with a chemiluminescence documentation system.

Densitometric analysis of dot intensities was performed using ImageJ software. Statistical comparisons between groups were made using a two-tailed Student's t-test, with details provided in the corresponding figure legend.

RNA extraction and quantitative real-time PCR

Total RNA was extracted using TRIzol reagent (Takara) according to the manufacturer's instructions. RNA concentration and purity were determined nanodrop2000 (Thermo Fisher Scientific). Complementary DNA (cDNA) was synthesized from 1 μg of total RNA using a PrimeScript RT reagent kit (Takara). Quantitative real-time PCR (qRT-PCR) was performed using TB Green Premix Ex Taq II (Takara) on a QuantStudio 6 Pro Real-Time PCR System (Applied Biosystems). Each reaction was performed in technical triplicates, and all experiments were repeated in at least three independent biological replicates ($n \geq 3$).

The PCR amplification conditions were as follows: initial denaturation at 95 °C for 30 seconds, followed by 40 cycles of 95 °C for 5 seconds and 60 °C for 30 seconds. Melting curve analysis was conducted to confirm amplification specificity.

GAPDH mRNA was used as an internal control for normalization under standard conditions. For samples treated with actinomycin D (Act-D), 18S rRNA was used as the normalization

control due to its higher stability under transcriptional inhibition. The primer sequences used for qRT-PCR are listed in Supplementary Table S3.

Gene expression levels were calculated using the $2^{-\Delta\Delta Ct}$ method. Statistical comparisons between groups were performed using a two-tailed Student's t-test or one-way ANOVA with Bonferroni's post-hoc test, as specified in the figure legends. Data are presented as mean ± SD.

Nuclear Run-On Assay

Nuclear run-on (NRO) assays were performed as previously described [3, 4, 5] with the following modifications. Briefly, cells were counted and plated in 10-cm dishes. After one wash with ice-cold PBS, cells were harvested in 4 mL of lysis buffer (10 mM Tris-HCl, pH 7.4; 10 mM NaCl; 3 mM $MgCl_2$; 150 mM sucrose; 0.5% NP-40) and incubated on ice for 10 min. Nuclei were pelleted by centrifugation at 500 g for 5 min at 4 °C, then resuspended in 100 μL of freezing buffer (50 mM Tris-HCl, pH 8.3; 5 mM $MgCl_2$; 0.1 mM EDTA; 40% glycerol) and stored at −80 °C if not used immediately.

For NRO transcription, nuclei were thawed and incubated in 100 μL transcription reaction buffer containing 1× biotin-16-UTP labeling mix (Roche) for 30 min at 29 °C. Reactions were stopped by adding 500 μL of TRIzol LS reagent (Takara). Total RNA was extracted using the Maxwell RSC Instrument with the LEV RNA Kit (Promega). Biotin-labeled nascent RNA was isolated using 50 μL of Dynabeads M-280 Streptavidin (Invitrogen) resuspended in binding buffer (10 mM Tris-HCl, pH 7.5; 1 mM EDTA; 2 M NaCl) and incubated for 30 min at room temperature with rotation. Beads were washed three times with PBST containing 8 U/mL RNaseOUT (Invitrogen). Bound RNA was eluted and extracted with TRIzol, followed by quantification using quantitative RT-PCR as described above.

Each experiment included three independent biological replicates (n = 3). Statistical analysis was performed using a two-tailed Student's t-test or one-way ANOVA with Bonferroni correction, as appropriate, with details provided in the corresponding figure legend.

Luciferase Reporter Assay/Dual-Luciferase Reporter Assay

Promoter activity and translation efficiency were assessed using dual-luciferase reporter assays. For promoter activity analysis, the pEGX-DL01-BIRC2-hluc-Rluc reporter plasmid (GeneCodex, Wuhan, China), which contains the BIRC2 promoter region (−2000 to +1)

upstream of the hLuc (firefly luciferase) gene, was employed. For translation activity analysis, the pEGX-DL02-hluc-BIRC2-Rluc reporter (GeneCodex), which contains the BIRC2 coding sequence (CDS) downstream of the hLuc gene, was used. To evaluate the effects of the wild-type or mutant BIRC2 5′UTR (Mut1/2/3) on mRNA stability, corresponding fragments were inserted into the pEGX-DL02 vector between the promoter and the hLuc reporter gene. Cells were transfected with the respective reporter constructs for 48 hours. Luciferase activity was measured using the Dual-Glo Luciferase Assay System (Beyotime) according to the manufacturer's instructions. Firefly luciferase (hLuc) activity was normalized to Renilla luciferase (Rluc) activity to account for variations in transfection efficiency and general transcriptional/translational activity.

For mRNA stability assays, transcript levels of hLuc and Rluc in the pEGX-DL02 system were quantified via qRT-PCR using the following primers: h-Luc, forward 5′- TGG ACA TCA CCT ATG CCG AGT AC -3′ and reverse 5′- GAT GCG GTG GTT GGT GTT CAG -3′; R-Luc, forward 5′- AAG GAG AAG GGC GAG GTT AGA C -3′ and reverse 5′- CCG AAG GTA GGC GTT GTA GTT G -3′.

All transfections and assays were performed with six independent biological replicates (n = 6). Data are presented as mean ± SD. Statistical significance was determined using a two-tailed Student's *t*-test or one-way ANOVA with Bonferroni's post-hoc test, as appropriate, with specific tests indicated in the figure legends.

RNA Stability and Protein Stability Assays

To assess RNA stability, shNC and shALKBH3 liver cancer cells were seeded overnight and treated with 1 μM actinomycin D (Act-D; Sigma, SBR00013) for the indicated durations (e.g., 0, 2, 4, 8 hours). Total RNA was extracted using TRIzol reagent, and the mRNA levels of BIRC2, hLuc, and BIRC2 precursor mRNA (pre-mRNA) were quantified by quantitative real-time PCR (qRT–PCR). GAPDH mRNA was used as an internal control for normalization. RNA decay rates were calculated relative to the untreated control (t = 0).

For protein stability analysis, cells were treated with 100 μg/mL cycloheximide (CHX; Selleck, S7418) or 2 μM MG132 (Selleck, E2899) for specified time points (e.g., 0, 2, 4, 8 hours). CHX inhibits de novo protein synthesis, while MG132 blocks proteasomal degradation. After treatment, cells were lysed in 100 μL of cell lysis buffer (Beyotime)

supplemented with protease inhibitors. BIRC2 protein levels were detected by western blotting, with α-tubulin or GAPDH serving as loading controls. Band intensities were quantified using ImageJ software.

Each experiment included at least three independent biological replicates ($n \geq 3$). Data are presented as mean ± SD. Half-life ($t_{1/2}$) was calculated using one-phase decay nonlinear regression analysis. Statistical comparisons were performed using two-way ANOVA with Bonferroni's multiple comparisons test, as detailed in the figure legends.

Subcellular Fraction

Subcellular fractionation was performed using the Nuclear and Cytoplasmic Extraction Kit (Beyotime) according to the manufacturer's instructions, as previously described [2]. Briefly, 2×10^7 cells were pelleted, washed with ice-cold PBS, and subjected to fractionation to isolate cytoplasmic and nuclear components.

Total RNA from each fraction was extracted using TRIzol reagent (Takara) following established protocols [1, 2]. The distribution of BIRC2 mRNA in cytoplasmic and nuclear fractions was analyzed by quantitative RT–PCR. GAPDH mRNA and 18S rRNA were used as controls for cytoplasmic and nuclear RNA, respectively.

For protein analysis, cytoplasmic and nuclear extracts were immunoblotted as described in the Western Blot Analysis section. BIRC2 protein levels in each fraction were detected using specific antibodies. α-Tubulin and Lamin B1 served as controls for cytoplasmic and nuclear protein fractions, respectively.

Each experiment was repeated in three independent biological replicates ($n = 3$). Statistical comparisons were performed using a two-tailed Student's t-test or one-way ANOVA, as appropriate, with details provided in the corresponding figure legend.

CLIP-RT-PCR Assay

The CLIP-RT-PCR assay was performed as previously described [2] with the following modifications. Briefly, cells were UV cross-linked (254 nm, 150 mJ/cm^2) on ice for 30 minutes before harvesting. Cells were lysed in high-salt lysis buffer (300 mM NaCl, 0.2% NP-40, 20 mM Tris-HCl pH 7.6, 0.5 mM DTT) supplemented with protease inhibitor cocktail (one tablet per 50 mL) and RNase inhibitor (1:200 dilution) at 4 °C for 30 minutes. Lysates were clarified by centrifugation at 12,000 g for 15 minutes at 4 °C.

The supernatant was treated with or without 1 U of RNase T1 (Thermo Fisher Scientific) at 4 °C for 15 minutes. Ten percent of the supernatant was saved as input control. The remainder was incubated with antibodies against YTHDF1, YTHDF2, YTHDF3, YTHDC1, XRN2, CNOT1, DDX6, or control IgG conjugated to Dynabeads™ Protein G (Thermo Fisher Scientific) in 500 µL of 1× IP buffer (150 mM NaCl, 10 mM Tris-HCl pH 7.5, 0.1% NP-40) containing RNase inhibitors for 3 hours at 4 °C with rotation.

Beads were washed twice with IP buffer, twice with low-salt wash buffer (50 mM NaCl, 10 mM Tris-HCl pH 7.5, 0.1% NP-40), and twice with high-salt wash buffer (500 mM NaCl, 10 mM Tris-HCl pH 7.5, 0.1% NP-40). RNA was eluted by incubating beads in 100 µL elution buffer (5 mM Tris-HCl pH 7.5, 1 mM EDTA pH 8.0, 0.05% SDS, 20 mg/mL Proteinase K) for 2 hours at 50 °C with shaking. Eluted RNA was recovered by phenol:chloroform extraction and ethanol precipitation. RNA concentration was measured using the Qubit™ RNA HS Assay Kit (Thermo Fisher Scientific).

Both immunoprecipitated RNA and input RNA were used as templates for quantitative RT–PCR. Each experiment included three independent biological replicates (n = 3). Data are presented as mean ± SD. Statistical significance was determined using a two-tailed Student's t-test or one-way ANOVA with Bonferroni correction, as specified in the figure legends.

Immunoprecipitation Assay

Immunoprecipitation (IP) assays were performed as follows. Liver cancer cells were seeded in 10-cm plates and treated under the indicated conditions. Cells were then pelleted and lysed in 400 µL of 1× IP buffer (150 mM NaCl, 10 mM Tris-HCl pH 7.5, 0.1% NP-40) supplemented with protease inhibitor cocktail on ice for 30 minutes. Lysates were clarified by centrifugation at 12,000 g for 15 minutes at 4 °C.

A 20 µL aliquot of the supernatant was saved as input. The remaining lysate was incubated overnight at 4 °C with primary antibodies against BIRC2, YTHDF3, YTHDF2, ALKBH3, XRN2, CNOT1, DDX6, or control IgG. Protein A/G agarose beads (Santa Cruz Biotechnology, sc-2003) were added to the lysate–antibody mixture and incubated for 4 hours at 4 °C with rotation.

The beads were washed three times with IP buffer and once with PBS. Bound proteins were eluted by boiling the beads in 1× SDS loading dye for 10 minutes at 95 °C. Eluted proteins were analyzed by western blotting as described in the “Western Blot Analysis” section.

Each experiment included three independent biological replicates (n = 3). Representative results from one experiment are shown in the figures. Statistical analysis was performed using a two-tailed Student’s t-test, with details provided in the corresponding figure legends.

Transmission Electron Microscopy (TEM) for Analysis of Apoptotic Vesicles

Transmission electron microscopy (TEM) was employed to analyze apoptotic vesicles in Huh7 shNC, Huh7 shALKBH3, and Huh7 shALKBH3+BIRC2oe cell lines. Cells were harvested at 80% confluency and detached using trypsin. Cell pellets were fixed in electron microscopy fixative containing 2.5% glutaraldehyde and 100 mM phosphate buffer (pH 7.4) at room temperature for 30 minutes in the dark.

After initial fixation, samples were sent to Wuhan Servicebio Technology Co., Ltd., for further processing, including post-fixation with 1% osmium tetroxide, dehydration through a graded ethanol series, embedding in epoxy resin, ultrathin sectioning (70 nm), and staining with uranyl acetate and lead citrate. Processed sections were imaged using a transmission electron microscope.

Apoptotic vesicles and ultrastructural changes in cell morphology were assessed qualitatively. For each experimental group, a minimum of 30 cells were examined across three independent biological replicates (n = 3). Representative images are presented in the figures.

Xenograft tumor mouse model

HepG2 cells (5×10^6) stably transduced with sh-NC, shALKBH3 (sh-A3), pcDNA-BIRC2 (sh-NC^{BIRC2}), or co-transfected with pcDNA-BIRC2-5′UTR-WT (sh-$A3^{BIRC2}$), or co-transfected with pcDNA-BIRC2-5′UTR-Mut1 (sh-$A3^{Mut1}$) were injected subcutaneously into the flanks of 4–6-week-old NOD/ShiLtIGpt-Prkdc em26l2rgem26/Gpt (NCG) mice (Male:Female=3:2) (GemPharmatech Co.,Lta, China), respectively.

Tumor length (L) and width (W) were measured weekly using a vernier caliper. Tumor volume was calculated with the formula: $V=\pi/6\times L\times W^2$. Mice were euthanized on day 30 post-injection, and tumors were excised, weighed, and processed for further analysis.

A portion of each tumor was snap-frozen in liquid nitrogen and stored at –80 °C for RNA and protein extraction. The remaining tissue was fixed in 4% paraformaldehyde for 24 hours and embedded in paraffin for IHC analysis.

Each experimental group contained 5 mice (n = 5). Data are presented as mean ± standard deviation (SD). Statistical comparisons between groups over time were performed using two-way ANOVA with Bonferroni's post-hoc test. Final tumor weights and volumes were compared using one-way ANOVA followed by Tukey's multiple comparisons test, as detailed in the figure legends.

Immunohistochemistry (IHC) Assay

Formalin-fixed, paraffin-embedded xenograft tumor sections (4 μm thick) were deparaffinized in xylene and rehydrated through a graded ethanol series. Antigen retrieval was performed by heating slides in 10 mM sodium citrate buffer (pH 6.0) at 95–100 °C for 15 minutes. Endogenous peroxidase activity was quenched with 3% hydrogen peroxide in methanol for 15 minutes at room temperature.

Sections were blocked with 5% normal goat serum and 0.1% Triton X-100 in phosphate-buffered saline (PBS) for 1 hour at room temperature to prevent nonspecific binding. Primary antibodies against m^1A (MBL, D345-3, 1:500), BIRC2 (Abcam, ab108361, 1:200), ALKBH3 (Proteintech, 12292-1-AP, 1:50), or Cleaved Caspase 3 (Cell Signaling Technology, 9661S, 1:150) were applied and incubated overnight at 4 °C.

After washing with PBS, sections were incubated with a horseradish peroxidase (HRP)-conjugated secondary antibody (1:1000; Beyotime) for 1 hour at room temperature. Signal was developed using a diaminobenzidine (DAB) substrate kit (Yeasen Biotechnology), and sections were counterstained with hematoxylin.

Stained sections were imaged using a Nikon Eclipse Ni-U microscope. IHC staining was evaluated by two independent pathologists blinded to the experimental groups. For each sample, five random fields (200× magnification) were analyzed. The staining intensity and percentage of positive cells were scored semiquantitatively.

Each experimental group included five biological replicates (n = 5). Statistical analysis was performed using one-way ANOVA followed by Tukey's post-hoc test, with details provided in the figure legends.

References for supplementary data

1. Dominissini D, Nachtergaele S, Moshitch-Moshkovitz S, Peer E, Kol N, Ben-Haim MS, *et al.* The dynamic N^1-methyladenosine methylome in eukaryotic messenger RNA. Nature. 2016;530:441-446.
2. Chen W, Chen S, Yan C, Zhang Y, Zhang R, Chen M, *et al.* Allergen protease-activated stress granule assembly and gasdermin D fragmentation control interleukin-33 secretion. Nat Immunol. 2022;23:1021-1030.
3. Chen Z, Qi M, Shen B, Luo G, Wu Y, Li J, et al. Transfer RNA demethylase ALKBH3 promotes cancer progression via induction of tRNA-derived small RNAs. Nucleic Acids Res. 2019;47:2533-2545.
4. Wu Y, Chen Z, Xie G, Zhang H, Wang Z, Zhou J, *et al.* RNA m^1A methylation regulates glycolysis of cancer cells through modulating ATP5D. Proc Natl Acad Sci U S A. 2022;119:e2119038119.
5. Yamada M, Horiguchi K, Umezawa R, Hashimoto K, Satoh T, Ozawa A, *et al.* Troglitazone, a ligand of peroxisome proliferator-activated receptor-{gamma}, stabilizes NUCB2 (Nesfatin) mRNA by activating the ERK1/2 pathway: isolation and characterization of the human NUCB2 gene. Endocrinology. 2020;151:2494-2503.

Supplementary Table Legends

Supplementary Table S1. Background demographics of the study cohort

Clinical and pathological characteristics of 15 patients with liver cancer included in this study. Data include diagnosis, gender, age, primary organ involvement, metastatic sites, tumor grade, tumor size, neural invasion, lymph node and distant metastasis, TNM staging, hepatitis B virus serological markers (HBsAg, HBsAb, HBcAb, HBeAg, HBeAb), and serum AFP levels. Additional markers such as CA199 are indicated where applicable. This table provides the baseline information for the cohort used in the analysis of m^1A level and its correlation with clinicopathological features.

Supplementary Table S2. Sequences of shRNAs and siRNAs used in this study

The table lists the names and corresponding nucleotide sequences of short hairpin RNAs (shRNAs) and small interfering RNAs (siRNAs) targeting specific genes, including ALKBH3, YTHDF3, and YTHDF2. Non-targeting control sequences (sh-NC and siNC) were used as negative controls. For siRNAs, both sense and antisense strands are provided. All sequences are shown in the 5' to 3' orientation.

Supplementary Table S3. Sequences of primers used in this study

The table lists the names and nucleotide sequences of forward (sense) and reverse (anti-sense) primers employed for quantitative real-time PCR (qRT-PCR) and cloning experiments. Primers targeting housekeeping genes (GAPDH, ACTB, 18S) were used as internal controls. Gene-specific primers include those for ALKBH3, BIRC2 (full-length, precursor, and UTR regions), as well as primers for NFKB1, Renilla luciferase (R-Luc), firefly luciferase (h-Luc), and Flag-tag detection. All sequences are shown in the 5' to 3' orientation.

Supplementary Table S4. YTHDF3 pull-down and mass spectrometry analysis

YTHDF3 pull-down followed by mass spectrometry was performed in Huh7 cells. The table lists proteins identified in the YTHDF3 pull-down sample (sh-A3) compared to the negative control (sh-NC), with normalized abundance values shown for each condition. A total of 333 proteins were found to be enriched in sh-A3 cells compared with sh-NC cells. Proteins with a sh-A3/sh-NC abundance ratio greater than 2 are highlighted in the rightmost column. Accession numbers and gene symbols are provided for each identified protein. This dataset reveals potential YTHDF3-interacting partners involved in m^1A-mediated RNA metabolism and tumor progression.

Supplementary Table S5. Gene Set Variation Analysis (GSVA) of m^1A modification subtypes in TCGA-LIHC cohort

GSVA was performed to assess the enrichment of biologically relevant gene sets across the three m^1A modification subtypes (Clusters A-C) identified by consensus clustering of 10 m^1A regulators in 371 TCGA-LIHC patients. The table lists representative genes from key pathways showing differential activation among subtypes, including RNA degradation,

mitophagy (animal), NF-κB signaling pathway, apoptosis, and hepatocellular carcinoma-related gene sets. This analysis revealed coordinated activation of apoptosis-related pathways alongside dysregulation of mitophagy and NF-κB signaling, corresponding to Figure 2B.

Supplementary Table S6. List of m^{1}A-modified genes identified by m^{1}A-seq in HepG2 cells

The table presents genes previously identified as m^{1}A-methylated targets through m^{1}A sequencing (m^{1}A-seq) analyses in HepG2 cells, compiled from published studies [1]. For each gene, the RefSeq accession number (NM_), gene symbol, species (Homo sapiens*), and full gene name are provided. This dataset was used to identify overlapping candidates between m^{1}A-modified genes and apoptosis-related genes from GSVA enrichment analysis, as shown in Figure 3A.

Supplementary Table S7. Gene Ontology (GO) enrichment analysis of YTHDF3-interacting proteins identified by mass spectrometry

The table presents the top enriched pathways from GO analysis of the 333 proteins identified as YTHDF3-interacting candidates in Huh7 cells (corresponding to Figure S9I and Supplementary Table S4). For each enriched pathway, the associated genes are listed. Notably, the RNA degradation pathway was significantly enriched and included eight known regulators of mRNA stability (highlighted in Figure 6F). Other enriched pathways include ribosome, DNA replication, RNA transport, carbon metabolism, spliceosome, biosynthesis of amino acids, mismatch repair, nucleotide excision repair, and citrate cycle (TCA cycle). This analysis provides insights into the functional landscape of YTHDF3-interacting proteins and their potential roles in mRNA metabolism and tumor progression.
