# Supplementary material for: m1A-mediated regulation of BIRC2 mRNA stability drives apoptosis evasion and tumor progression in liver cancer
Source: Cell Death Dis. 2026 Apr 16;17(1):630. doi: 10.1038/s41419-026-08731-z (PMC13350948; doi:10.1038/s41419-026-08731-z)

Figure 1A

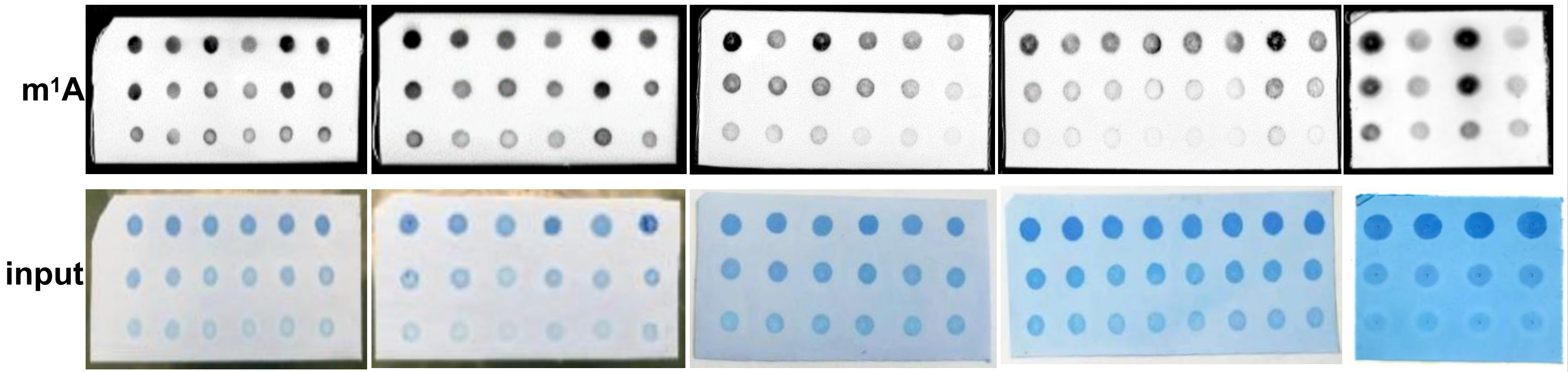

Figure 1F

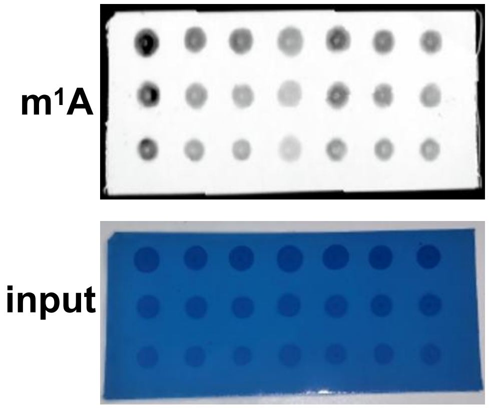

Figure 2D

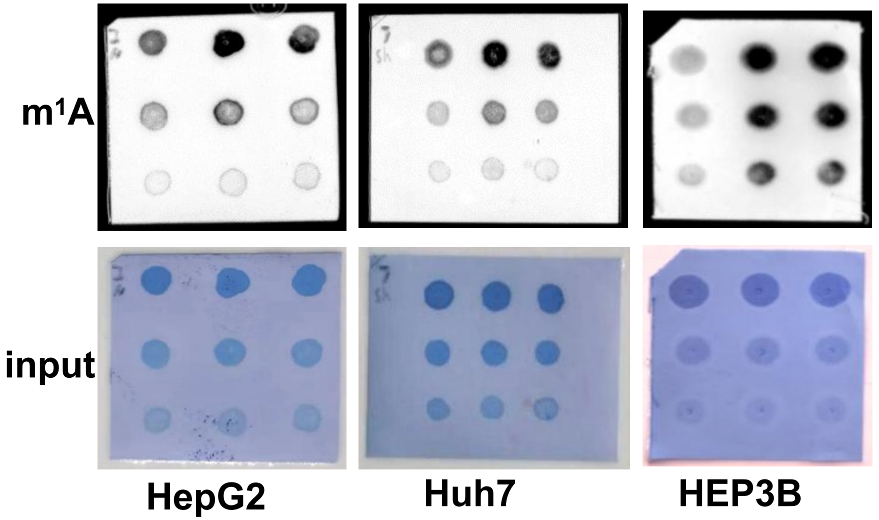

Figure 2J

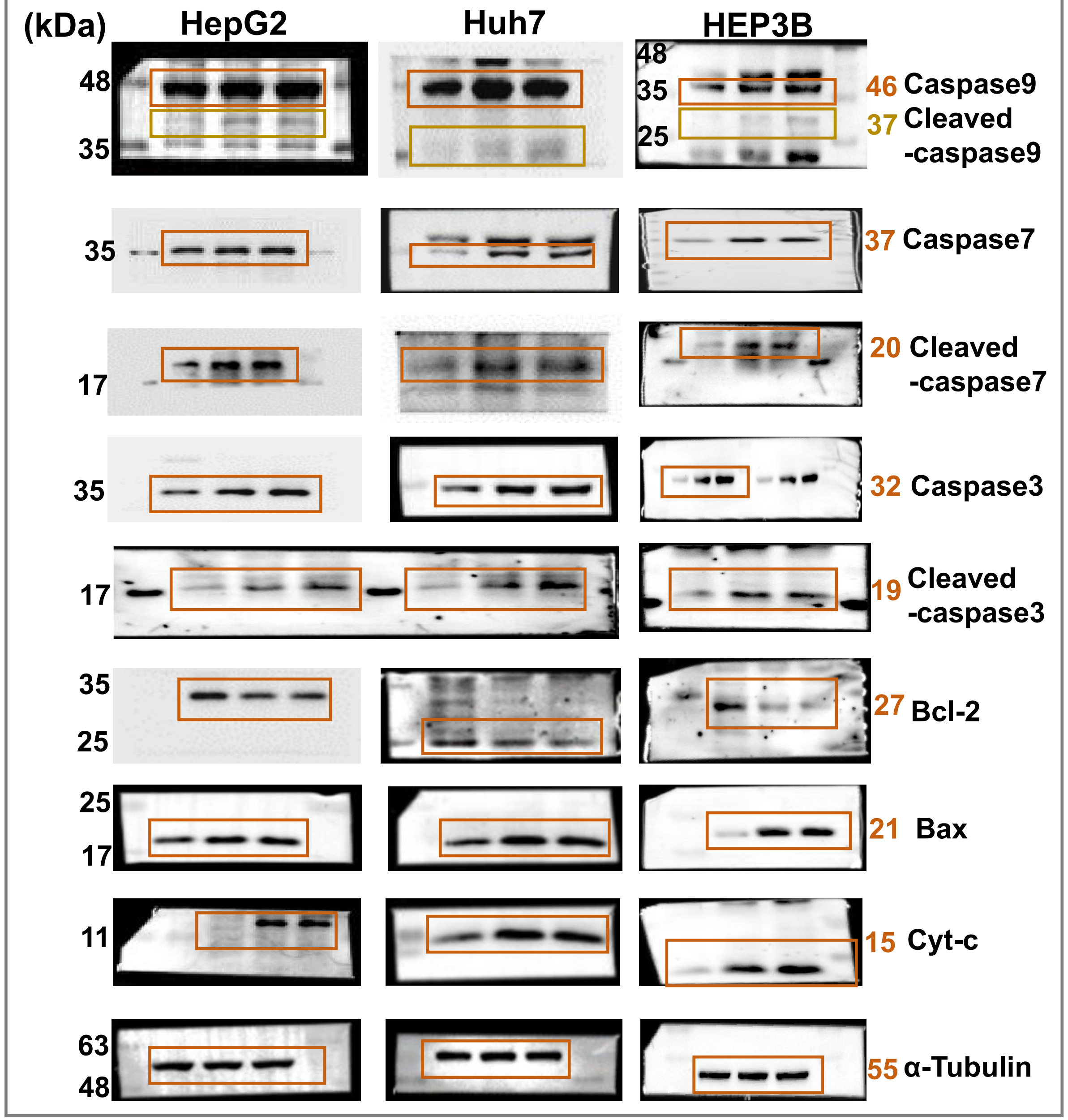

Figure 2L

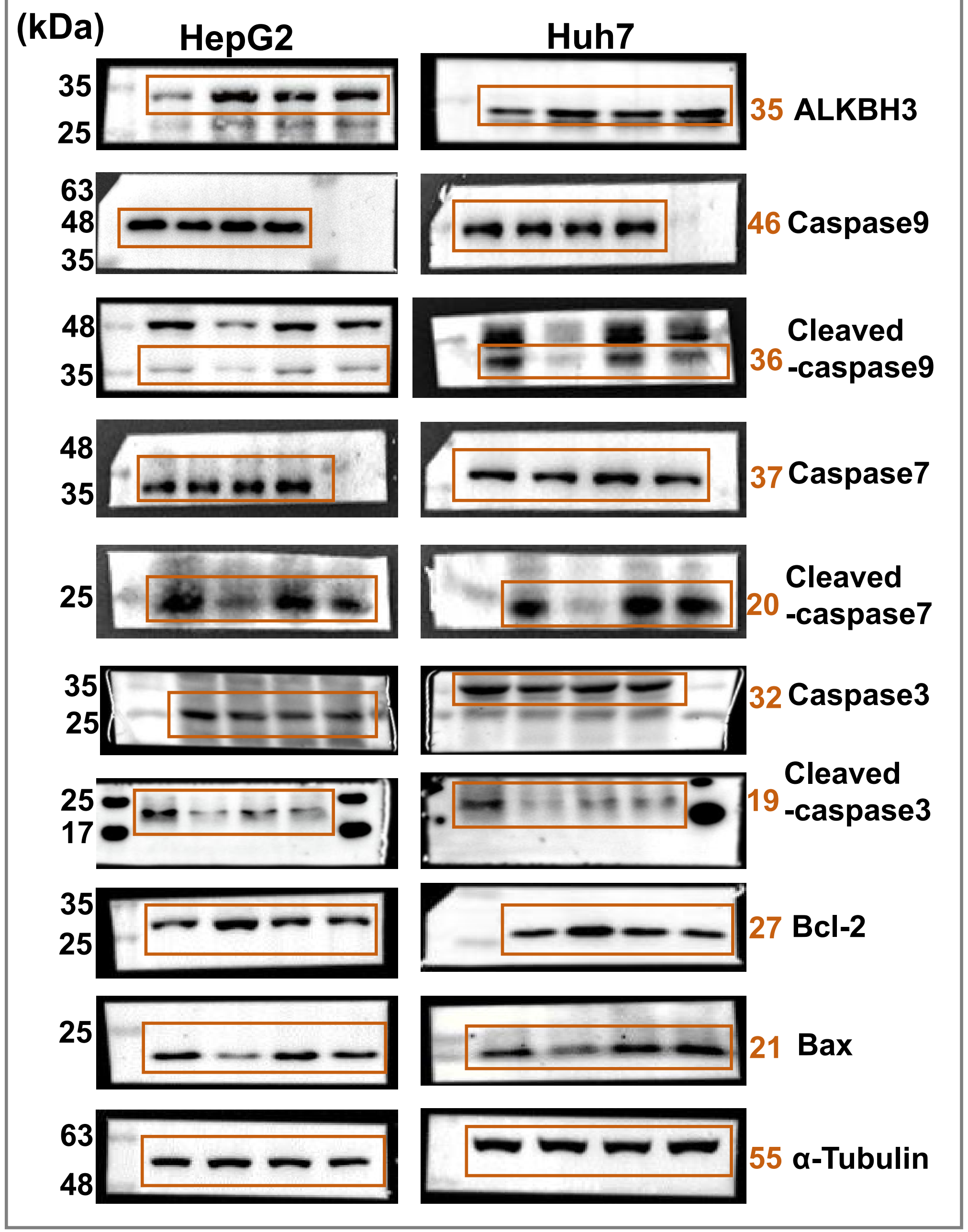

Figure 3C

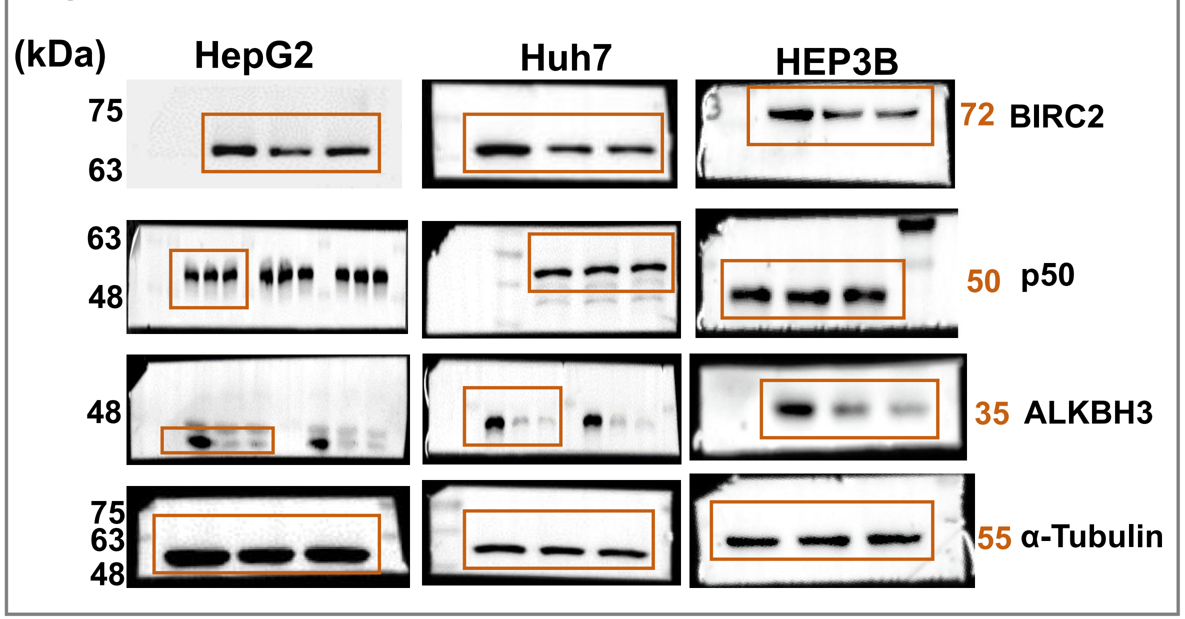

Figure 3H

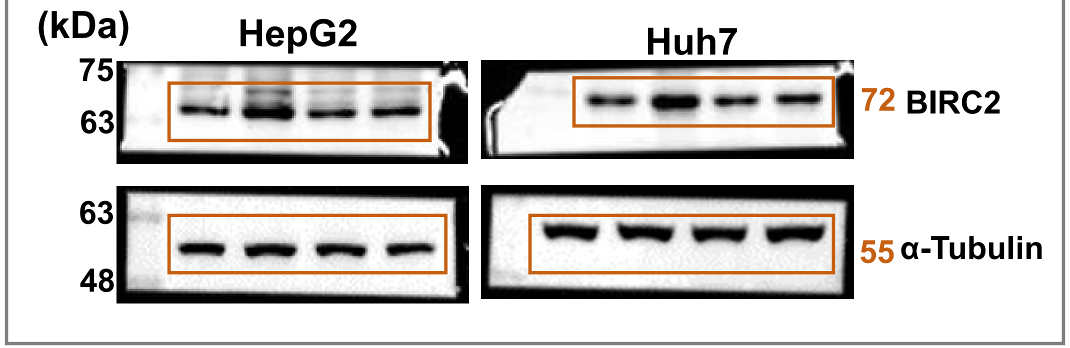

Figure 3D

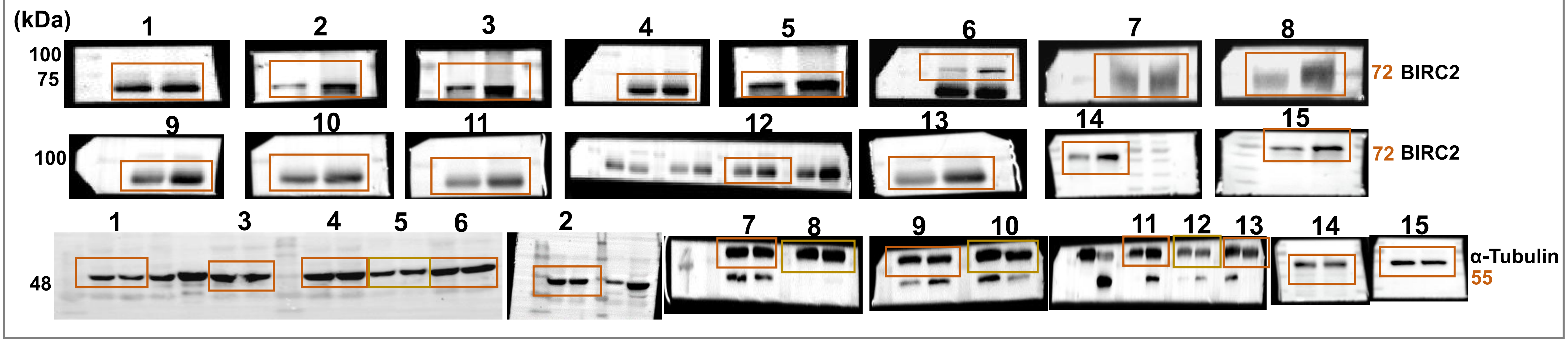

Figure 3J

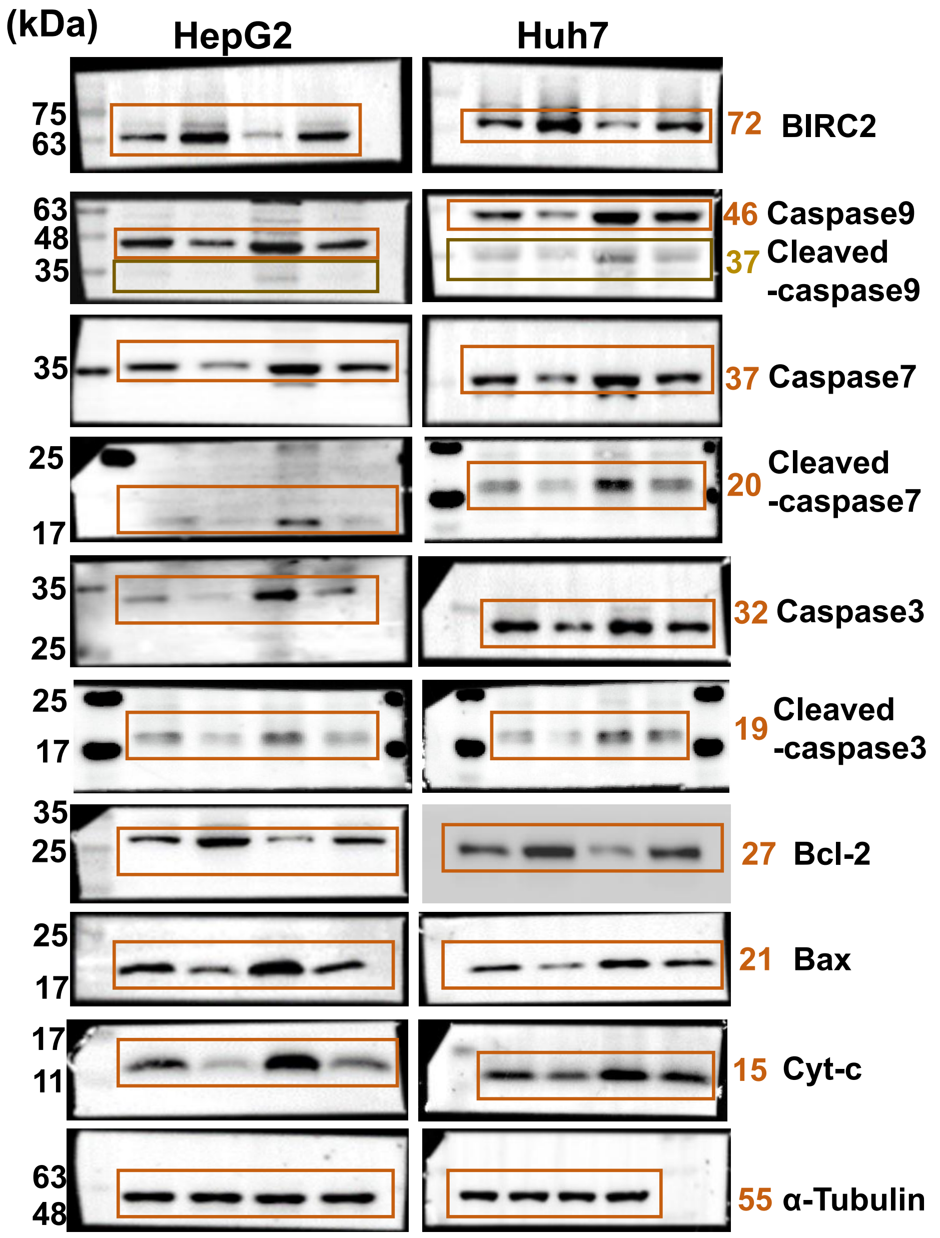

Figure 4G

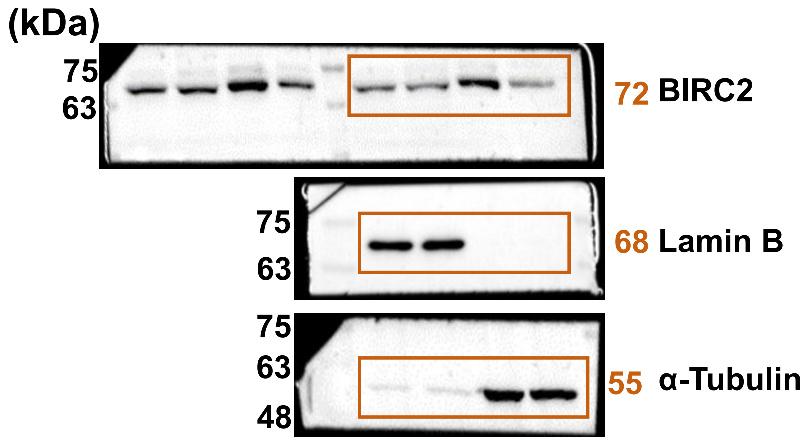

Figure 4K

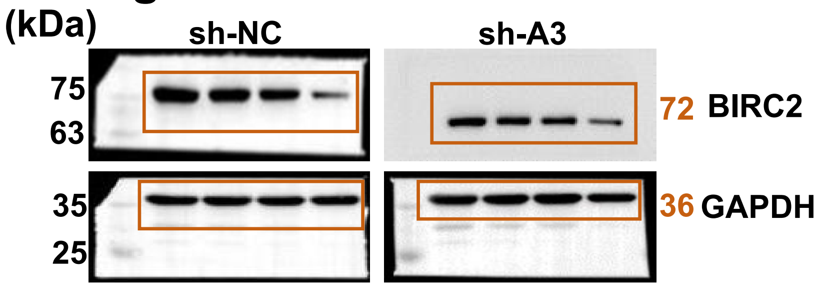

Figure 4L

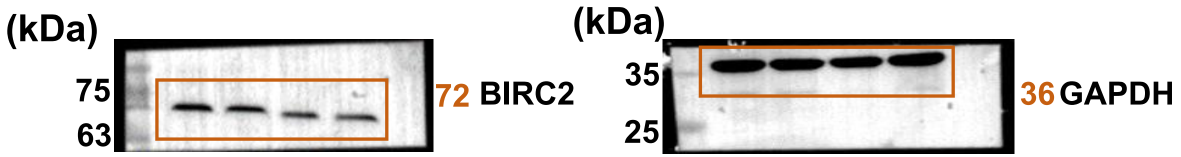

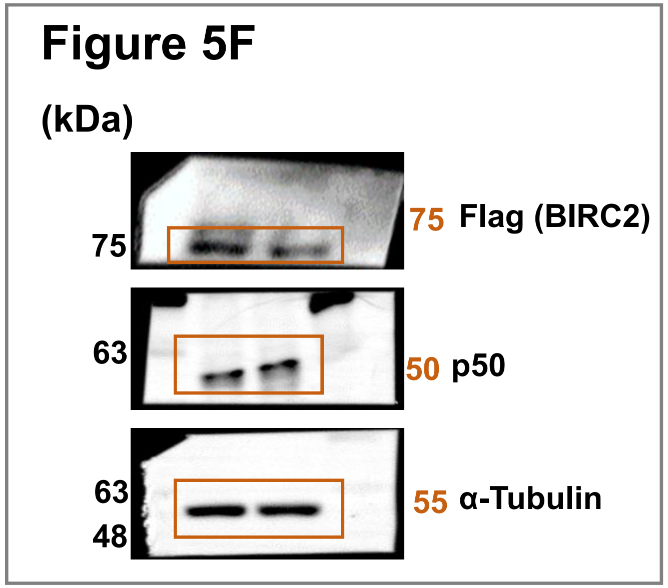
Figure 5F
(kDa)
75
75 Flag (BIRC2)
63
50 p50
63
48
55 α-Tubulin

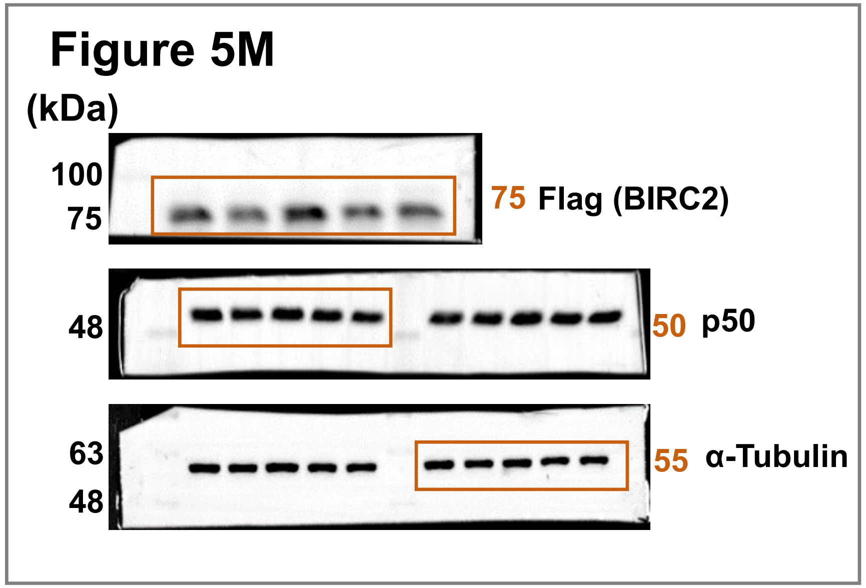
Figure 5M
(kDa)
100
75
75 Flag (BIRC2)
48
50 p50
63
48
55 α-Tubulin

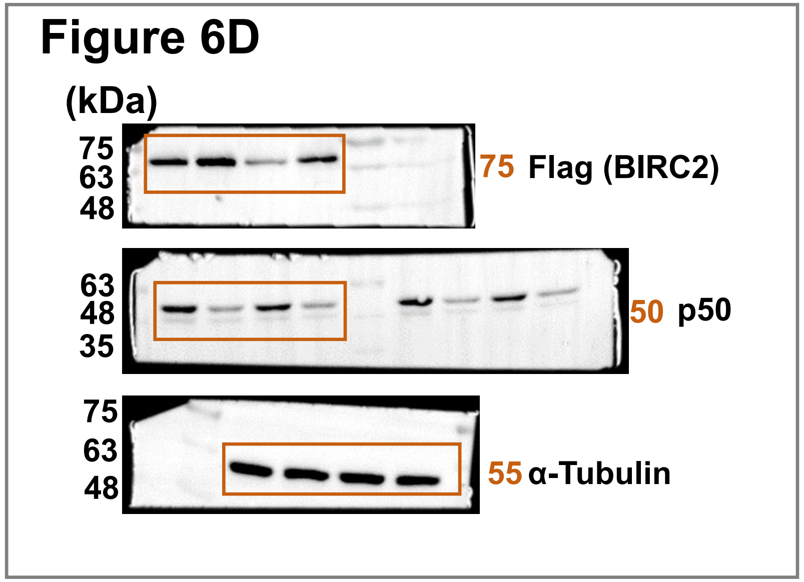
Figure 6D
(kDa)
75
63
48
75 Flag (BIRC2)
63
48
35
50 p50
75
63
48
55 α-Tubulin

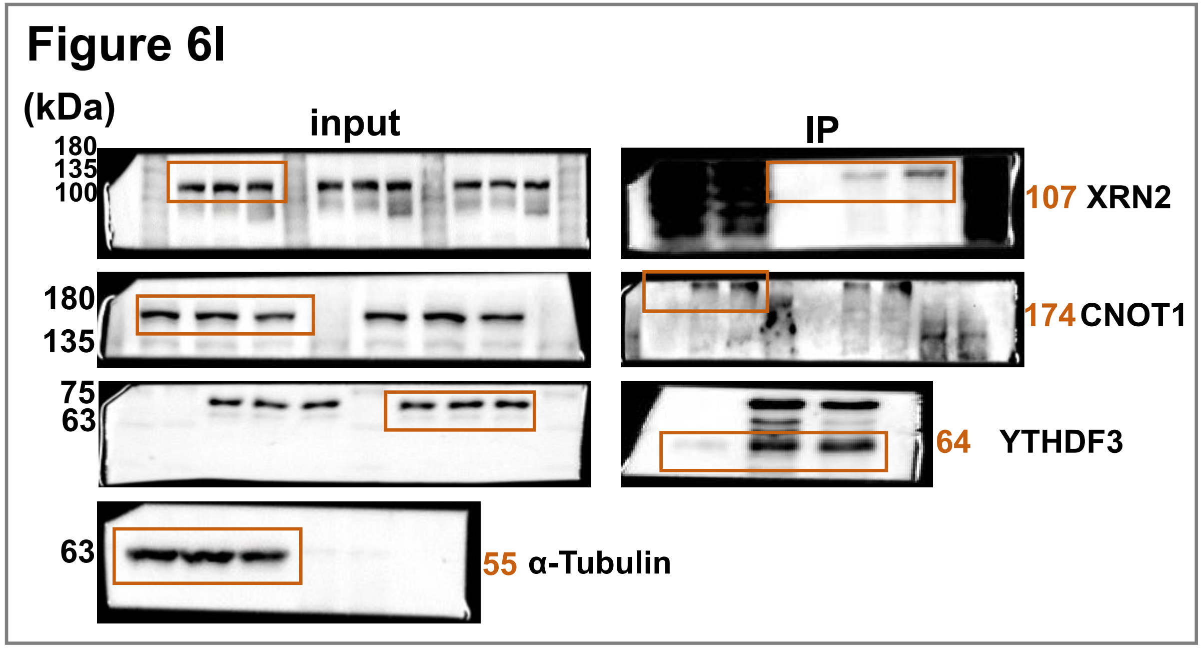
Figure 6I
(kDa)
input
IP
180
135
100
107 XRN2
180
135
174 CNOT1
75
63
64 YTHDF3
63
55 α-Tubulin

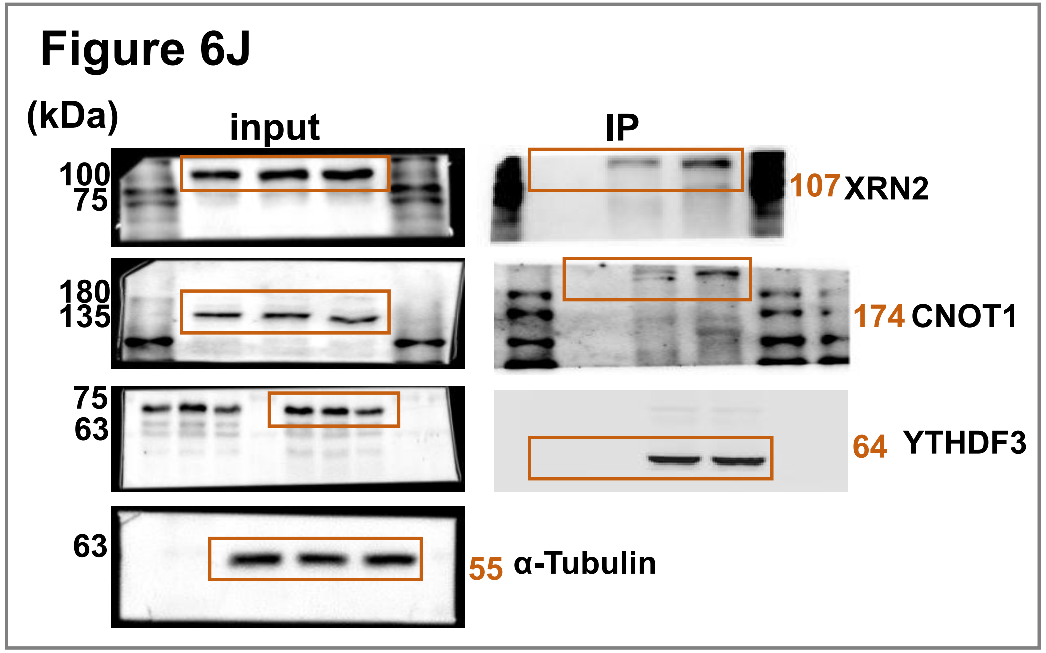
Figure 6J
(kDa)
input
IP
100
75
107 XRN2
180
135
174 CNOT1
75
63
64 YTHDF3
63
55 α-Tubulin

Figure 6K

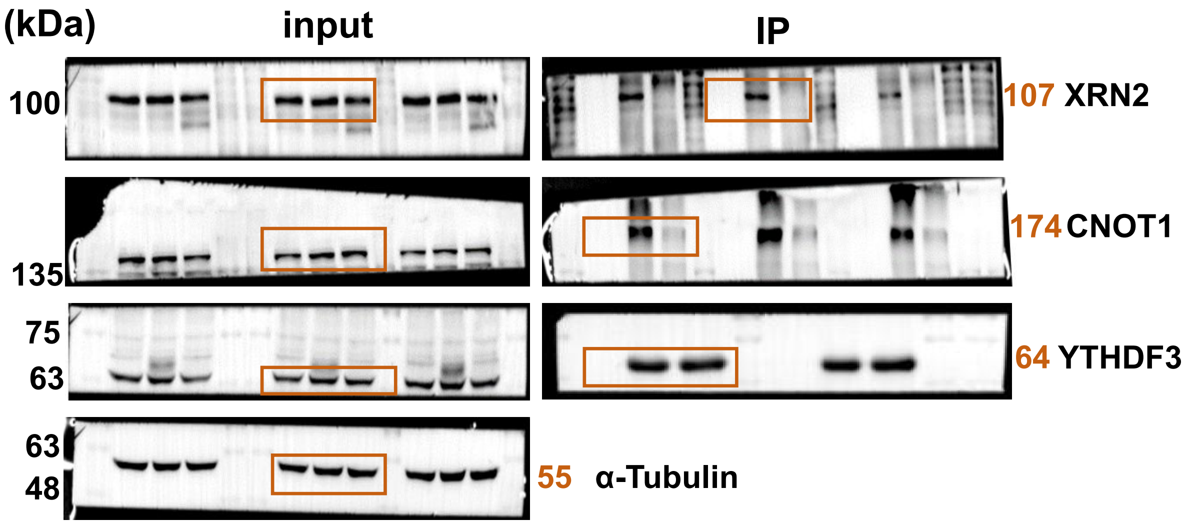

Figure 6L

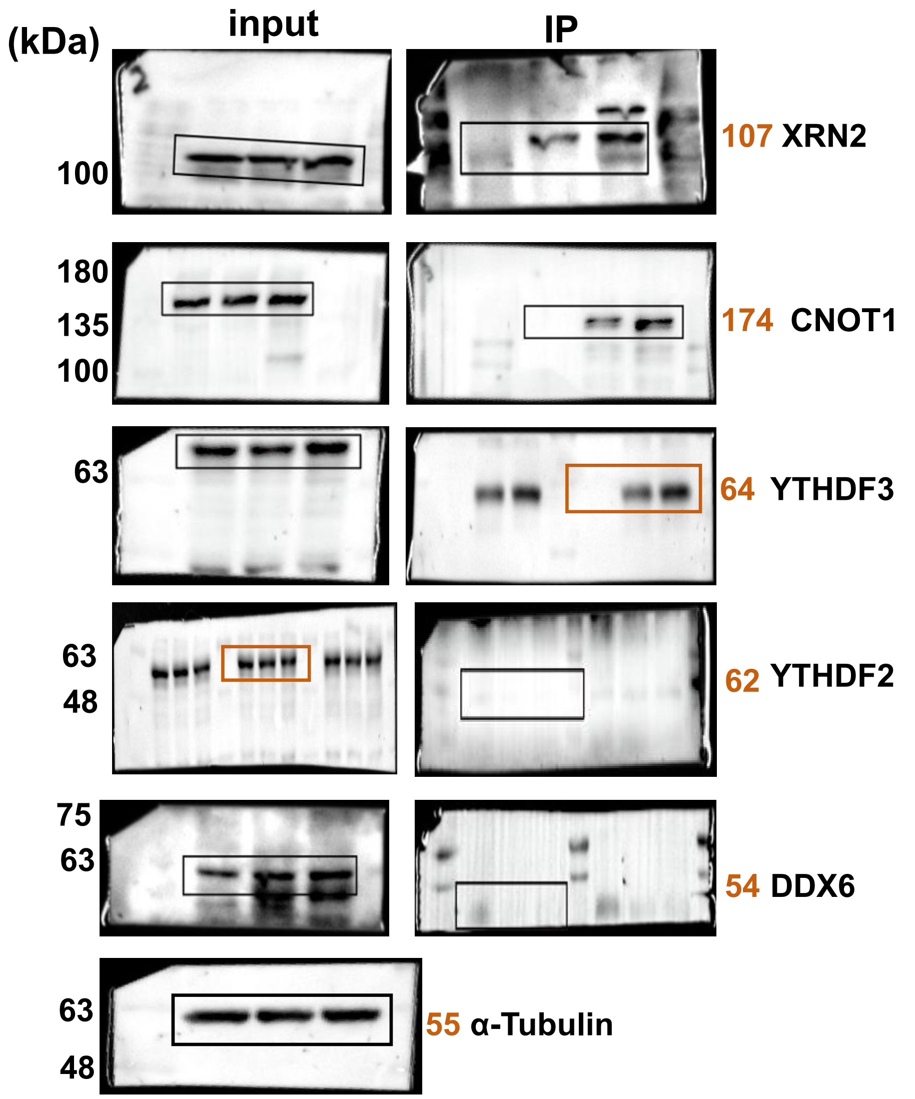

Figure 6N

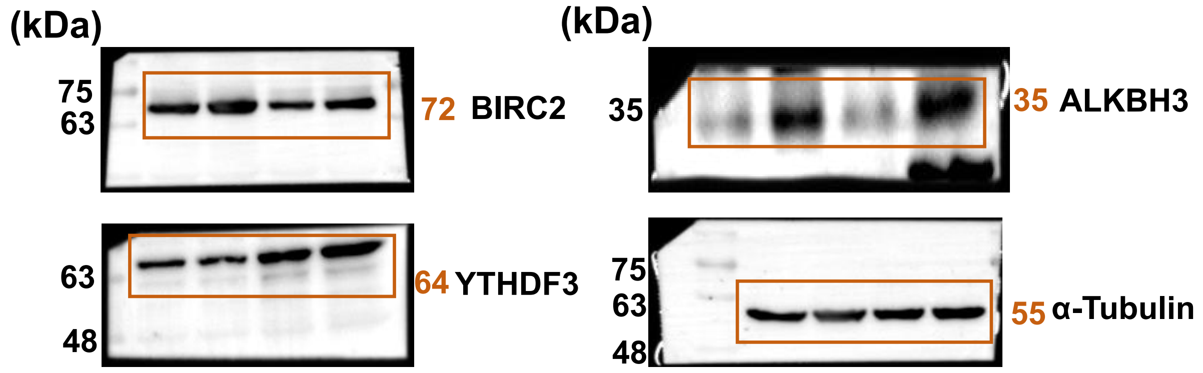

Figure 7H

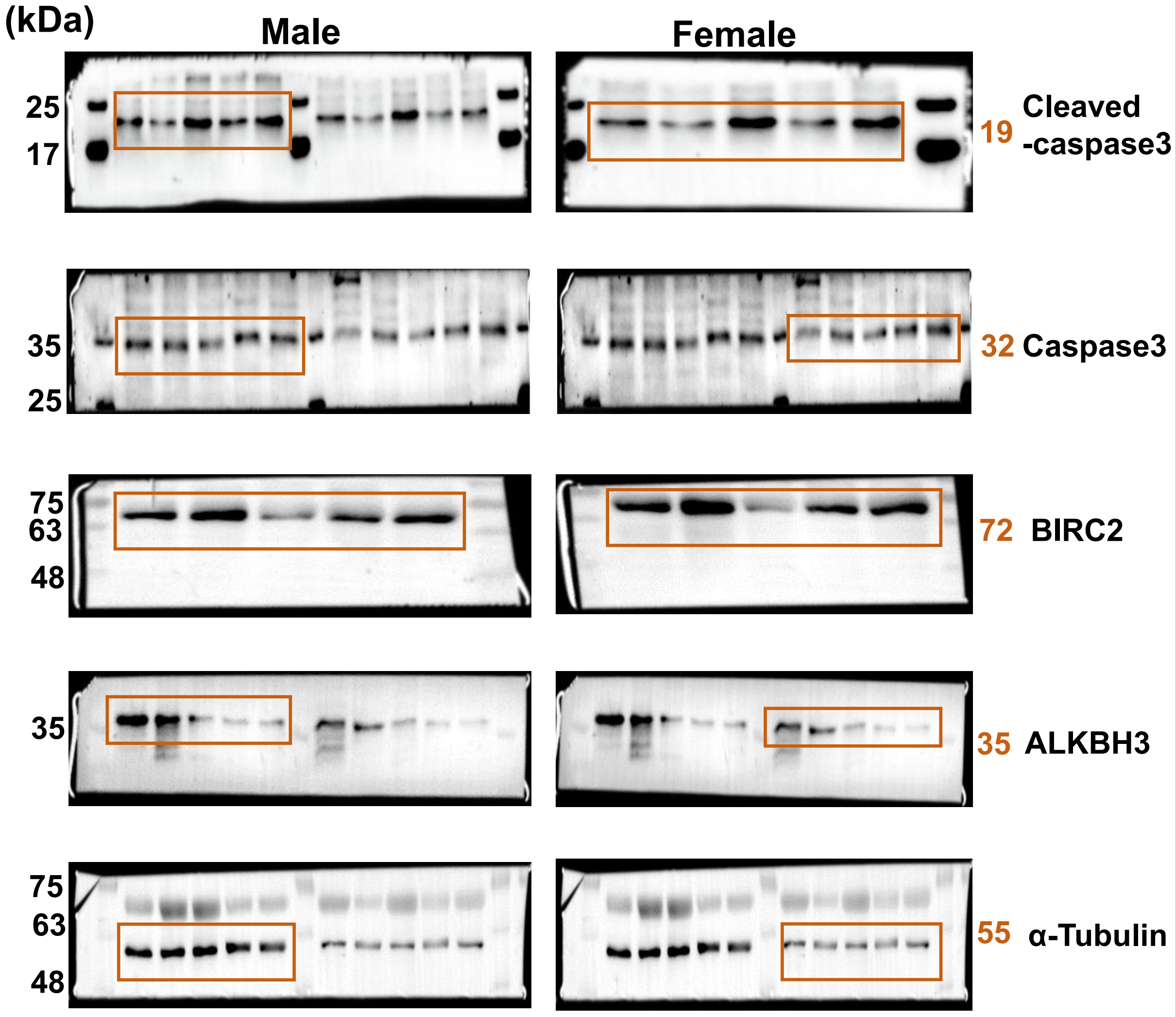

Figure S3B

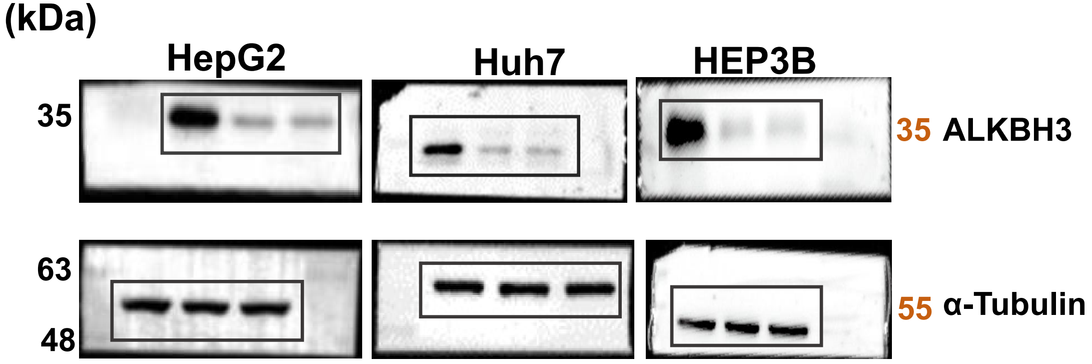

Figure S3E

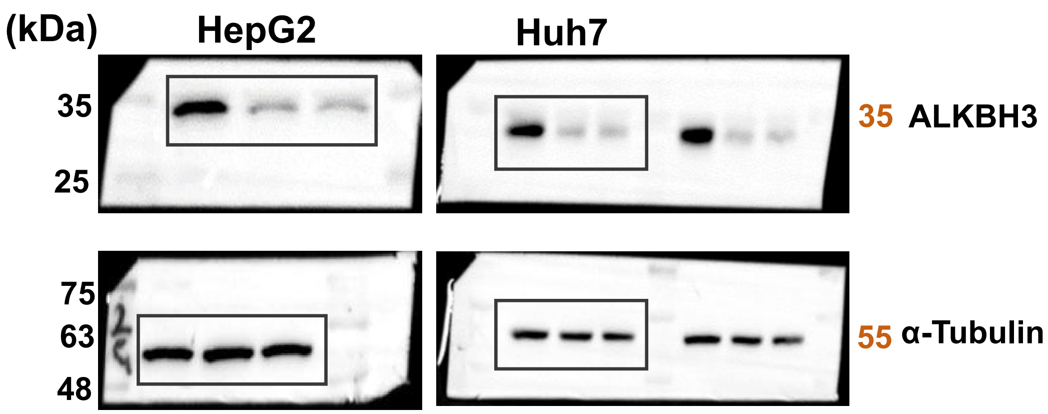

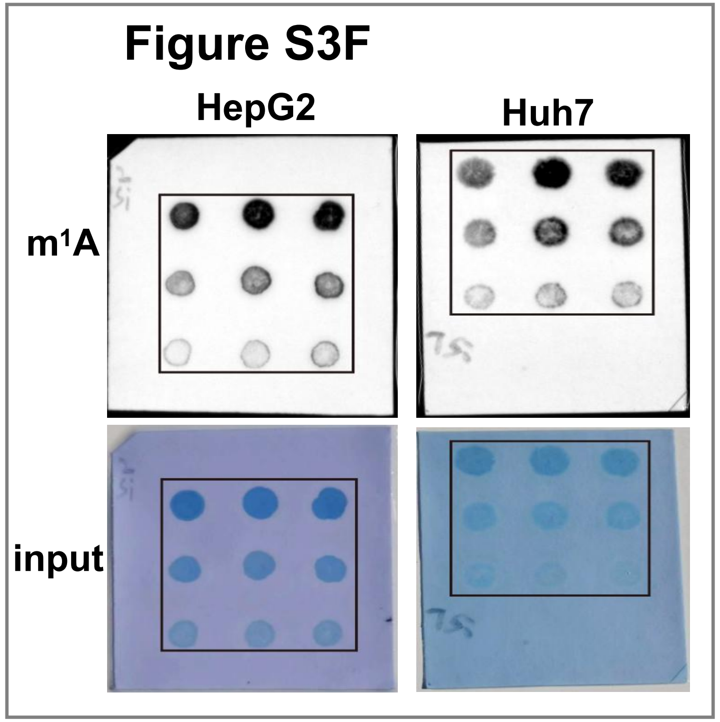
Figure S3F
HepG2
Huh7
m¹A
input

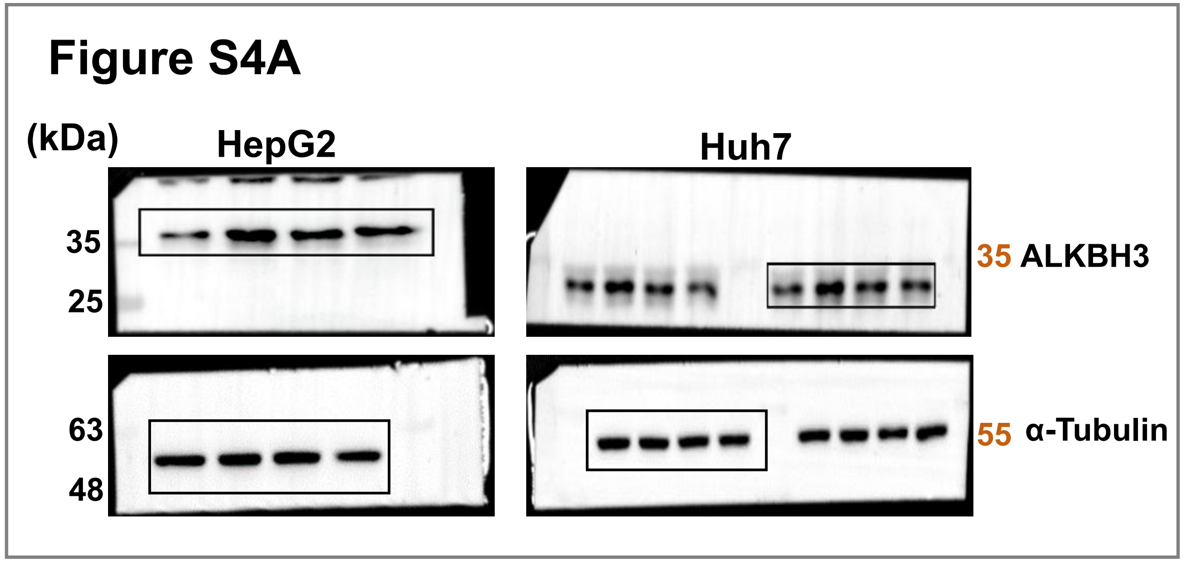
Figure S4A
(kDa)
HepG2
Huh7
35
25
35 ALKBH3
63
48
55 α-Tubulin

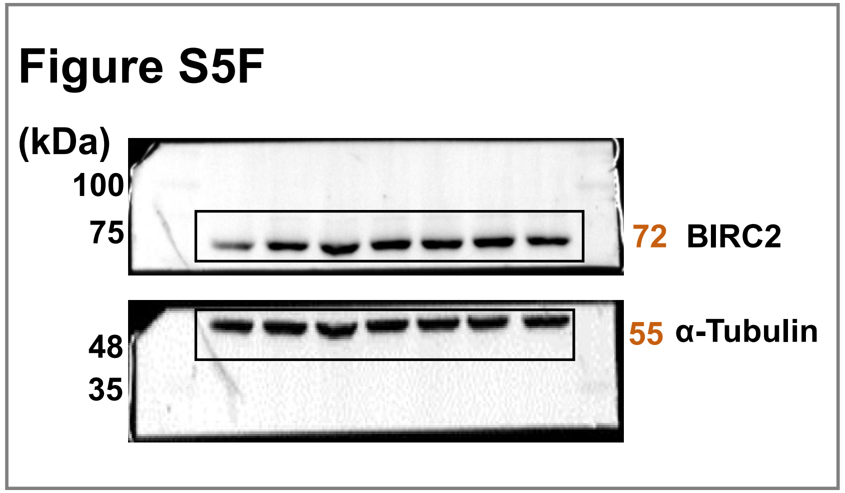
Figure S5F
(kDa)
100
75
72 BIRC2
48
35
55 α-Tubulin

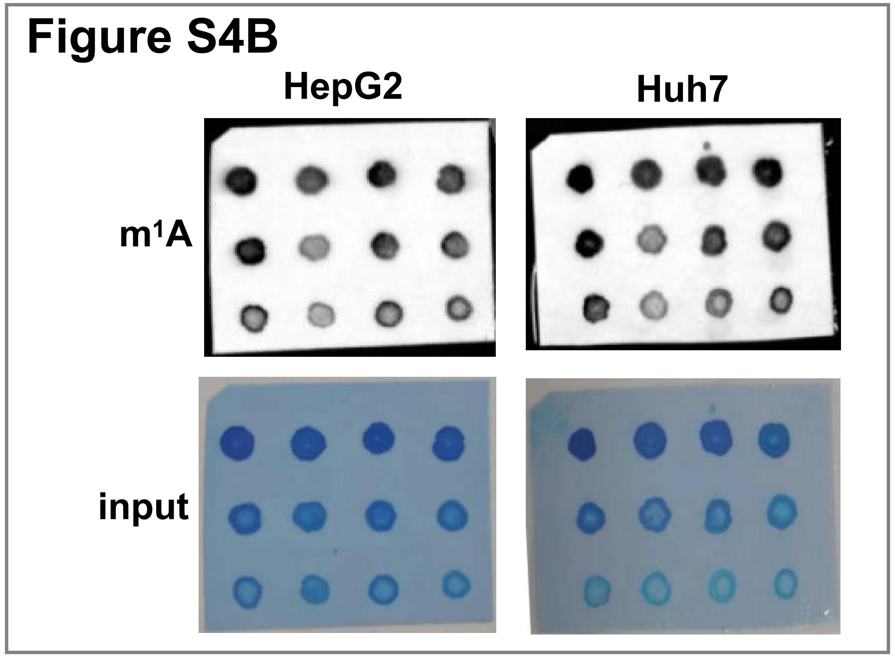
Figure S4B
HepG2
Huh7
m¹A
input

Figure S6A

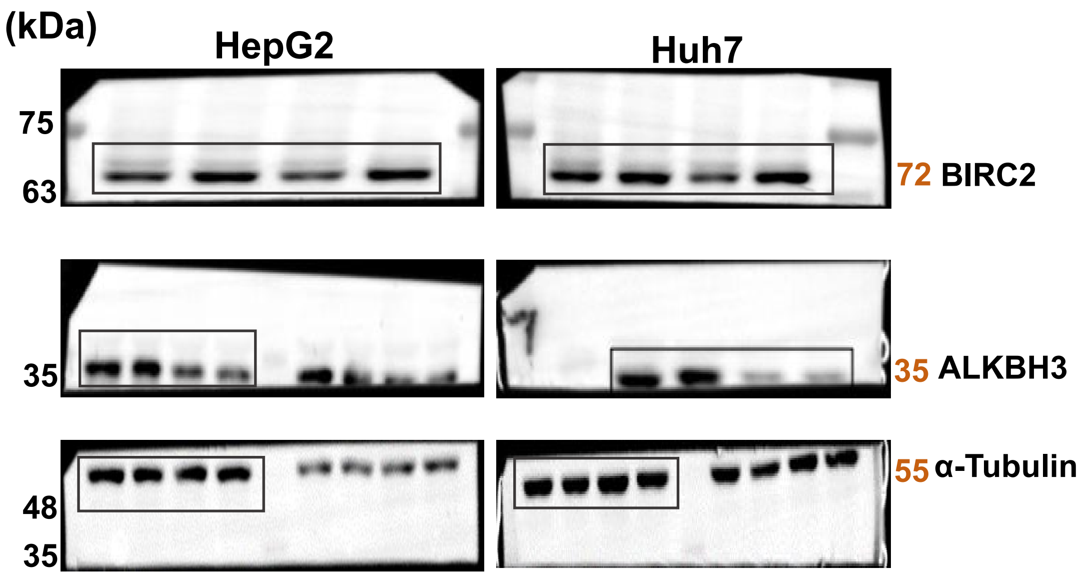

Figure S7E

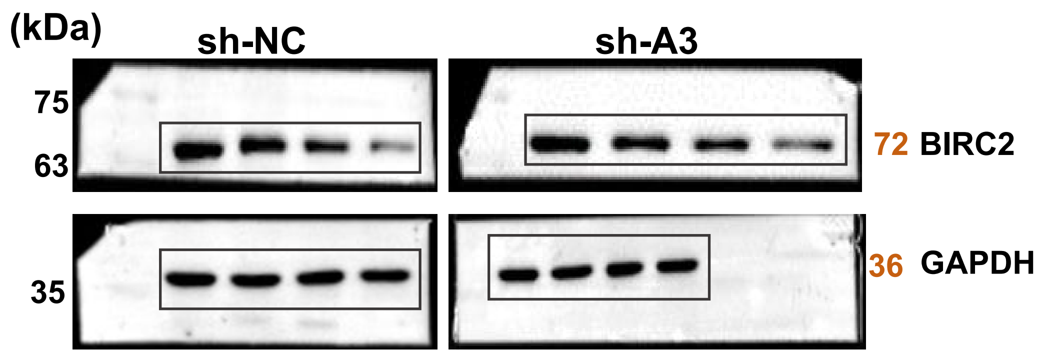

Figure S8G

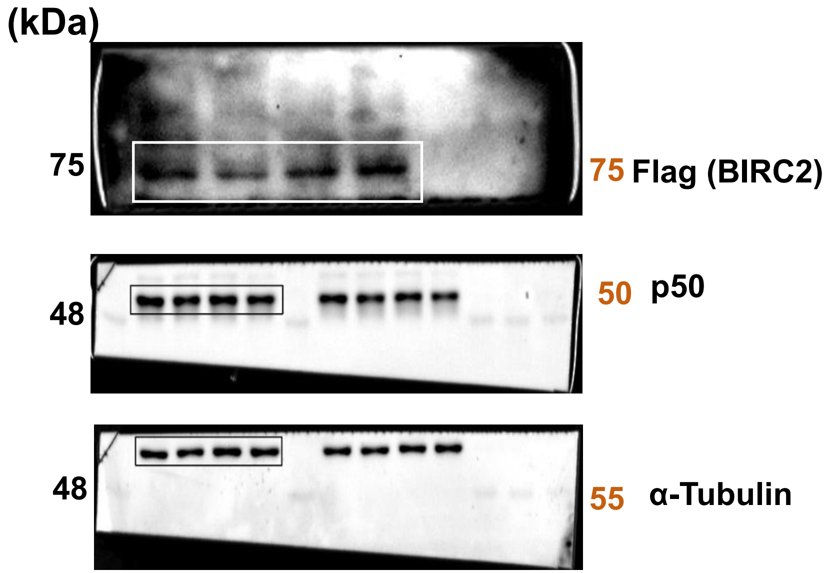

Figure S7F

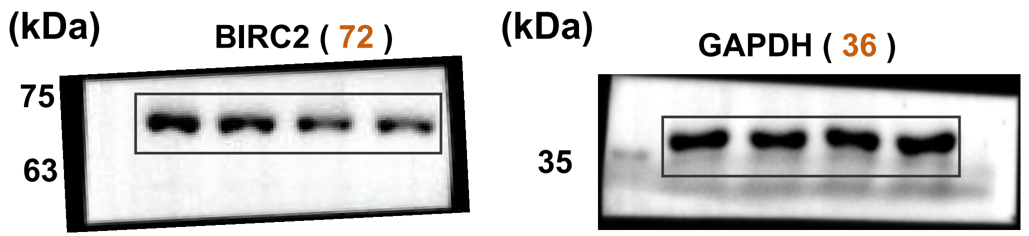

Figure S8K

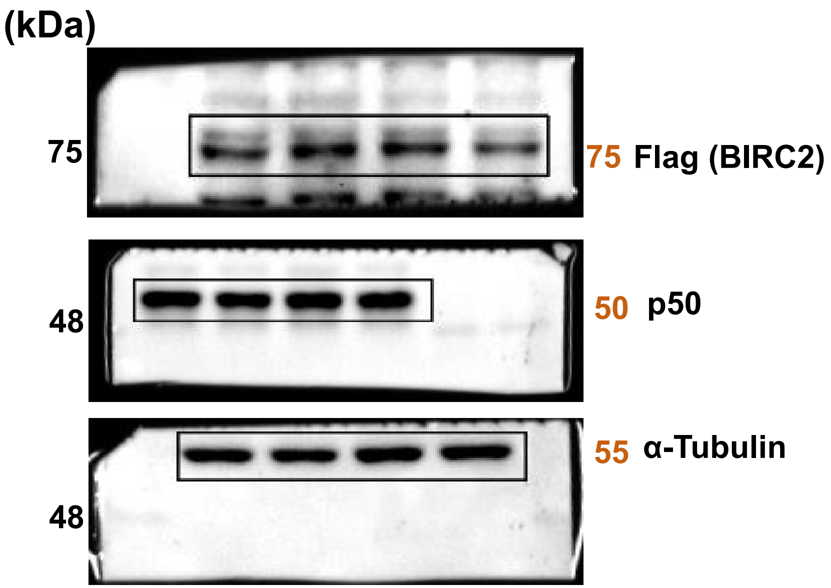

Figure S9C

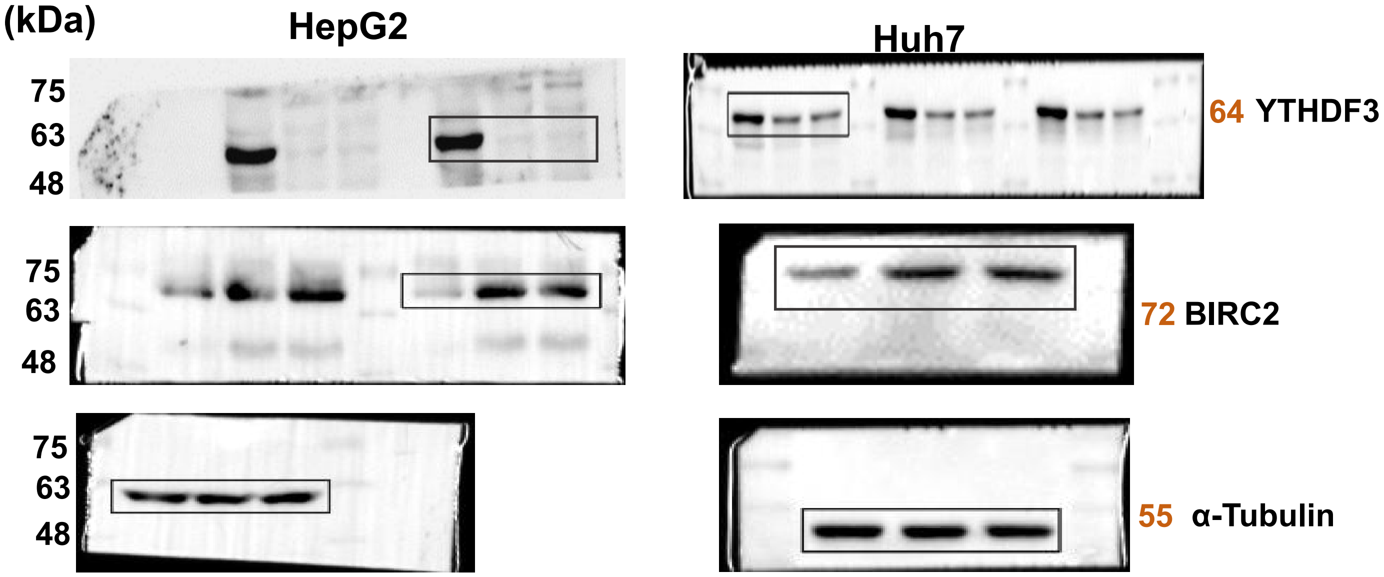

Figure S9E

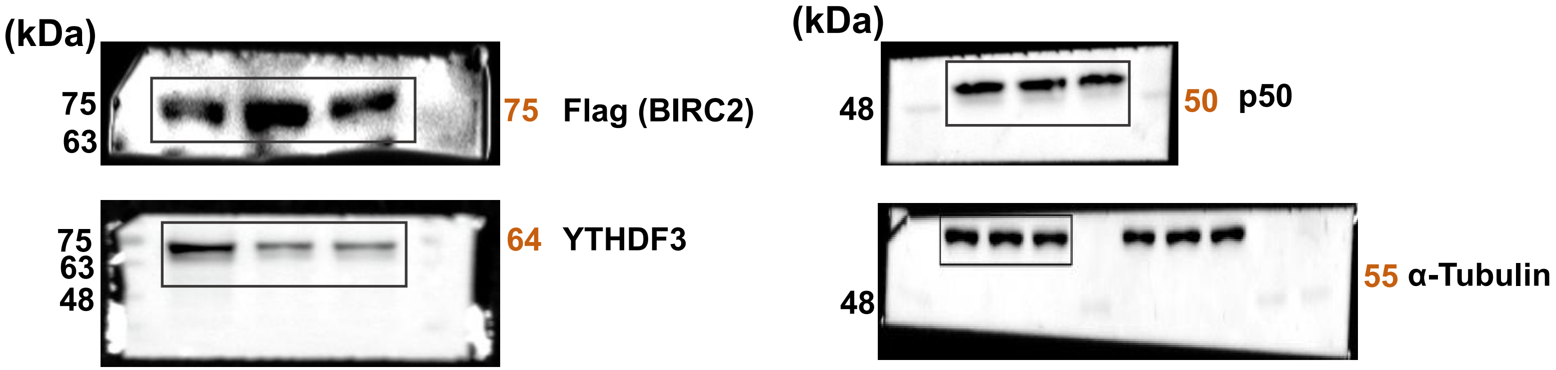

Figure S9J

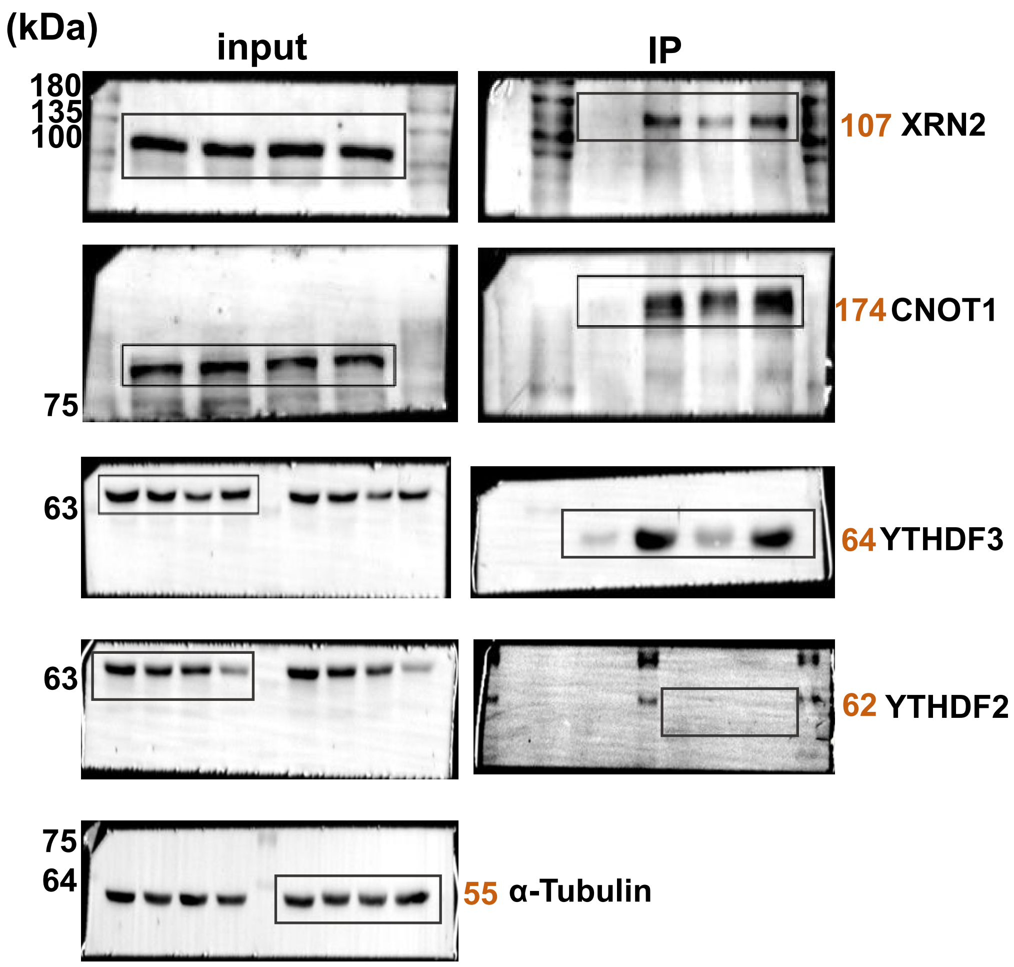

Figure S10D

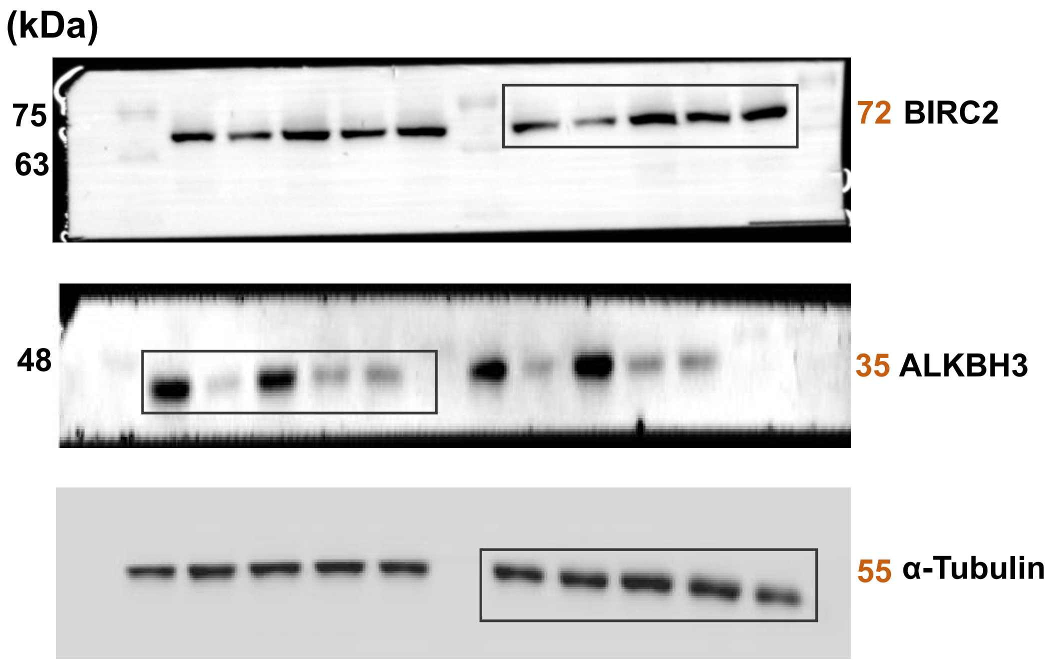

Supplement: Supplementary file 3 — Supplemental Material [file 41419_2026_8731_MOESM3_ESM.pdf]
